# Tag and Snag: A New Platform for Bioactive Natural Product Screening from Mixtures

**DOI:** 10.3390/molecules28155726

**Published:** 2023-07-28

**Authors:** Jeremy Seidel, Yongle Du, Rohin Devanathan, Richard Law, Zhijuan Hu, Nicholas A. Zill, Anthony T. Iavarone, Wenjun Zhang

**Affiliations:** 1 Department of Chemical and Biomolecular Engineering, University of California Berkeley, Berkeley, CA 94720-3220, USA; jeremy.seidel@berkeley.edu (J.S.);; 2 California Institute for Quantitative Biosciences (QB3), University of California Berkeley, Berkeley, CA 94720-3220, USA; 3 QB3—Chemistry Mass Spectrometry Facility, University of California Berkeley, Berkeley, CA 94720-3220, USA

**Keywords:** natural products, pharmacology, mass spectrometry

## Abstract

Natural products provide an unparalleled diversity of small molecules to fuel drug screening efforts, but deconvoluting the pharmacological activity of natural product mixtures to identify key bioactive compounds remains a vexing and labor-intensive process. Therefore, we have developed a new platform to probe the non-specific pharmacological potential of compounds present in common dietary supplements via shotgun derivatization with isotopically labeled propanoic acid, a live cell affinity assay, which was used to selectively recognize the population of compounds which bind tightly to HeLa cells in culture, and a computational LC-MS data analysis of isotopically labeled compounds from cell lysate. The data analysis showed that hundreds of compounds were successfully derivatized in each extract, and dozens of those compounds showed high affinity for HeLa cells. In total, over a thousand isotopically labeled compounds were screened for cell affinity across three separate experiments, resulting in the identification of several known bioactive compounds with specific protein targets and six previously unreported structures. The new natural products include three tulsinol compounds which were isolated from *Ocimum tenuiflorum* and three valeraninium alkaloids from *Valeriana officinalis*. The valeraninium alkaloids constitute a distinct new family of alkaloids from valerian, which may have previously undescribed bioactivity. These results collectively demonstrate the tag and snag workflow’s viability as a drug discovery method.

## 1. Introduction

Before modern pharmacology began with the isolation of morphine from opium in 1803, natural products (NPs) had been used in the form of plant medicines for thousands of years [1,2]. Today, a rich variety of natural products and their derivatives are approved to be used as drugs by the Food and Drug Administration (FDA) [3]. Indeed, natural products and their derivatives still comprise over half of all FDA-approved drugs [3]. Despite this incredible track record of success, natural products have been phased out of drug discovery programs at major pharmaceutical companies because they require laborious purification and repeat assays to isolate bioactive components from their natural source [4]. The process of fractionation and retesting grows exponentially more difficult when attempting to identify compounds without an obvious phenotypic effect, because the more sophisticated assays necessary to detect this bioactivity must be performed on each fraction with an ever-smaller quantity of active compound. To address this mismatch between modern drug discovery methods and the laborious isolation of natural products, there is a pressing need for new tools and strategies to tackle the unique challenges of drug discovery from plants and other natural sources.

Another systemic factor limiting the therapeutic potential of natural products is the pharmaceutical industry’s shift from phenotypic assays to targeted ligand binding assays to drive high-throughput discovery efforts [5]. While this shift to targeted screening has enabled the discovery of less toxic chemotherapies like Imatinib, ligand binding assays against pure protein targets have proven to be unreliable for predicting bioactivity or target ligation in vivo [6]. Furthermore, targeted screening neglects the rich history of natural products as drivers of biological discovery and treats natural products merely as drug candidates. The modern science of aging would have been delayed by decades if rapamycin had not guided researchers to elucidate the molecular mechanisms of growth and aging [7]. The discovery and characterization of mevastatin isolated from *Penicillium citrinum* similarly catalyzed the development of synthetic statin drugs to inhibit cholesterol biosynthesis [8]. As these examples show, natural products have guided researchers to valuable protein targets, paving the way for new therapies and optimized synthetic ligands. Natural products are more than drug candidates; they have been guides to some of the most valuable therapeutic targets known to medical science.

Rather than fighting against the complex character of natural product pharmacology, we endeavored to leverage the inherent diversity of plant metabolomes to boost throughput without neglecting undiscovered therapeutic targets. Reimagining natural product drug discovery to harvest this bounty of diverse molecules required the development of tag and snag, a new workflow for natural product discovery in which we “tag” plant natural products with isotopic labels and then human cells “snag” potentially valuable compounds. Through the cell affinity assay, a diverse array of small molecules are permitted to bind to any protein target, eliminating bias toward cytotoxic compounds or compounds that bind to established pharmacological targets. Rigorous wash steps eliminate weakly bound molecules, which are more likely to have non-specific interactions with the lipophilic components of the cell.

To test this new bioassay, we turned to readily available plant-based dietary supplements as a source of diverse small molecules, albeit any natural product mixtures can be used in our workflow. More than a third of adults report using herbal medicines, plants purported to treat a wide range of conditions from cancer to sleep and mood disorders, despite the fact that there is often little evidence to support these claims [9,10]. Despite the lack of scientific evidence supporting the use of many dietary supplements, many of the drugs we use today originate from ancient herbal medicine traditions, such as the anti-malarial drug artemisinin and the anticancer drug camptothecin [11,12]. Both of these drugs have obvious cytotoxic effects against their respective biological targets, allowing for successful isolation of the natural compounds. However, identifying the active components from herbs with more subtle bioactivity remains a challenge. If a natural product is not cytotoxic, it is likely to fall through the cracks. It is precisely this type of subtle bioactivity, perhaps attributed to a novel mode of action, that the tag and snag workflow is intended to address. Our recent structure–activity–distribution study of anticancer natural products in human cancer cells showed a positive correlation of compound intracellular enrichment to its cytotoxic activity, suggesting the feasibility of using whole cells for bioactive compound enrichment from solutions [13]. Subsequent work developing deuterium adduct-based screening and a similar cell affinity assay to discover a novel anticancer compound has shown that cell affinity is a good filter for bioactive compounds [14]. In this work, we employ the tag and snag workflow to test three well-known herbal medicines: ashwagandha, holy basil, and valerian.

## 2. Results and Discussion

### 2.1. Overview of the “Tag and Snag” Workflow

**Figure 1:**
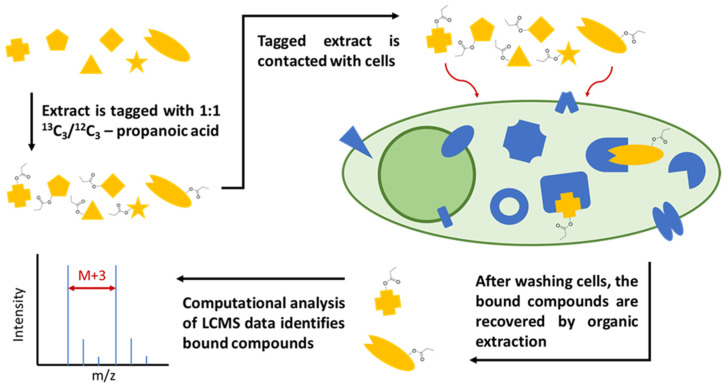
Workflow diagram for the live cell binding assay. First, a crude natural product extract is tagged with isotopically labeled propanoic acid. Then, the tagged extract is incubated with cells. Unbound and loosely bound compounds are washed off the cells, which are then lysed and extracted with chloroform. The cell lysate/extract is then analyzed via LC-MS, after which isotopically labeled compounds can be identified with computational data analysis.

First, a crude natural product extract is tagged with isotope labels for facile compound identification via mass spectrometry. Then, the tagged extract is incubated with human cells such as HeLa cells for potentially bioactive compound enrichment. Unbound and loosely bound compounds are washed off the cells, which are then lysed and extracted with organic solvents such as chloroform to release tightly bound compounds. The cellular extract is then analyzed via liquid chromatography–high resolution mass spectrometry (LC-HRMS) and computational data analysis is used to rapidly identify isotopically labeled metabolites, allowing us to focus on the enriched compounds with a high affinity for cellular targets, which are more likely to have pharmaceutical value. The tag and snag workflow (Figure 1) allowed us to rapidly screen over 1000 compounds from three well-studied plant extracts and efficiently re-identify several known bioactive compounds and also isolate six previously unidentified compounds, as detailed below.

### 2.2. Shotgun Derivatization of Dietary Supplement Extracts

Prior to derivatization, three plant-based dietary supplements were selected and extracted. Ashwagandha (*Withania somnifera*), holy basil (*Ocimum tenuiflorum*), and valerian (*Valeriana officinalis*) dietary supplements were purchased from a local supermarket. All the dietary supplements were advertised as containing freeze-dried plant material from only one species. Extraction in a 2:1 mixture of dichloromethane and methanol was chosen as a means to extract the widest possible array of small molecules, while very hydrophilic molecules relevant to the traditional use of these plants may have been inadvertently excluded by organic extraction. On the other hand, the organic extraction may recover molecules excluded by aqueous extraction that possess bioactivity unrelated to traditional use. An initial LC-MS-based analysis of the extracts demonstrated that they all contained ions that corresponded to characteristic compounds from the species indicated on the label.

Selecting an appropriate chemical reaction for the shotgun derivatization was crucial to tag many different species in the extract with high efficiency and minimal chemical degradation. Nucleophilic groups represent accessible targets for derivatization, including primary and secondary amines, alcohols, phenols, and thiols. While amines and thiols have the advantage of being easy to modify in aqueous solutions, they are not present on the majority of small-molecule secondary metabolites. The most abundant nucleophilic functional groups on natural products are alcohols and phenols, which are present on ~54% of natural product-derived drugs [15]. We chose to use Steglich esterification for the shotgun derivatization because it can acylate alcohols and phenol groups under mild conditions. The crude natural product extract was tagged with equivalently normal and isotopic propanoic acid for facile-tagged compound identification via computational analysis. ^13^C_3_-labeled propionic acid was chosen as the tag because it presented the optimal combination of small size and easy detection in computational LC-MS data analysis. The isotopic label was determined to require at least an M+3 character because M+1 and M+2 peaks can be quite pronounced in small-molecule natural products due to endogenous ^13^C atoms. Conversely, endogenous M+3 peaks are typically of low intensity even for large small molecules, up to 1500 *m*/*z*. It would have been possible to use D_3_-acetic acid to achieve an even smaller label; however, carbon–deuterium bonds are significantly more polar than carbon–hydrogen bonds. This causes a retention time shift in deuterium-labeled compounds relative to their unlabeled counterparts, which would have hindered accurate data analysis.

While it is possible that acylation at a critical functional group may alter bioactivity, there remain many examples of acylated natural products which retain their bioactivity, such as morphine (diamorphine), erythromycin, and betulinic acid (bevirimat), etc. [16]. The enrichment of untagged compounds in reporting cells could be readily confirmed following tagged compound identification. However, some compounds will inevitably be missed in our assay if acylation leads to a complete abolishment of binding. Indeed, our results show that one of the reported compounds, valeraninium C, demonstrates the complete abolishment of bioactivity after uptake. This is an example of how bioactivity modification through acylation may be beneficial if it provides information about which functional groups are critical to cell affinity and bioactivity. Furthermore, the identified bioactive natural products already contain a modifiable moiety, which facilitates subsequent lead optimization and molecular target identification.

To fully characterize the effectiveness of the shotgun derivatization, it was necessary to implement an automated data analysis pipeline. MS-DIAL was used to generate a peak table from the raw LC-MS data. A subsequent analysis of the peak table in Python identified peaks expressing the isotopic label based on peak intensity, retention time, and mass-to-charge ratio (*m*/*z*) values. Following the Python analysis, results were visually checked to confirm the low frequency of false positives. All the tagged extracts contained hundreds of isotopically labeled compounds. The extract with the fewest tagged compounds was valerian, in which 270 hits were revealed, while the extract with the most tagged compounds was holy basil, in which 661 hits were found. A computational analysis of an untagged extract returned less than a dozen false positive results, demonstrating the robustness of our data analysis methods.

### 2.3. NP Enrichment by Reporting Cells and MS Identification

During the cell affinity assay, the tagged dietary supplement extracts were incubated with HeLa cells, which were subsequently passed through a rigorous washing process. The strong-binding compounds were re-isolated from these cells through chemical extraction followed by LC-HRMS detection and computational data analysis. The isotopic feature of the tag readily distinguished NPs from the endogenous metabolites of Hela cells, and these isotopically labeled compounds were identified using the same computational procedure used to validate the initial shotgun derivatization. Each labeled extract contained between 250 and 700 isotopically labeled compounds, from which about 4% to 11% compounds present in the extract were enriched and re-identified after the cell affinity assay (Tables S1–S3, Supplementary Materials). The whole cell chemical extraction may miss covalently bound compounds, and we here thus primarily focus on non-covalently bound compounds as they are more challenging to be identified through traditional assays and most active NPs probably fall in this category [14]. Interestingly, several identified ions could not be found from the pre-incubation mixture, suggesting that some of the parent-tagged compounds were metabolized by HeLa cells during incubation. Many of the identified ions were predicted to belong to the same family of natural products using the MS fragment-based molecular network analysis. Based on the enrichment level and the parent-untagged compound abundance in the original crude extract, we then selected a few represented parent compounds for in-depth structural characterization (Table 1).

### 2.4. Structural Elucidation of Selected Natural Products from Ashwagandha

**Figure 2:**
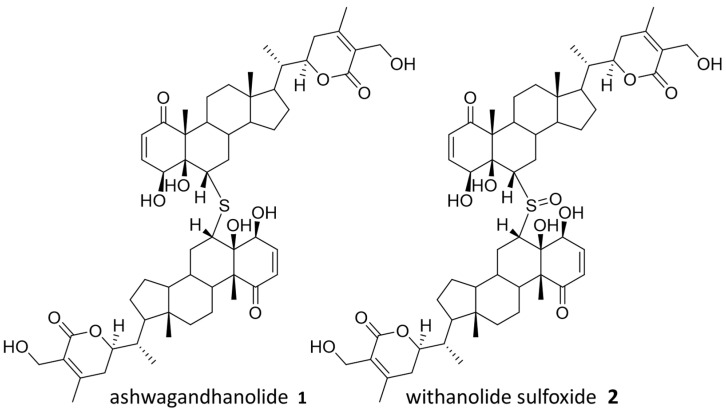
Identified natural products from Ashwagandha.

*Withania somnifera*, also known as Ashwagandha, is commonly used in Ayurvedic medicine as an anti-anxiety and fitness supplement, although there is no scientific evidence to support these uses [17]. Ashwagandha is a source of several known bioactive compounds, and ashwagandhanolide (**1**), previously isolated with anticancer activity, was successfully identified through our “Tag and Snag” workflow [18,19]. Specifically, an untagged parent ion of *m*/*z* = 975.5297 (predicted formula C_76_H_78_O_12_S:H^+^) was identified and the corresponding compound was purified and confirmed to be ashwagandhanolide (**1**) by nuclear magnetic resonance (NMR) spectroscopy analysis (Figure S6, Supplementary Materials). Meanwhile, we identified a less abundant parent ion of *m*/*z* = 991.5257 (predicted formula C_76_H_78_O_13_S:H^+^) to be the known dimeric steroid withanolide sulfoxide (**2**) based on the observed exact mass. Both compounds are known to exert their anticancer effects by the selective inhibition of cyclooxygenase-2 [18,19], and the anticancer activity of **1** has also been confirmed by sulforhodamine B (SRB) assays (Figure S19). The rediscovery of these bioactive compounds validates our workflow by demonstrating the ability to discover compounds from NP mixture with potential therapeutic values. Compounds from Ashwagandha that were identified in the cell affinity assay are shown in Figure 2.

### 2.5. Structural Elucidation of Selected Natural Products from Holy Basil

**Figure 3:**
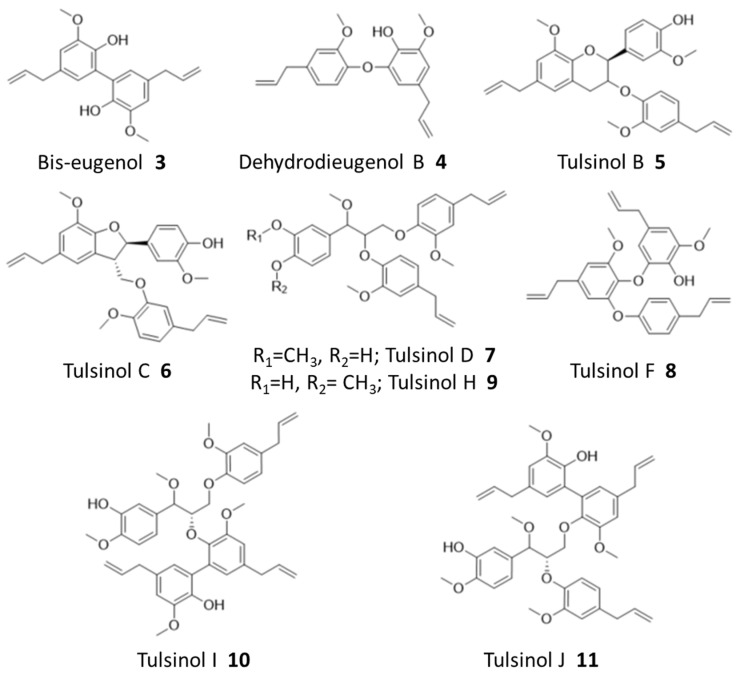
Identified natural products from holy basil.

*Ocimum tenuiflorum*, also known as holy basil, is a known producer of various terpenoid and lignan natural products, and it was used for antibiotic, immunomodulatory, and analgesic effects [20,21]. Meanwhile, it is worth noting that many natural products have been isolated from *O. tenuiflorum*, and it possesses immense biosynthetic capabilities within its genome, including 81 putative terpene synthase gene clusters [22,23]. Therefore, it is understandable that holy basil showed the largest number of derivatized compounds, both in the extract and after the cell affinity assay. Compounds from holy basil that were identified in the cell affinity assay are shown in Figure 3.

Ions corresponding to bis-eugenol (**3**) and dehydrodieugenol B (**4**), as well as several known members of the tulsinol family, including tulsinols B (**5**), C (**6**), D (**7**), and F (**8**), were successfully identified during the binding assay, and some of their structures were further confirmed by NMR analysis (Figures S7–S12). Among these compounds, bis-eugenol (**3**) has been demonstrated to exhibit antioxidant properties as well as anti-inflammatory activity mediated through the suppression of NF-kB activation; while tulsinol C (**6**) has been reported to have a good antiparasitic activity against *Leishmania normalis* [20,24]. No direct anticancer activity has been reported for these compounds nor based on our SRB assays (Figure S19, Supplementary Materials), demonstrating the capability of our workflow to reveal potentially bioactive compounds beyond cytotoxicity.

While natural products from holy basil have been well-studied, our workflow further identified several new compounds which are reported here for the first time based on our best knowledge. Specifically, an NMR analysis of purified compounds corresponding to ions 538.2796 (predicted formula: C_34_H_40_O_9_:NH_4_^+^) and 705.3040 (predicted formula: C_41_H_46_O_9_:Na^+^), respectively, revealed three new tulsinol families of natural products, which we named tulsinol H–J. Tulsinol H (**9**) is an isomer of tulsinol D (**7**), differing in one methylation position, and tulsinols I (**10**) and J (**11**) are isomers which each possess a bis-eugenol moiety attached to different parts of the shared tulsinol D scaffold (Figures S13–S15). Notably, these compounds also did not demonstrate cytotoxicity in SRB assays (Figure S19). The discovery of tulsinol H–J suggested that our workflow was particularly efficient in identifying potentially bioactive compounds that were missed during the traditional NP discovery workflows, such as those based on phenotypic screening.

### 2.6. Structural Elucidation of Selected Natural Products from Valerian

*Valeriana officinalis*, also known as valerian, is a popular dietary supplement derived from the root and advertised as an anti-anxiety supplement and a potential treatment for sleep disorders [25,26]. Valerian root is known as a source of iridoid pyridiniums and sesquiterpenoids such as valerenic acid, although the contributions of these substituents to the overall health benefits remain unclear [27,28]. We selected a pair of isomeric hit compounds with the same predicted molecular formula (C_33_H_44_NO_3_^+^) for structural elucidation, as multiple database searches suggested that they might be new compounds never reported before. Unfortunately, although significantly enriched in our cell affinity assays, the parent-untagged compounds in the original extract were too dilute to be purified with a large-enough amount for structural elucidation using NMR.

**Figure 4:**
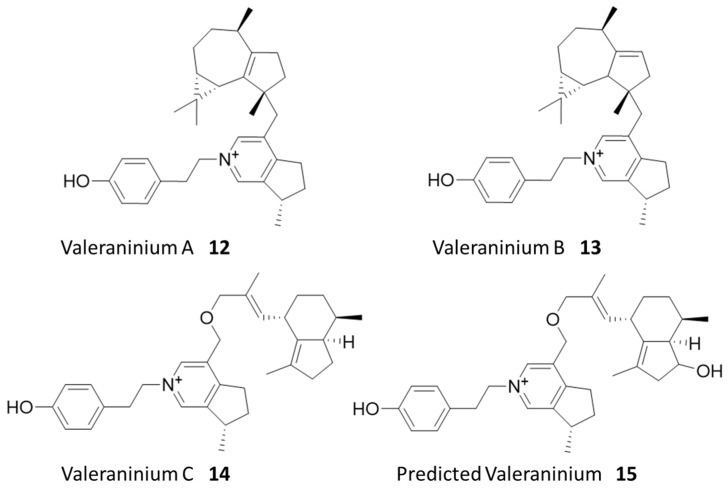
Identified natural products from valerian.

Considering that similar metabolites are typically produced as compound families in plants, we performed the molecular network analysis to identify related untagged parent metabolites, which are more abundant in the original extract, but may not be revealed from our cell enrichment assay due to a lack of hydroxyl for tagging or a loss of activity upon tagging. Three new compounds were successfully identified using this method, for which we managed to purify enough materials for structural elucidation using NMR analysis. These three compounds were predicted to have molecular formulas C_33_H_44_NO^+^ and C_33_H_44_NO_2_^+^ based on HRMS analysis. They were revealed to be a family of new alkaloids, which we named valeraninium A (**12**), B (**13**), and C (**14**), respectively, sharing a common iridoid pyridinium moiety linked to a variable sesquiterpenoid moiety, as shown in Figure 4 (Figures S16–S18). These novel alkaloids are united by an n-para-ethylphenol valeranine base first isolated and characterized by Torsell and Whalberg in 1967 [27]. These appear to be mature forms of that alkaloid and possess a variety of terpenoid groups attached at the ethoxy group by an ether bond. The terpenoid groups identified here include gurjenene, isoledene, and valerenic acid [29,30,31]. The compound (**15**) detected in the affinity assay is predicted to be a variant of valeraninium C with a hydroxyvalerenic acid terpenoid group using MS/MS analysis (Figure S20, Supplementary Materials). Again, these compounds also did not demonstrate obvious cytotoxicity in SRB assays up to 10 µM (Figure S17).

Interestingly, only the predicted compound **15** and its isomers were successfully identified during the initial cell affinity assay (Table S3). Six prominent peaks sharing the formula for compound **15** were identified in the pre-binding mixture, along with three tagged variants. After the cell affinity assay, four out of six untagged variants, along with two tagged isomers of **15,** were detected (Figure S21). These tagged variants may be independent derivatives of separate isomers, or isomers generated by modifying a single isomer at either the known phenol group or putative hydroxyl group.

Of compounds **12**–**14**, only compound **14** was confirmed to be successfully tagged in the pre-binding mixture (Figure S21). Given the similar structure of the family of compounds, this is a striking difference in detected cell affinity. One possible explanation is that the acylation of the phenol group diminishes its compatibility with a hypothetical protein target. To test this, we generated an extracted ion chromatogram (EIC) for compound **14** and its tagged variants. The chromatogram shows that only the untagged compound **14,** as well as the untagged compounds **12** and **13,** are present after the cell affinity assay, indicating that the acylation of the phenol group negatively impacted cell affinity. On the other hand, the detection of tagged **15** after the binding assays suggested that the acylation of hydroxyvalerenic acid was tolerated for cell affinity. Previous work established that valerenic acid and hydroxyvalerenic acid exerted their anxiolytic effect by binding to a subset of GABA-A receptors and allosterically modulating their activity [32]. Further work showed that the acylation of hydroxyvalerenic acid still bound to GABA-A receptors but could not modulate their activity [33]. Additionally, GABA-A receptors have been found in human cervical tissue, indicating that this protein target is likely expressed in HeLa cells [34]. These findings are consistent with our observed binding of **15** derivatives to cells, likely via specific protein targets. The tag and snag workflow thus spontaneously generated a structure-cell affinity relationship experiment for this family of compounds, demonstrating the power of the multifaceted data produced by the combination of shotgun derivatization and cell affinity assay. Future work to confirm the structure of **15** and elucidate the protein targets of **14** and **15** may help expand our understanding of valerian’s demonstrated anxiolytic and sedative effects.

## 3. Materials and Methods

### 3.1. Dietary Supplement Extraction and Validation

The plant-based dietary supplements were extracted with a 2:1 ratio of dichloromethane and methanol for 18 h to extract the full spectrum of secondary metabolites. Because the shotgun derivatization is performed via Steglich esterification, it was essential to ensure that the dietary supplement extracts were completely free of water [27]. First, the dichloromethane/methanol extract was fully evaporated under nitrogen until an opaque, oily residue was obtained from each sample. This residue was subsequently redissolved in anhydrous tetrahydrofuran and concentrated down to a translucent, water-free residue. After determining the mass of each individual residue, the various samples were redissolved in anhydrous tetrahydrofuran to a constant concentration of 50 mg/mL.

### 3.2. Shotgun Derivatization of Dietary Supplements with ^13^C_3_/^12^C_3_-Propanoate Tag

To perform the shotgun derivatization, the anhydrous extracts were combined with dimethylaminopyridine (DMAP), diisopropylcarbodiimide (DIC), and a 1:1 ratio of ^13^C_3_/^12^C_3_-propanoic acid using anhydrous tetrahydrofuran as the solvent. After 24 h, the reaction was terminated and evaporated under nitrogen. DMAP and diisopropylurea, produced by consuming DIC, remained in the tagged extracts throughout this workflow, but were not concentrated enough to exert cytotoxicity, much less alter the physical properties of the cells in culture. After redissolving the tagged extracts in methanol, the samples were analyzed by LC-MS. A manual review of the tagged extract LC-MS data showed many compounds expressing the M+0 and M+3 isotopic abundance pattern, so it was immediately apparent that the shotgun derivatization had been successful.

### 3.3. Cell Affinity Assay Procedures

After the extracts were tagged with ^13^C_3_/^12^C_3_-propanoic acid, they were prepared for use in the cell affinity assay by fully evaporating the methanol under nitrogen and redissolving the residue in dimethylsulfoxide (DMSO) to a constant concentration of 100 mg/mL. During the cell binding assay, each labeled extract in DMSO was added to cell culture medium to a final concentration of 1 mg/mL. The final concentration of DMSO itself was limited to 1% to prevent confounding effects on the binding assay. The HeLa cells were then incubated with an extract-rich medium for two hours. During this time, high-affinity compounds concentrated within the cells. The extract-rich medium was then replaced with fresh medium, and the cells were allowed to incubate for two more hours to eliminate non-binding and weakly bound compounds from the cells. Three consecutive wash steps were performed, each including 5 min of incubation time with 10 mL phosphate-buffered saline (PBS), to thoroughly wash the growth medium off the cells and remove any lingering weakly bound compounds. Cells were recovered by scraping into 2 mL PBS, and the suspension was extracted using chloroform. This extract was concentrated and re-dissolved in 100 µL of methanol for LC-MS analysis.

### 3.4. LC-MS Analysis

LC-MS analysis of the tagged extract and cell binding assay samples was originally performed on a Thermo Fisher Q Exactive Orbitrap mass spectrometer coupled to ultra-performance liquid chromatography (UPLC). For each dietary supplement, 100 µg of tagged extract dissolved in 10 µL of methanol and 10 µL of the methanolic cell binding assay sample were injected separately. The solvent system was acetonitrile (ACN) and water, each with 0.1% formic acid. Chromatographic conditions were a 0.1 mL/min flow rate starting at 2% ACN for 10 min, followed by a 30 min gradient up to 98% ACN and a 10 min hydrophobic wash at a constant 98% ACN.

### 3.5. Computational Data Analysis

**Figure 5:**
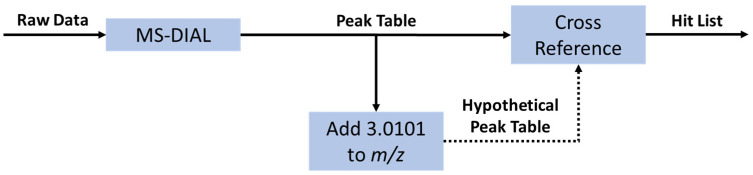
Flow chart for the isotopic label detection data analysis in using MS-DIAL ver.4.70 and Python. The first step is the generation of a peak table using the open-source software MS-DIAL. The Python data analysis is initiated by creating a hypothetical table of peaks with exactly the mass difference between ^12^C_3_- and ^13^C_3_-propanoic acid (+3.0101 *m*/*z*). This hypothetical table was then compared against the original table to find peaks with similar *m*/*z* (±3 ppm), retention time (±0.05 min), and intensity (up to 33% difference). The pair of peaks, both from the original peak table, was then collected in a hit table containing the *m*/*z*, retention time, and intensity of both peaks.

Following Orbitrap analysis, the Thermo Fisher .RAW files were converted to .mzML format using the MSConvert graphical user interface (GUI) and subsequently processed in MS-DIAL using the default parameters. The resulting peak tables were then exported to .csv files and analyzed in Python. The Python script used can be found in the supplementary information. A flow chart describing how the python script works is shown in Figure 5.

The initial isotopic label detection was performed in the same manner for the labeled extract both before and after the cell affinity assay, generating two independent hit tables. The two resulting peak tables were then compared to find peaks with similar *m*/*z* (±4 ppm) and retention time (±0.5 min). Hits that were present both before and after incubation with cells were considered for isolation and characterization.

### 3.6. Extraction and Isolation

All herbal supplements were extracted in methanol and individual compounds were isolated by high-performance liquid chromatography (HPLC) in water and acetonitrile. Full details of extraction and isolation can be found in the supplementary information.

### 3.7. Structural Characterization

The structures of tulsinols H, I, and J were determined by analysis of ^1^H NMR, heteronuclear single quantum correlation (HSQC), and heteronuclear multiple-bond correlation (HMBC) spectroscopic data after the molecular formulae were determined by HRMS. Full details for the structural characterization of novel tulsinols can be found in the supplementary information.

The structures of valeraninium A, B, and C were determined by analysis of ^1^H NMR, HSQC, HMBC, and correlation spectroscopy (COSY) spectroscopic data after the molecular formulae were determined by HRMS. Full details for the structural characterization of novel tulsinols can be found in the supplementary information. All structures contained an iridoid pyridinium moiety as indicated by known reference [27]. The structures of the terpenoid moieties were attached either through carbon–carbon bond (valeraninium A and B) or ether bond (valeraninium C). The structures of the terpenoid moieties were confirmed by comparison to known references [28,30,35]. Full details for the structural characterization of the valeraninium alkaloids can be found in the supplementary information.

### 3.8. Cytotoxicity Assay

The cytotoxicity of the isolated compounds against MCF-7 human breast cancer cell line was evaluated using Cyto Scan^TM^ SRB cell cytotoxicity assay kit according to manufacturer’s instructions. Compounds were screened with a single dose (10 mM) while IC_50_ value of compound **1** was further determined. Briefly, MCF-7 cells were seeded in 96-well plate at 5000 cells/well confluence for 24 h, followed by treatment with test compounds at appropriate concentrations. After further 72 h incubation at 37 °C with 5% CO_2_, 50 mL fixative reagent was gently layered onto each well. The plate was incubated at 4 °C for 1 h, washed 4 times with distilled water, and dried in a 50 °C incubator for 30 min. Then, 100 mL of SRB dye solution was added and incubated for 30 min at room temperature in the dark. The wash and dry steps were repeated with wash solution instead of water. The dye was dissolved with 200 mL SRB solubilization buffer and the absorbance was measured at 515 nm. DMSO was used as a negative control. Absorbances were blanked with cell-free medium controls. % Cytotoxicity = (100 * (Cell Control-Experimental))/Cell Control. IC_50_ was calculated using GraphPad Prism 9.

## 4. Conclusions

We have demonstrated the “Tag and Snag” workflow by identifying natural products, some of which are new, from well-studied dietary supplements. The live cell binding and enrichment assays have been employed for primary bioactive NP screening, a method which has shown higher flexibility and translatability than the in vitro purified target-based screening, and higher sensitivity and flexibility than the traditional phenotype-based screening. As a result, compounds which were missed during previous studies, including a new family of valeraninium natural products from valerian, were revealed from our workflow. While the particular bioactivity and molecular target of identified compounds warrant further study, we have provided a list of derivatizable natural products which are suitable for subsequent protein target identification, promoting the future bioactivity investigation of natural products and their potential therapeutic values. Indeed, the facile derivatization of identified compounds is an advantage as many protein target identification methods require a modification of the drug [36]. The tag and snag workflow therefore offers to integrate natural product discovery and target identification into an efficient pipeline.

## Figures and Tables

**Table 1:** Representative examples of ions identified for characterization. Three dietary supplements were used, and compounds were selected for isolation and characterization based on the cell affinity assay. Compounds from holy basil included two sets of isomers. The compound identified from valerian consisted of two isomers generated by tagging a single-parent molecule.

Source	Tagged *m*/*z* (M+0)	RT (min)	Mass Intensity	Calc. Parent *m*/*z*	Adduct	Calc. Mass	Structure	LC-MS Figure
Ashwag.	1031.5559	24.5	4.32 × 10^4^	975.5297	H^+^	974.5214	**1**	Figure S1
Ashwag.	1047.5519	22.3	8.09 × 10^3^	991.5257	H^+^	990.5163	**2**	Figure S2
Hol. Ba.	594.3060 594.3064	31.1 31.4	2.21 × 10^3^ 2.21 × 10^5^	538.2796	NH_4_^+^	520.2461	**7** **9**	Figure S3
Hol. Ba.	761.3282 761.3293	31.7 34.2	4.74 × 10^5^ 4.36 × 10^4^	705.3031	Na^+^	682.3124	**10** **11**	Figure S4
Valerian	558.3575 558.3582	30.8 32.7	1.53 × 10^5^ 2.00 × 10^5^	502.3313	none	502.3313	**15**	Figure S5

## Data Availability

Please contact the corresponding author for raw research data.

## Supplementary Materials

The following supporting information can be downloaded at: https://www.mdpi.com/article/10.3390/molecules28155726/s1.
