# Supplementary material for: Tag and Snag: A New Platform for Bioactive Natural Product Screening from Mixtures
Source: Molecules. 2023 Jul 28;28(15):5726. doi: 10.3390/molecules28155726 (PMC10421080; doi:10.3390/molecules28155726)
Supplement: Supplementary file 1 [file molecules-28-05726-s001.zip › molecules-2514417-supplementary.pdf]

Supplementary Information

Table of contents

Extraction and Isolation of characterized compounds	Pages 2 - 3
Structural Characterization of tulsinols	Page 4
Structural Characterization of valeraninium alkaloids	Pages 5 - 6
Cell Affinity Assay Hit Ion Tables	Pages 7 - 9
MS1 spectra for selected isotopically labeled species	Pages 10 - 14
NMR data for isolated compounds	Pages 15 - 62
MS/MS characterization of proposed valeraninium structure	Page 63
Extracted Ion Chromatograms for valeraninium ions	Pages 64 - 68
Code for mass tag search	Pages 69 - 73

Extraction and Isolation:

For purpose of purification, five capsules of Ashwagandha (Herbal supplement from Sprouts Farmers Market) were extracted with 100 mL methanol, then centrifugated and evaporated to yield 58.8 mg of crude extract. The extract was loaded onto a C18 SPE column eluted with 30 % acetonitrile/70 % water, 50 % acetonitrile/50 % water, and 100 % acetonitrile to afford three fractions. The 50 % acetonitrile/50 % water fraction (18.4 mg) was purified by HPLC on a C18 column (Phenomenex 250 × 10 mm) using acetonitrile/water (40:60) for 15 minutes, then from 40:60 to 47:53 for 21 minutes at a flow rate of 2.5 mL/min to yield the ashwagandhanolide 1 (1.30 mg), with retention time of 33.2 min.

Five capsules of Holy Basil (Herbal supplement from Sprouts Farmers Market) were extracted with 100 mL methanol, then centrifugated and evaporated to yield 179.6 mg of crude extract. The extract was loaded onto a C18 SPE column eluted with 50 % acetonitrile/50 % water, 70 % acetonitrile/30 % water, and 100 % acetonitrile to afford three fractions. The 100 % acetonitrile fraction (65.4 mg) was purified by HPLC on a C18 column (Phenomenex 250 × 10 mm) using acetonitrile/water (70:30) for 5 minutes, then from 70:30 to 100:0 for 30 minutes at a flow rate of 2.5 mL/min to yield the bis-eugenol 3 (0.48 mg), dehyfrodieugenol 4 (0.24 mg), tulsinol H 9 (1.22 mg), tulsinol B 5 (1.21 mg), tulsinol D 7 (0.94 mg), tulsinol C 6 (1.88 mg), tulsinol F 8 (1.10 mg), tulsinol I 10 (0.87 mg), and tulsinol J 11 (1.68 mg), with retention time of 14.5 min, 16.5 min, 21.4 min, 21.8 min, 22.3 min, 23.2 min, 24.8 min, 26.8 min, and 29.2 min.

Twenty-five capsules of Valerian (Herbal supplement from Sprouts Farmers Market) were extracted with 500 mL methanol, then centrifugated and evaporated to yield 445.2 mg of crude extract. The extract was loaded onto a C18 SPE column eluted with 100 % acetonitrile, 100 % acetonitrile with 0.1% TFA, and 100 % methanol with 0.1% TFA to afford three fractions. The 100 % acetonitrile with 0.1% TFA fraction (30.9 mg) was purified by HPLC on a C18 column (Phenomenex 250 × 10 mm) using acetonitrile/water (10:90) with 0.1% TFA for 5 minutes, then from 10:90 to 100:0 for 30 minutes at a flow rate of 2.5 mL/min to yield valeraninium A 12 (1.07 mg), valeraninium C 14 (0.86 mg), and valeraninium B 13 (0.93 mg), with retention time of 33.6 min, 33.8 min, and 34.2 min.

Ashwagandhanolide **1**: white, amorphous solid; HRESIMS m/z 975.5297 [M+H]+ (calcd. for C56H79O12S+, 975.5287); 1H NMR (900 MHz, DMSO-d6) and 13C NMR (from 1H-13C HSQC and 1H-13C HMBC, DMSO-d6), see Figure S6.

Bis-eugenol **3**: white, amorphous solid; HRESIMS m/z 327.1601 [M+H]+ (calcd. for C20H23O4+, 327.1591); 1H NMR (900 MHz, DMSO-d6) and 13C NMR (from 1H-13C HSQC and 1H-13C HMBC, DMSO-d6), see Figure S7.

Dehydrodieugenol **4**: white, amorphous solid; HRESIMS m/z 327.1614 [M+H]+ (calcd. for C20H23O4+, 327.1591); 1H NMR (900 MHz, DMSO-d6) and 13C NMR (225 MHz, DMSO-d6), see Figure S8.

Tulsinol B **5**: yellow, amorphous solid; HRESIMS m/z 511.2091 [M+Na]+ (calcd. for C30H32O6Na+, 511.2091); 1H NMR (900 MHz, DMSO-d6) and 13C NMR (from 1H-13C HSQC and 1H-13C HMBC, DMSO-d6), see Figure S9.

Tulsinol C **6**: yellow, amorphous solid; HRESIMS m/z 511.2096 [M+Na]+ (calcd. for C30H32O6Na+, 511.2091); 1H NMR (900 MHz, DMSO-d6) and 13C NMR (from 1H-13C HSQC and 1H-13C HMBC, DMSO-d6), see Figure S10.

Tulsinol D **7**: yellow, amorphous solid; HRESIMS m/z 543.2365 [M+Na]+ (calcd. for C31H36O7Na+, 543.2353); 1H NMR (900 MHz, DMSO-d6) and 13C NMR (from 1H-13C HSQC and 1H-13C HMBC, DMSO-d6), see Figure S11.

Tulsinol F **8**: yellow, amorphous solid; HRESIMS m/z 511.2091 [M+Na]+ (calcd. for C30H32O6Na+, 511.2091); 1H NMR (900 MHz, DMSO-d6) and 13C NMR (from 1H-13C HSQC and 1H-13C HMBC, DMSO-d6), see Figure S12.

Tulsinol H **9**: yellow, amorphous solid; HRESIMS m/z 543.2359 [M+Na]+ (calcd. for C31H36O7Na+, 543.2353); 1H NMR (900 MHz, DMSO-d6) and 13C NMR (from 1H-13C HSQC and 1H-13C HMBC, DMSO-d6), see Figure S13.

Tulsinol I **10**: yellow, amorphous solid; HRESIMS m/z 705.3041 [M+Na]+ (calcd. for C41H46O9Na+, 705.3034); 1H NMR (900 MHz, DMSO-d6) and 13C NMR (from 1H-13C HSQC and 1H-13C HMBC, DMSO-d6), see Figure S14.

Tulsinol J **11**: yellow, amorphous solid; HRESIMS m/z 705.3047 [M+Na]+ (calcd. for C41H46O9Na+, 705.3034); 1H NMR (900 MHz, DMSO-d6) and 13C NMR (from 1H-13C HSQC and 1H-13C HMBC, DMSO-d6), see Figure S15.

Valeraninium A **12**: yellow, amorphous solid; HRESIMS m/z 470.3458 [M]+ (calcd. for C33H44NO+, 470.3417); 1H NMR (900 MHz, DMSO-d6) and 13C NMR (from 1H-13C HSQC and 1H-13C HMBC, DMSO-d6), see Figure S16.

Valeraninium B **13**: yellow, amorphous solid; HRESIMS m/z 470.3446 [M]+ (calcd. for C33H44NO+, 470.3417); 1H NMR (900 MHz, DMSO-d6) and 13C NMR (from 1H-13C HSQC and 1H-13C HMBC z, DMSO-d6), see Figure S17.

Valeraninium C **14**: yellow, amorphous solid; HRESIMS m/z 486.3408 [M]+ (calcd. for C33H44NO2+, 486.3367); 1H NMR (900 MHz, DMSO-d6) and 13C NMR (from 1H-13C HSQC and 1H-13C HMBC, DMSO-d6), see Figure S18.

Structural Characterization of Novel Tulsinols:

Structural elucidation of tulsinol H **9**. Tulsinol H **9** had the molecular formula $C_{31}H_{36}O_7$ based on a sodium adduct ion at m/z 543.2359 [M+Na]+ in its positive ion HRESIMS spectrum. Investigating the ^1H NMR, HSQC, and HMBC spectroscopic data of tulsinol H **9** (Table S8) in comparison with those of the known tulsinol D **7** (Table S7) indicated that tulsinol H **9** shared the same core structure of tulsinol D **7** with the different positions of one methoxy group. The known compound **7** had one methoxy group (δ_H 3.69, δ_C 55.3) at C-3 (δ_C 149.2), while the methoxy group (δ_H 3.61, δ_C 55.1) of tulsinol H **9** was assigned to C-4 (δ_C 146.5). Furthermore, the structure of tulsinol H **9** was confirmed by its HMBC spectrum.

Structural elucidation of tulsinol I **10**. Tulsinol I **10** was isolated as a yellow amorphous solid. Its positive ion HRESIMS data showed a peak for a sodium adduct ion at m/z 705.3041, corresponding to a molecular formula of $C_{41}H_{46}O_9$. Analysis of its ^1H NMR, HSQC, and HMBC spectroscopic data (Table S9) in comparison with those of the known tulsinol D **7** (Table S7) revealed that tulsinol I **10** had a similar core structure to tulsinol D **7** but added one more 4-allyl 2-methoxyphenoxy moiety. Moreover, the planar structure of tulsinol I **10** was confirmed through analysis of its HMBC spectrum. Depended on the vicinal coupling constant $^3J_{7,8}$ of 3.3 Hz, the relative configurations of C-7 and C-8 were confirmed as 7R*, 8R*.

Structural elucidation of tulsinol J **11**. Tulsinol J **11** was also isolated as an amorphous yellow solid and had the molecular formula $C_{41}H_{46}O_9$ based on its positive mode HRESIMS, which showed an intense peak at m/z 705.3047 [M+Na]+, corresponding to $C_{41}H_{46}O_9Na^+$(calcd. 705.3034). Comparison of the ^1H NMR, HSQC, and HMBC spectroscopic data (Table S10) with those of tulsinol I **10** (Table S9) indicated that both compounds shared the same skeleton, but their substituents were at different positions. The 4-allyl-2-methoxyphenoxy moiety was connected to C-8 (δ_C 79.3), which was supported by ^3J-HMBC correlation between H-8 (δ_H 4.36) and C-4’ (δ_C 145.9). Moreover, the relative configurations of C-7 and C-8 were assigned as 7R*, 8R* based on the vicinal coupling constant $^3J_{7,8}$ of 4.0 Hz.

Structural Characterization of Valeraninium Alkaloids:

Structural elucidation of valeraninium A **12**. Valeraninium A **12** had the molecular formula $C_{33}H_{44}NO^+$ based on a molecular ion at m/z 470.3446 $[M]^+$ in its positive ion HRESIMS spectrum. Comparison of its ^{1}H NMR, HSQC, and HMBC spectroscopic data (Table S12) with those of valeraninium A **12** implied that valeraninium A **12** contains the similar moiety of 6,7-dihydro-2-(p-hydroxyphenethyl)-7-methyl-5H-2-pyridinium as valeraninium C **14**. The remaining substructure of isoledene was confirmed by comparison with the NMR data in the reference [30]. Furthermore, the isoledene moiety was attached to the pyridinium through the carbon carbon bond between C-10’ and C-3’’, which was confirmed by COSY correlation between H-10’ and H-3’’. Meanwhile, the configuration of valeraninium A **12** was indicated by the known reference [30].

Structural elucidation of valeraninium B **13**. Valeraninium B **13** had the molecular formula $C_{33}H_{44}NO^+$ based on a molecular ion at m/z 470.3446 $[M]^+$ in its positive ion HRESIMS spectrum. Comparison of its ^{1}H NMR, HSQC, and HMBC spectroscopic data (Table S13) with those of valeraninium B **13** implied that valeraninium B **13** contained the similar moiety of 6,7-dihydro-2-(p-hydroxyphenethyl)-7-methyl-5H-2-pyridinium as valeraninium B **13**. The remaining substructure of 1,2-didehydro-aromadendrane was confirmed by comparison with the NMR data in the reference [35]. Moreover, this sesquiterpene part was connected to the pyridinium through the carbon carbon bond between C-10’ and C-4’’, as indicated by ^3J-HMBC correlation between H-15’’ and C-10’. Besides that, the configuration of valeraninium B **13** was also indicated by the known reference [30].

Structural elucidation of valeraninium C **14**. Valeraninium C **14** was isolated as an amorphous yellow solid. Its positive ion HRESIMS revealed a peak for a molecular ion at m/z 486.3408, corresponding to the molecular formula $C_{33}H_{44}NO_2^+$. Its ^{1}H NMR and HSQC spectra displayed signals for one individual AA’BB’ *p*-aromatic spin systems [□$_H$ 6.97, H-2 and H-6 (2H); □$_H$ 6.66, H-3 and H-5 of (2H)], two aromatic methines (□$_H$ 8.71, H-1’;□□$_H$ 8.69, H-8’), one vinyl methine (□$_H$ 5.75, H-11’’), three heteromethylenes (□$_H$ 4.76, H-8a;□□$_H$ 4.68, H-8b; □$_H$ 4.51, H-10’a;□□$_H$ 4.49, H-10’b;□□$_H$ 3.91, H-13’’a; □$_H$ 3.90, H-13’’b), four alkyl methines (□$_H$ 3.38, H-3’; □$_H$ 3.46, H-5’’; □$_H$ 1.93, H-8’’; □$_H$ 2.87, H-9’’), seven alkyl methylenes (□$_H$ 3.13, H-7a; □$_H$ 3.06, H-7b; □$_H$ 2.42, H-4’a; □$_H$ 1.68, H-4’b; □$_H$ 3.10, H-5’a; □$_H$ 2.98, H-5’b;□□$_H$ 1.75, H-1’’a; □$_H$ 1.50, H-1’’b; □$_H$ 2.15, H-2’’a; □$_H$ 2.14, H-2’’b; □$_H$ 1.66, H-6’’a; □$_H$ 1.30, H-6’’b; □$_H$ 1.78, H-7’’a; □$_H$ 1.34, H-7’’b), and four methyl groups (□$_H$ 1.26, H-9’; □$_H$ 1.61, H-10’’; □$_H$ 1.67, H-14’’; □$_H$ 0.74, H-15’’). The presence of 6,7-dihydro-4-(hydroxymethyl)-2-(p-hydroxyphenethyl)-7-methyl-5H-2-pyridinium moiety was indicated by its COSY correlations between H-7a/b and H-8a/b, H-9’ and H-3’, H-3’ and H-4’a/b, and H-4’a/b and H-5’a/b, as well as ^2J-HMBC correlations between H-5’ and C-6’ (□$_C$ 162.0), and H-10’ and C-7’ (□$_C$ 134.7), and ^3J-HMBC correlations between H-2/H-6 and C-7 (□$_C$ 35.7), H-8 and C-1 (□$_C$ 126.1)/C-1’ (□$_C$ 137.9)/C-8’ (□$_C$ 140.5), H-1’/H-8’ and C-6’, H-10’a/b and C-6’/C-8’, and H-9’ and C-2’ (□$_C$ 148.1). On the other hand, the moiety of valerenol was confirmed by comparison of the ^{1}H NMR, HSQC, and HMBC spectroscopic data (Table S11) with those of valerenol in the reference [28]. These two substructures were linked to each other with an ether bond C-10’-O-C-

13’’, which was corroborated by ^{3}J-HMBC correlation between H-10’ and C-13’’ (□$_{C}$ 76.1). Meanwhile, the configuration of valeraninium C **14** was indicated by the known references [30].

Table S1. Isotopically labeled hit ions identified in the cell affinity assay for Ashwagandha.

	mass_Steg	time_Steg	intensity_Steg	mass_HeLa	time_HeLa	intensity_HeLa
**0**	652.3492	19.66206	1768936	652.3476	19.83059	12872.59
**1**	781.4639	20.50942	105624	781.4634	20.74134	4961.946
**2**	781.4639	20.95013	39707.86	781.4634	20.74134	4961.946
**3**	667.3947	21.67637	4330537	667.3958	21.85934	47920.88
**4**	1047.551	22.18301	38539.38	1047.551	22.30174	8091.228
**5**	719.3768	22.94482	1101358	719.3765	22.63986	56846.88
**6**	527.3011	22.97627	10700000	527.2996	23.03555	78131.98
**7**	549.2824	22.97627	1233252	549.2822	23.03555	16991.76
**8**	649.3853	23.07115	709554.4	649.3856	23.23817	21552.6
**9**	549.2836	23.29396	220873.5	549.2822	23.03555	16991.76
**10**	559.3268	23.32551	6030093	559.3268	23.37364	58510.95
**11**	581.3086	23.32551	684984.9	581.3084	23.37364	14887.89
**12**	563.278	23.48443	4881994	563.2764	23.54364	81457.78
**13**	565.2744	23.48443	1658176	565.274	23.54364	24317.56
**14**	1033.562	23.61093	30730.12	1033.565	23.60873	2754.895
**15**	1013.548	23.61093	25283.41	1013.547	23.60873	4468.908
**16**	1047.551	23.64273	390314.6	1047.552	23.6756	70817.55
**17**	527.3011	23.73836	1448952	527.3	23.77674	50228.77
**18**	685.3615	23.73836	1913070	685.3613	23.8801	21704.52
**19**	1153.641	23.77068	51365.49	1153.644	23.84562	9316.318
**20**	1063.53	24.41192	34723.93	1063.527	24.5041	2782.362
**21**	1031.555	24.54032	625744.9	1031.556	24.60252	43186.18
**22**	1053.538	24.54032	29662.48	1053.539	24.60252	3172.822
**23**	1169.635	24.57239	59764.59	1169.636	24.7318	11639.91
**24**	707.3719	24.57239	325753.6	707.373	24.7318	18445.99
**25**	667.3514	24.73318	362305.5	667.3503	24.89717	34841.2
**26**	669.3488	24.73318	115556.3	669.3476	24.89717	8880.996
**27**	1136.632	24.86214	131131	1136.634	24.99473	15113.41
**28**	1135.631	24.86214	195174.4	1135.632	24.99473	21145.58
**29**	1063.529	24.95871	55264.45	1063.527	24.5041	2782.362
**30**	1135.631	26.48778	231284.4	1135.631	26.59629	9480.388
**31**	835.3799	28.93519	11176.36	835.3794	28.99175	1637.621
**32**	476.3006	31.25368	46812.27	476.3004	31.31233	27883.27
**33**	313.1797	35.38006	36516.83	313.1799	35.48933	47804.79
**34**	477.3658	37.36497	145840.8	477.3665	37.51053	145067.7
**35**	370.332	37.3965	756757.2	370.3318	37.51053	655820
**36**	442.3529	38.26588	233434.3	442.3529	38.42299	93545.83

Table S2. Isotopically labeled hit ions identified in the cell affinity assay for Holy Basil.

	mass_Steg	time_Steg	intensity_Steg	mass_HeLa	time_HeLa	intensity_HeLa
**0**	664.4118	16.41067	2637320	664.41	16.13405	6292.598
**1**	748.4329	16.5035	707379.8	748.4328	16.26597	3670.326
**2**	708.4367	16.6268	2188149	708.438	16.42904	6956.542
**3**	752.4635	16.84263	1773888	752.4631	16.65721	7572.937
**4**	840.5163	17.24512	679950.8	840.5159	17.05177	4031.255
**5**	711.3909	24.80156	49447.23	711.3926	24.66713	37396.26
**6**	868.5674	27.21914	22939.79	868.5642	26.89776	7106.303
**7**	405.1672	28.05692	522025.1	405.1672	28.34314	44260.27
**8**	405.1671	28.42785	1774463	405.1672	28.34314	44260.27
**9**	790.4581	28.58188	35482.08	790.459	28.98364	30919.89
**10**	553.3376	28.8305	118663.5	553.3369	28.72529	51128.27
**11**	588.3747	28.8305	278421.4	588.374	28.72529	111411
**12**	593.3296	28.86111	115355.5	593.3302	28.72529	80983.37
**13**	585.2454	29.04816	153460.4	585.246	29.43668	16767.89
**14**	585.2453	29.51505	480817.3	585.246	29.43668	16767.89
**15**	567.2357	30.01226	572086.1	567.2348	29.76057	239328.3
**16**	777.3255	30.13693	325142.7	777.3239	30.04631	81780.46
**17**	757.2863	30.2603	220710	757.285	30.20346	6363.707
**18**	747.3148	30.29114	713676.5	747.3135	30.20346	243066.5
**19**	505.222	30.29114	442394.6	505.2221	30.52247	123685.1
**20**	505.2219	30.56937	862137.9	505.2221	30.52247	123685.1
**21**	757.2862	30.60021	117968.2	757.285	30.20346	6363.707
**22**	562.2803	30.7548	1906960	562.2792	30.6521	116355.9
**23**	545.2531	31.00343	569113.7	545.2527	31.30854	535022.4
**24**	562.2801	31.12732	2903501	562.2792	30.6521	116355.9
**25**	594.306	31.12732	5367631	594.3061	31.08172	221081.6
**26**	615.235	31.12732	236011.7	615.235	31.08172	10418.76
**27**	599.2614	31.12732	1153144	599.2614	31.05165	114499.2
**28**	545.2533	31.37585	1609474	545.2527	31.30854	535022.4
**29**	594.3064	31.43797	3359552	594.3061	31.08172	221081.6
**30**	599.2617	31.43797	698172	599.2614	31.05165	114499.2
**31**	615.2354	31.43797	121947.6	615.235	31.08172	10418.76
**32**	756.3748	31.65566	760321.5	756.3748	31.60037	135980.9
**33**	761.3282	31.65566	1154550	761.3301	31.60037	473560.8
**34**	742.359	31.84092	359754.9	742.3585	31.82619	67700.93
**35**	747.3141	31.84092	175662.8	747.3135	31.79348	101208.6
**36**	756.3752	31.90291	482232.5	756.3748	31.60037	135980.9
**37**	655.5049	32.6385	224692	655.5037	32.61163	60826.84
**38**	909.3823	33.06863	93990.39	909.3832	32.98201	27662.24
**39**	798.3962	33.12967	194805.7	798.3951	33.04601	19083.61
**40**	747.3126	33.19106	129573.3	747.3137	33.32288	21987.29
**41**	729.3043	33.28322	143172.8	729.3032	33.75446	75947.05

**42**	756.3757	33.46803	1865294	756.3746	33.81723	139812.4
**43**	724.3483	33.80859	2460335	724.3473	33.72407	184166.8
**44**	729.3042	33.80859	326694.9	729.3032	33.75446	75947.05
**45**	766.3696	33.80859	181368.3	766.371	33.72407	42478.8
**46**	756.374	33.90082	2441360	756.3746	33.81723	139812.4
**47**	886.4163	33.90082	202183.2	886.4179	33.87853	21726.37
**48**	888.4311	34.05456	224249.6	888.4326	34.11202	37060.14
**49**	918.4418	34.05456	326413.5	918.4424	34.22781	48448.41
**50**	923.3985	34.02365	313948.3	923.3994	34.25694	52038.9
**51**	729.3041	34.14677	475114.1	729.3032	33.75446	75947.05
**52**	724.3482	34.17706	1944441	724.3473	33.72407	184166.8
**53**	756.3739	34.14677	3549659	756.3746	33.81723	139812.4
**54**	381.1699	34.17706	396929.6	381.1694	34.16985	33209.35
**55**	761.3292	34.17706	991237.1	761.3301	33.7854	43609.21
**56**	904.4274	34.33146	135221.8	904.4281	34.34531	23926.73
**57**	1092.523	35.28671	9198.485	1092.521	35.2705	2450.019
**58**	923.3981	35.31811	360528.6	923.3992	35.30251	24573.71
**59**	961.47	35.28671	52297.05	961.468	35.2705	4051.876
**60**	891.3716	35.34833	103989.3	891.3703	35.33465	13213.16
**61**	918.4414	35.31811	1112615	918.4423	35.46376	37030.11
**62**	562.36	35.37918	251908	562.3602	35.46376	56492.19
**63**	886.416	35.37918	300373.1	886.4172	35.39888	11066.55
**64**	477.3661	36.05042	186170.1	477.366	36.05522	132126.1
**65**	562.3608	36.35551	190805.5	562.3594	36.44579	64941.23
**66**	643.382	37.36914	56886.22	643.3811	37.15567	36951.35
**67**	639.5098	37.8163	547945.9	639.5101	37.82639	98733.86

Table S3. Isotopically labeled hit ions identified in the cell affinity assay for Valerian.

	**mass_Steg**	**time_Steg**	**intensity_Steg**	**mass_HeLa**	**time_HeLa**	**intensity_HeLa**
**0**	725.4727	23.58311	21034.22	725.4736	23.81556	17922.45
**1**	540.2812	26.17398	107309.7	540.2813	26.32517	25176.92
**2**	269.102	30.13768	55479.3	269.1017	30.19334	22137.1
**3**	558.3578	30.76082	342171.2	558.3572	30.98514	153231.2
**4**	591.3421	31.50151	175609.4	591.3421	31.68011	28394.84
**5**	558.3582	32.73596	309293.2	558.3578	32.98517	199898.5
**6**	435.2016	33.13449	72881.73	435.2014	33.11101	17086.89
**7**	659.4248	33.89368	29560.52	659.4239	33.90987	59104.14
**8**	586.3604	33.98594	165326.1	586.3604	34.05508	71406.2
**9**	313.1797	35.37004	431131.4	313.1798	35.44148	257984.8
**10**	435.2008	35.84836	101966.6	435.2012	35.94113	27767.9
**11**	477.3661	37.29203	930886.1	477.3665	37.38883	588239.3
**12**	370.3322	37.32233	1901220	370.3317	37.38883	662637.7

1. **Mass Spectrometry Data for Crude Natural Products Extracts**

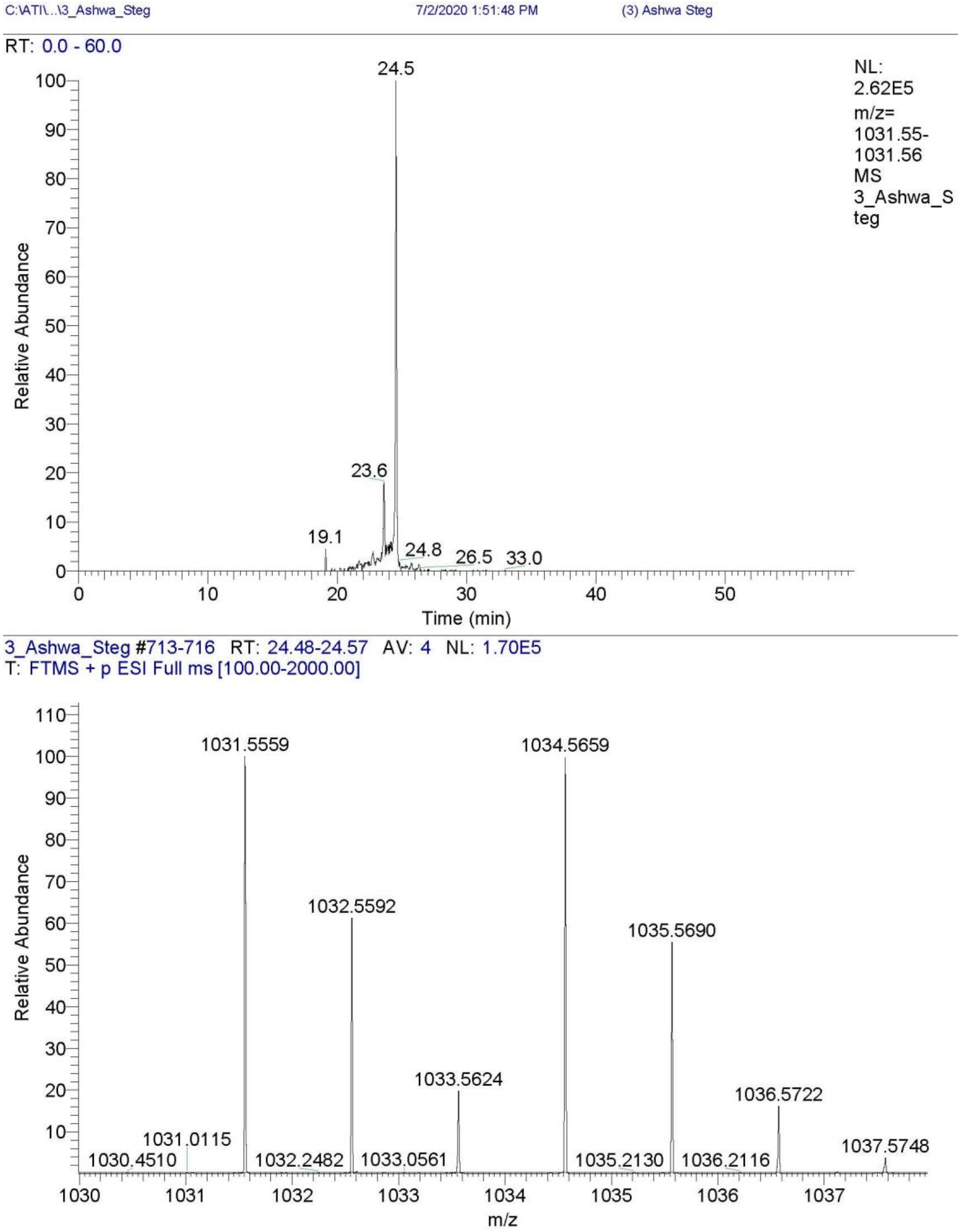

Figure S1: EIC and mass spectra showing tagged compound from Ashwagandha m/z 1031.5559

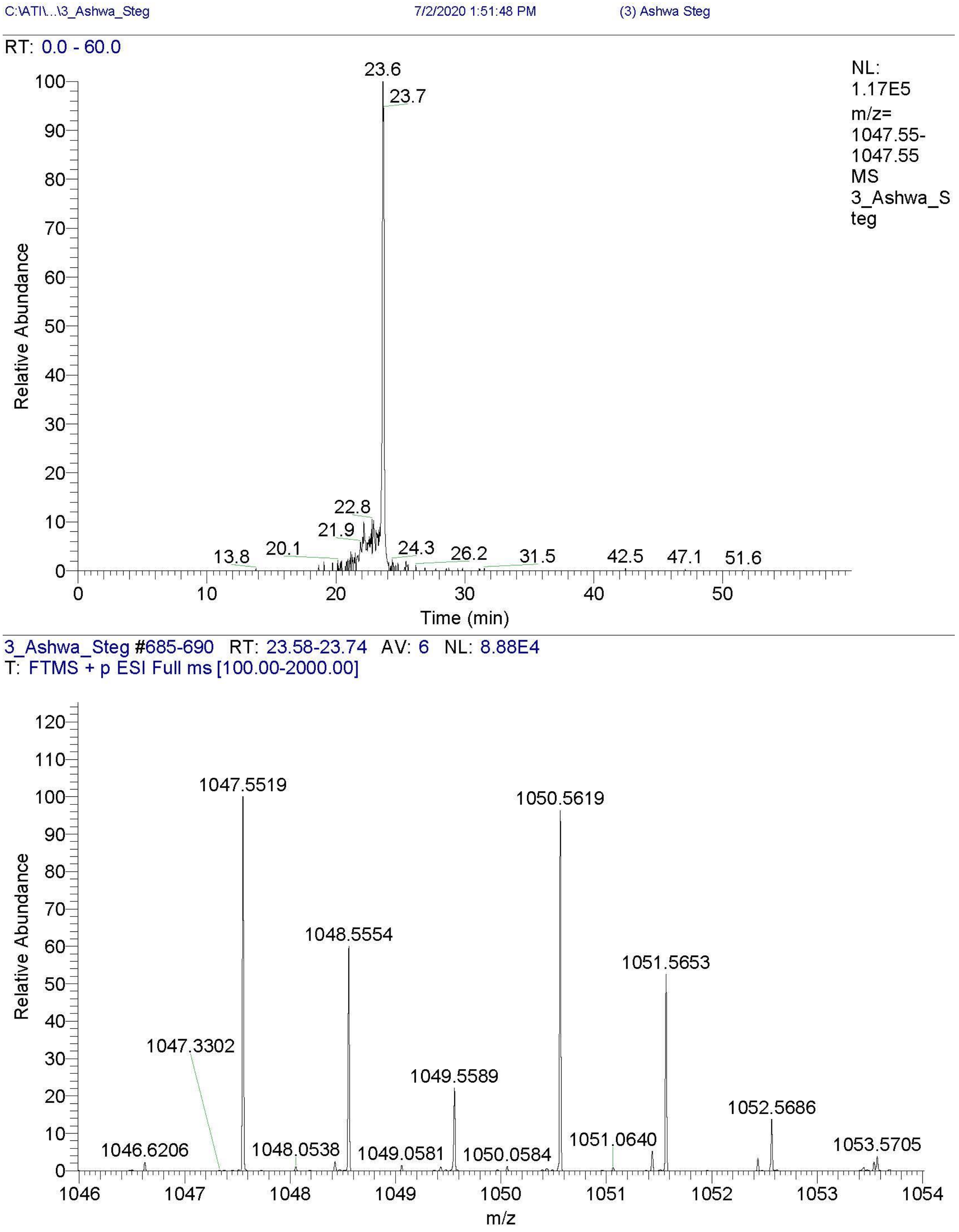

Figure S2: EIC and mass spectra showing tagged compound from Ashwagandha m/z 1047.5519

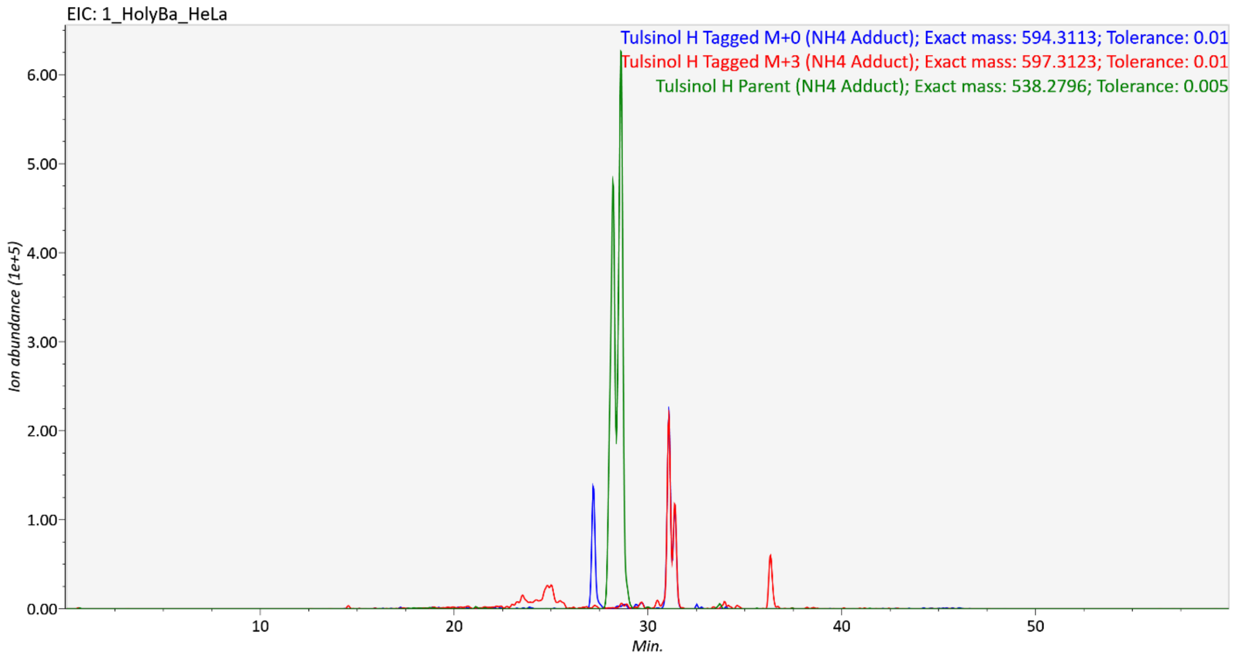

Figure S3: EIC showing isomeric tagged compounds from Holy Basil m/z 594.3113 which originate from two isomeric parent ions with m/z 538.2796.

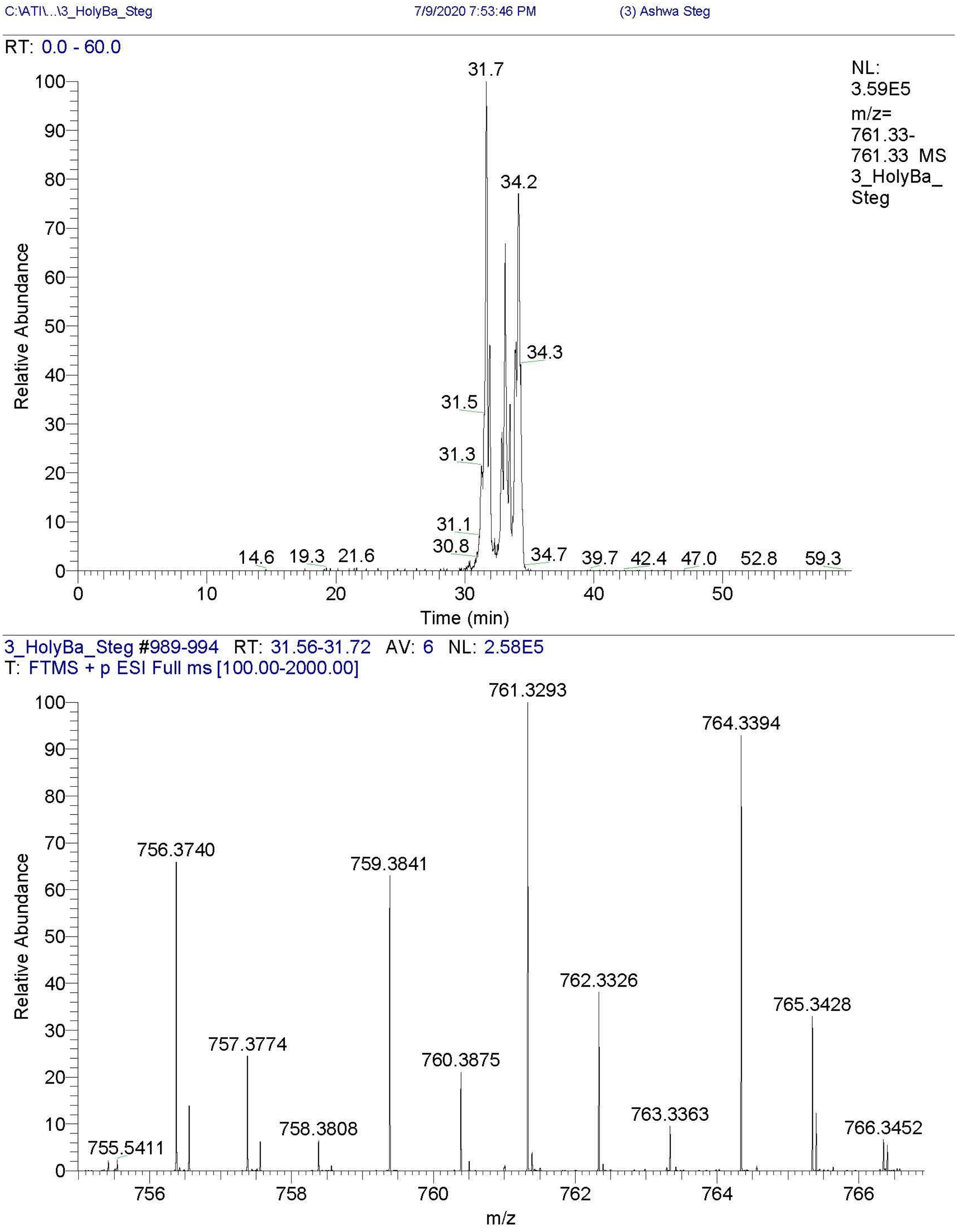

Figure S4: EIC and mass spectra showing tagged compound from Holy Basil m/z 761.3293

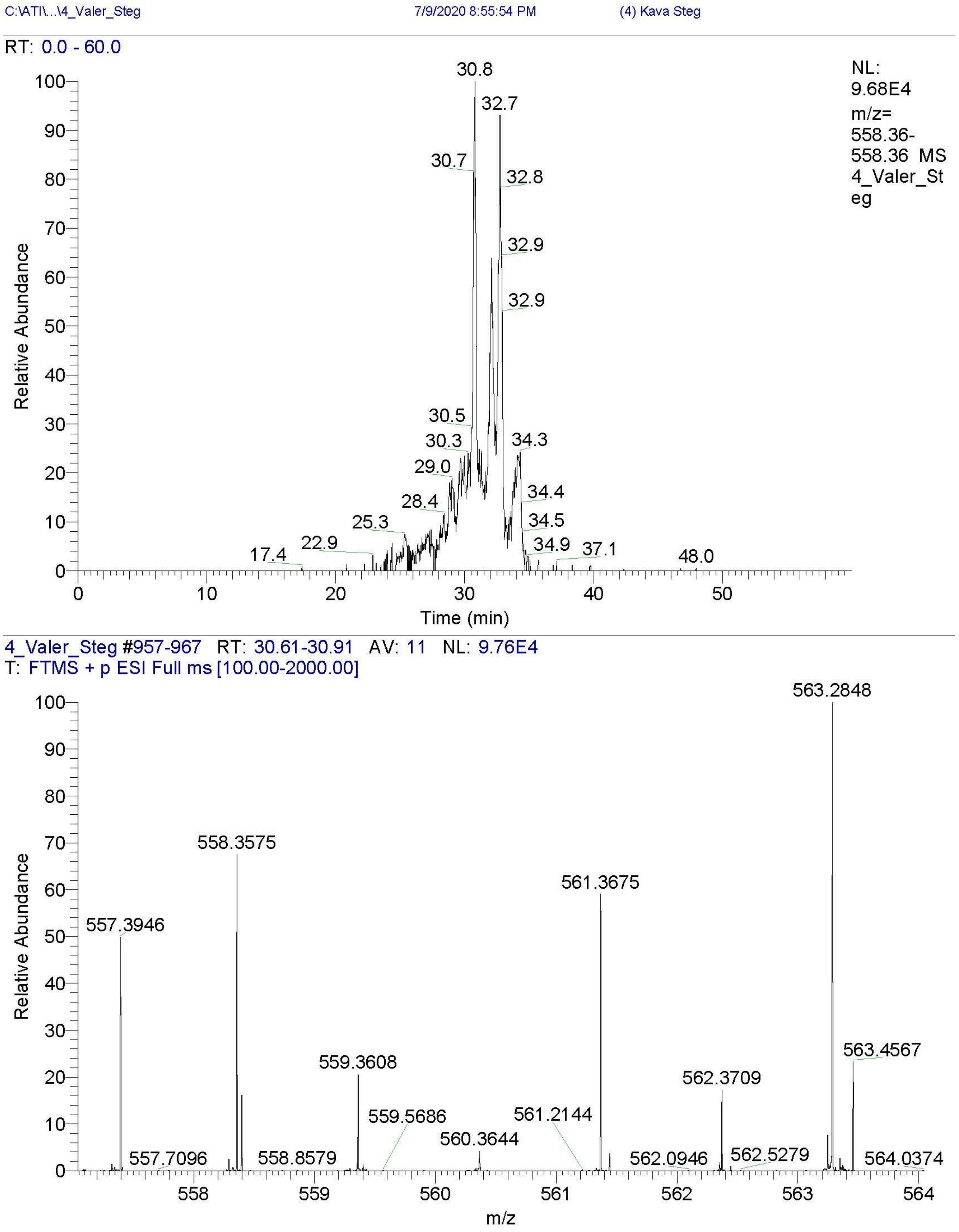

Figure S5: EIC and mass spectra showing tagged compound from Valerian m/z 558.3575

2. NMR data for identified compounds.

NMR Data of Ashwagandhanolide in DMSO-d_6					
Position	δ_H (J in Hz)	δ_C (C type)	Position	δ_H (J in Hz)	δ_C (C type)
1, 1'		201.5, C	15, 15'	1.54 m	23.5, CH_2
2, 2'	5.81 dd 10.3, 2.4	125.4, CH		1.12 m	
3, 3'	6.48 dd 10.3, 2.4	147.7, CH	16, 16'	1.73 m	26.3, CH_2
4, 4'	4.76 br	64.7, CH		1.31 m	
5, 5'		79.1, C	17, 17'	1.12 m	50.9, CH
6, 6'	2.73 dd 12.8, 4.4	51.3, CH	18, 18'	0.67 s	11.5, CH_3
7, 7'	2.03 m	36.6, CH_2	19, 19'	1.13 s	9.4, CH_3
	1.40 q 12.8		20, 20'	1.78 m	38.1, CH
8, 8'	1.57 m	34.7, CH	21, 21'	0.88 d 6.6	12.8, CH_3
9, 9'	1.30 m	45.0, CH	22, 22'	4.29 dt 13.5, 3.5	77.6, CH
10, 10'		56.3, C	23, 23'	2.38 m	28.7, CH_2
11, 11'	1.23 m	22.7, CH_2		2.08 dd 18.5, 3.5	
	0.77 m		24, 24'		154.7, C
12, 12'	1.04 m	38.5, CH_2	25, 25'		125.4, C
	1.82 m		26, 26'		165.4, C
13, 13'		42.6, C	27, 27'	4.15 d 11.4	54.2, CH_2
14, 14'	1.11 m	54.5, CH		4.10 d 11.4	
			28, 28'	2.00 s	19.7, CH_3

Figure S6 A: NMR Data for Ashwagandhanolide in DMSO-d_6.

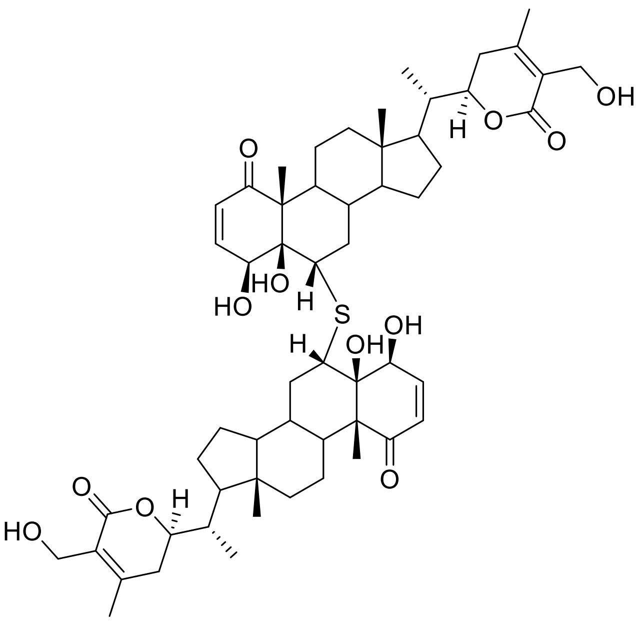

Ashwagandhanolide (^{1}H NMR, DMSO-d_6 at 900 MHz)

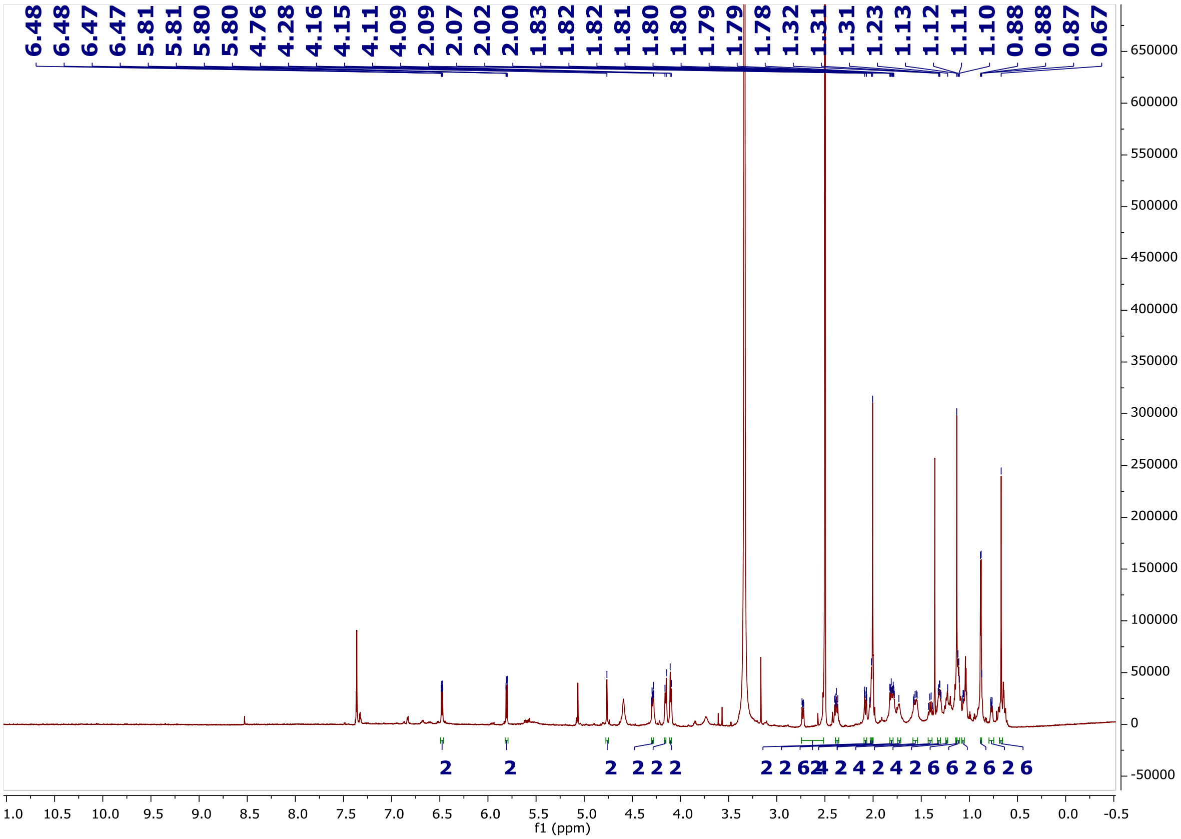

Figure S6 B: ^{1}H NMR, DMSO-d_6 at 900 MHz data for Ashwagandhanolide.

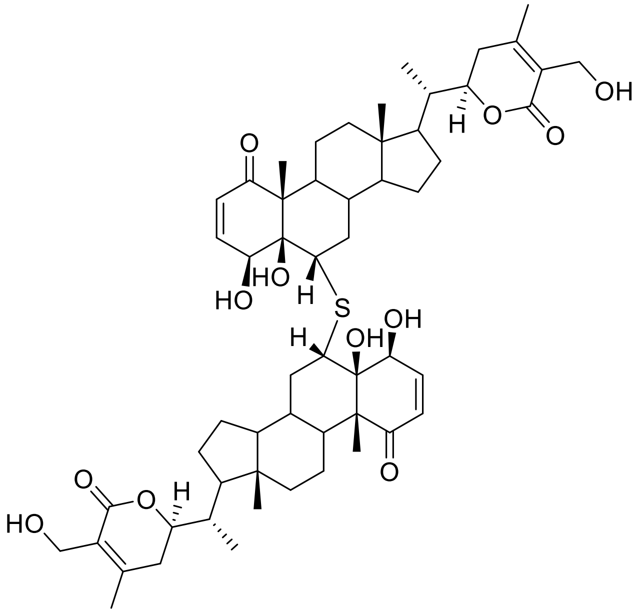

Ashwagandhanolide (^{1}H-^{13}C HSQC, DMSO-d_6 at 900 MHz)

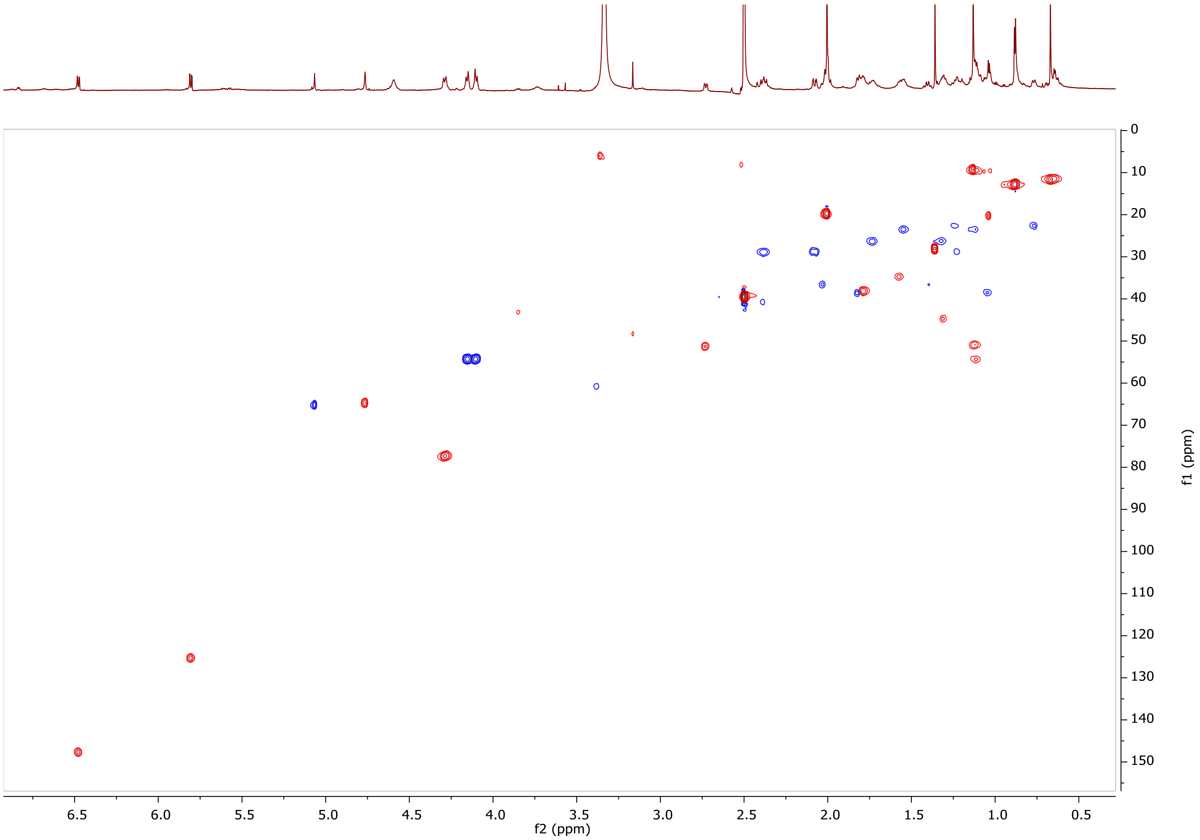

Figure S6 C: ^{1}H-^{13}C HSQC, DMSO-d_6 at 900 MHz data for Ashwagandhanolide.

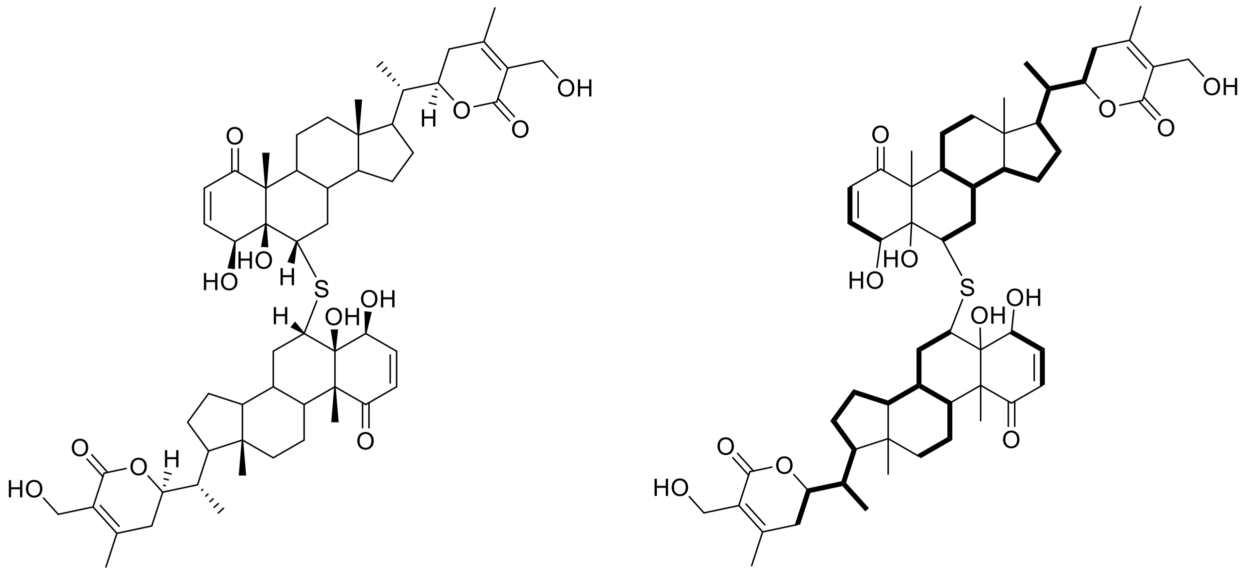

Ashwagandhanolide (^{1}H-^{1}H COSY, DMSO-d_6 at 900 MHz)

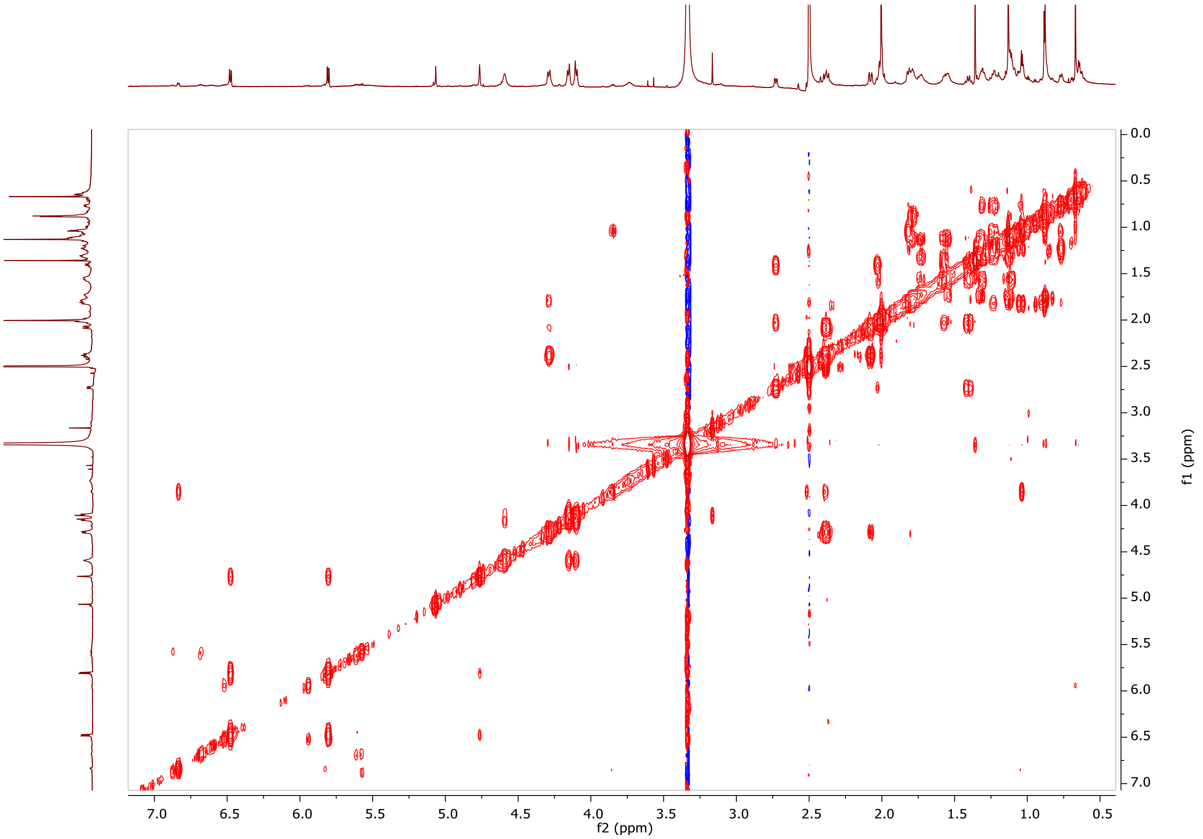

Figure S6 D: ^{1}H-^{13}C HSQC, DMSO-d_6 at 900 MHz data for Ashwagandhanolide.

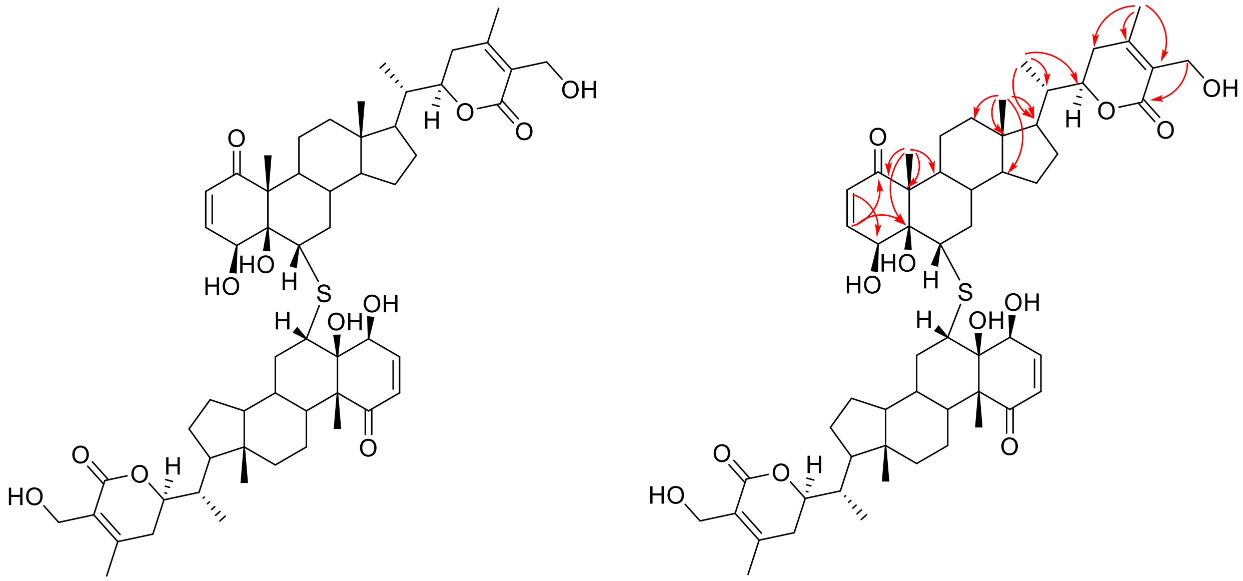

Ashwagandhanolide (^{1}H-^{13}C HMBC, DMSO-d_6 at 900 MHz)

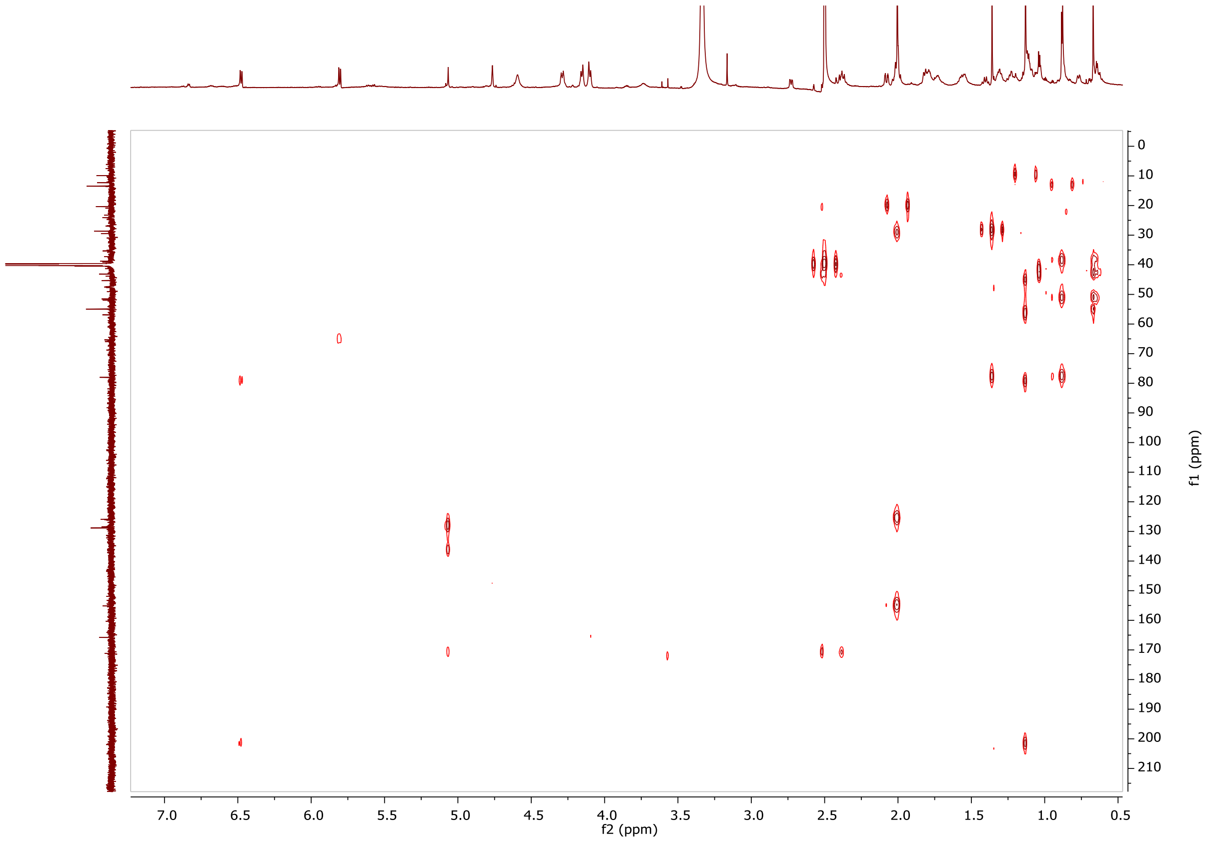

Figure S6 E: ^{1}H-^{13}C HMBC, DMSO-d_6 at 900 MHz data for Ashwagandhanolide.

The NMR Data of bis-eugenol in DMSO-d_6		
Position	d_H (J in Hz)	d_C (C type)
1, 1'		n.d.
2, 2'	6.63 d 2.2	110.5, CH
3, 3'		148.4, C
4, 4'		n.d.
5, 5'		126.7, C
6, 6'	6.55 d 2.2	122.4, CH
7, 7'	3.26 d 6.8	39.1, CH_2
8, 8'	5.95 ddt 16.7, 9.7, 6.8	138.2, CH
9, 9'	5.08 dd 16.7, 2.2	114.5, CH_2
	5.00 dd 9.7, 2.2	
OMe	3.74 s	55.4, CH_3

Figure S7 A: NMR Data for bis-eugenol in DMSO-d_6.

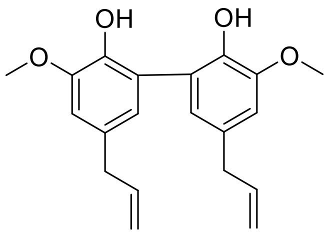

bis-eugenol (^{1}H NMR, DMSO-d_6 at 900 MHz)

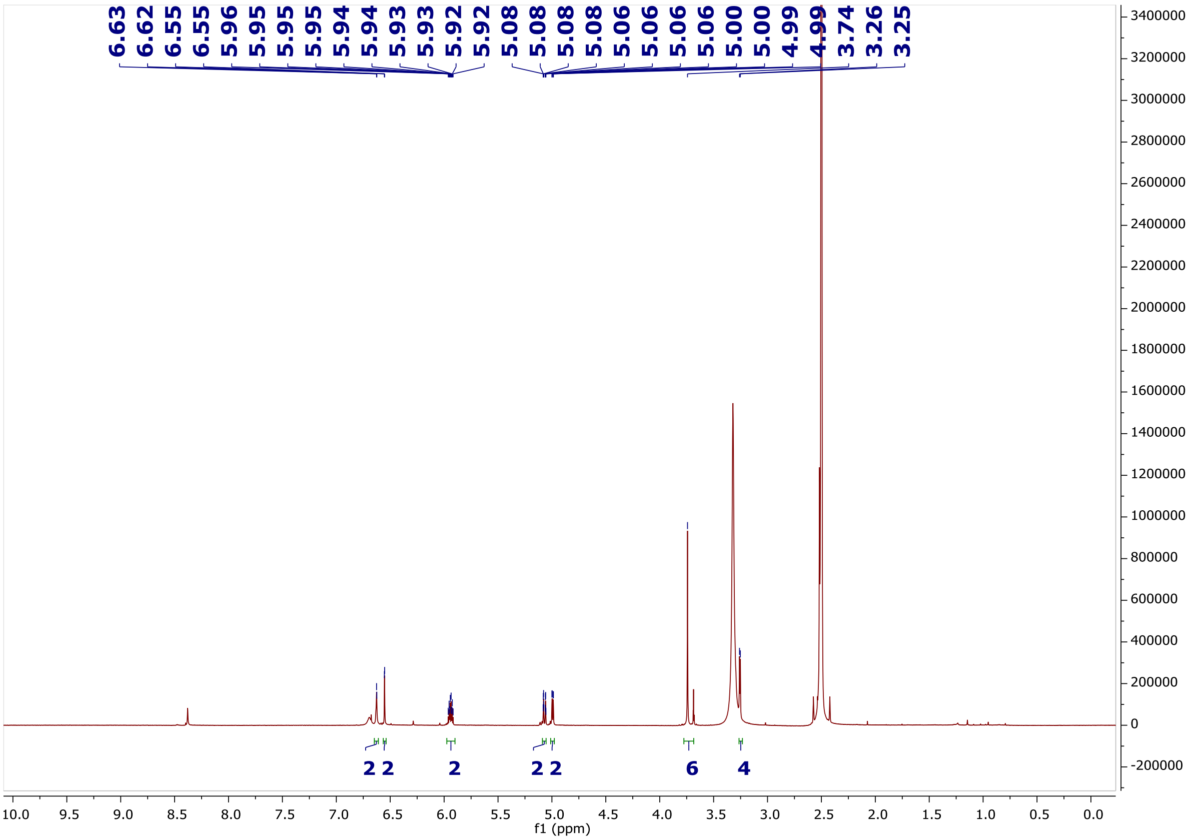

Figure S7 B: ^{1}H NMR, DMSO-d_6 at 900 MHz data for bis-eugenol.

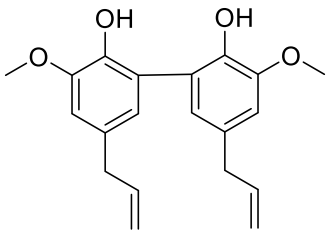

bis-eugenol (^{1}H-^{13}C HSQC, DMSO-d_6 at 900 MHz)

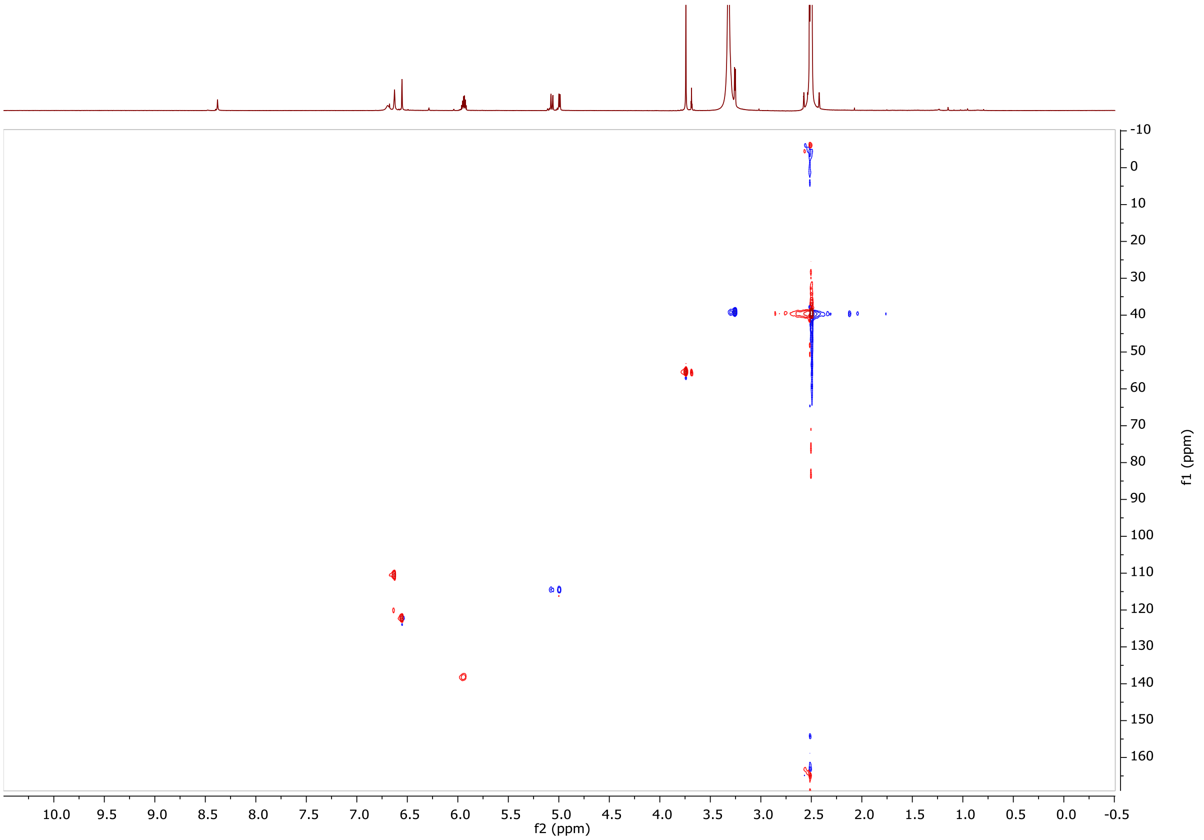

Figure S7 C: ^{1}H-^{13}C HSQC, DMSO-d_6 at 900 MHz data for bis-eugenol.

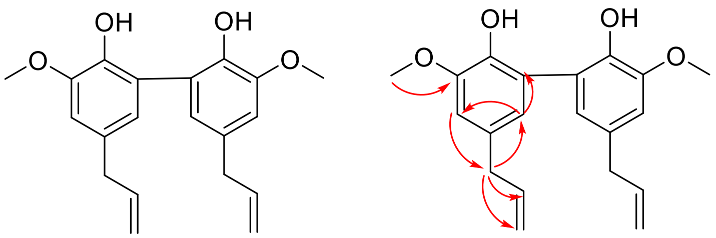

bis-eugenol (^{1}H-^{13}C HMBC, DMSO-d_6 at 900 MHz)

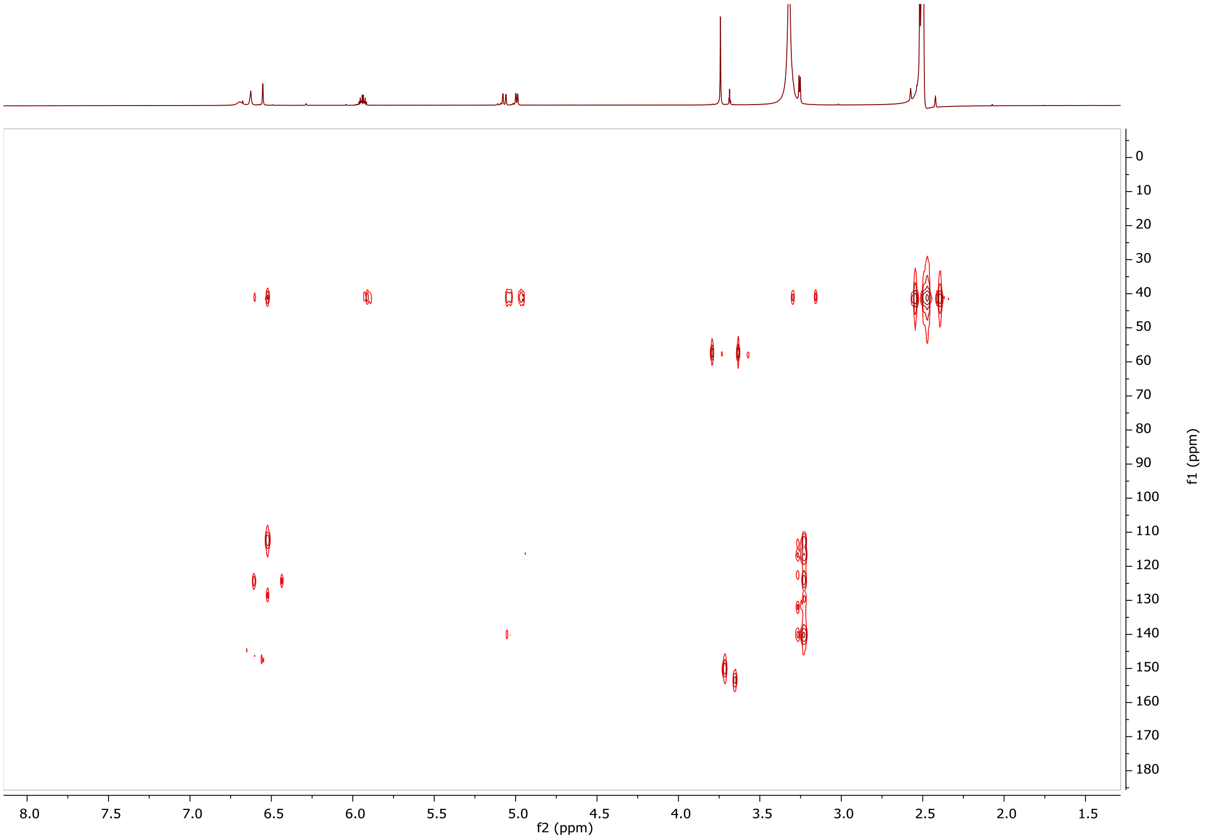

Figure S7 D: ^{1}H-^{13}C HMBC, DMSO-d_6 at 900 MHz data for bis-eugenol.

NMR Data of dehydrodieugenol in DMSO-d_6					
Position	δ_H (J in Hz)	δ_C (C type)	Position	δ_H (J in Hz)	δ_C (C type)
1		130.1, C	1’		n.d.
2	6.55 d 2.0	108.1, CH	2’	6.91 d 2.0	113.6, CH
3		148.4, C	3’		149.3, C
4		135.7, C	4’		144.3, C
5		145.1, C	5’	6.63 d 8.1	118.5, CH
6	6.14 d 2.0	111.4, CH	6’	6.67 d 8.1, 2.0	120.8, CH
7	3.18 d 6.8	39.5, CH_2	7’	3.32 d 6.8	39.4, CH_2
8	5.86 ddd 16.8, 10.0, 6.8	138.1, CH	8’	5.96 ddd 16.8, 10.0, 6.8	138.0, CH
9	5.00 dd 16.8, 2.0	115.7, CH_2	9’	5.09 dd 16.8, 1.8	115.7, CH_2
	4.97 dd 10.0, 2.0			5.05 dd 10.0, 1.8	
3-OMe	3.78 s	56.3, CH_3	3’-OMe	3.76 s	56.0, CH_3
4-OH	8.51 s				

Figure S8 A: NMR Data for dehydrodieugenol in DMSO-d_6.

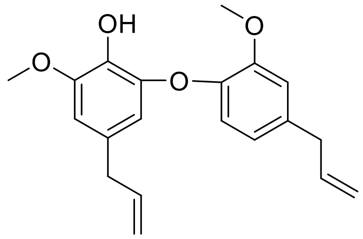

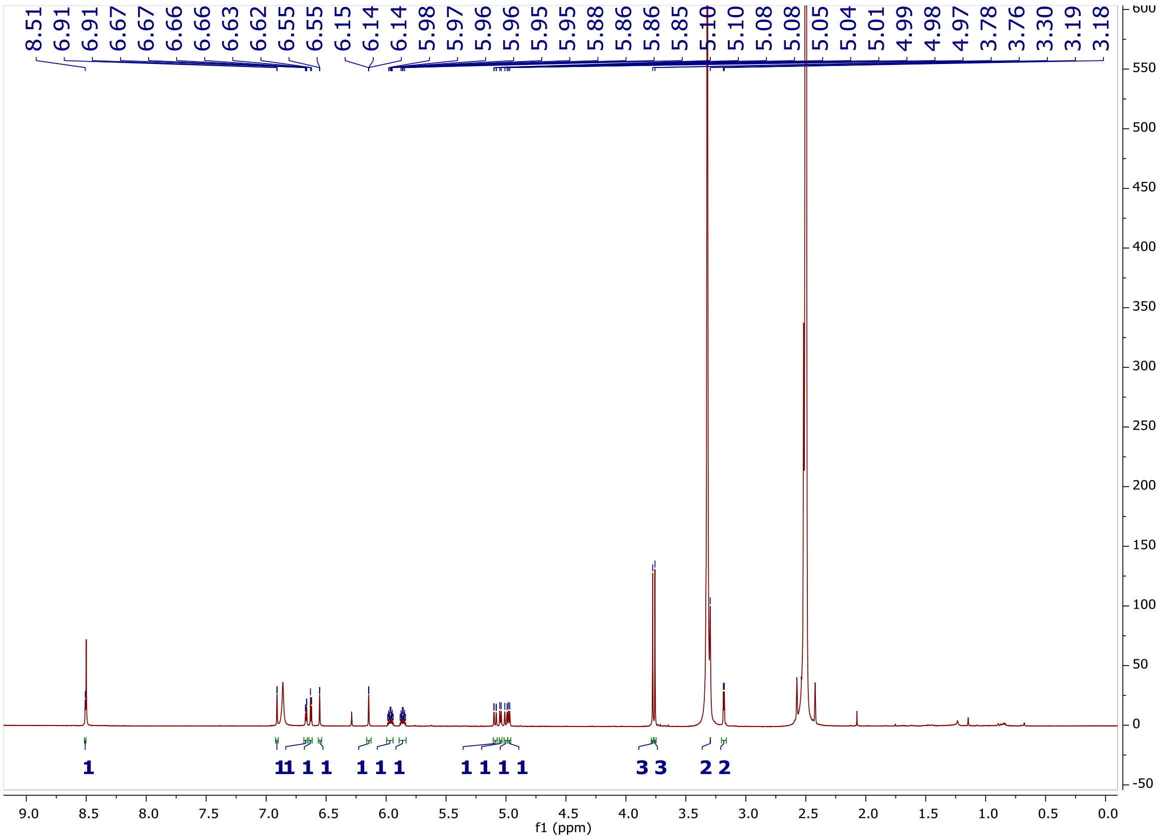

Figure S8 B: ^{1}H NMR, DMSO-d_6 at 900 MHz data for dehydrodieugenol.

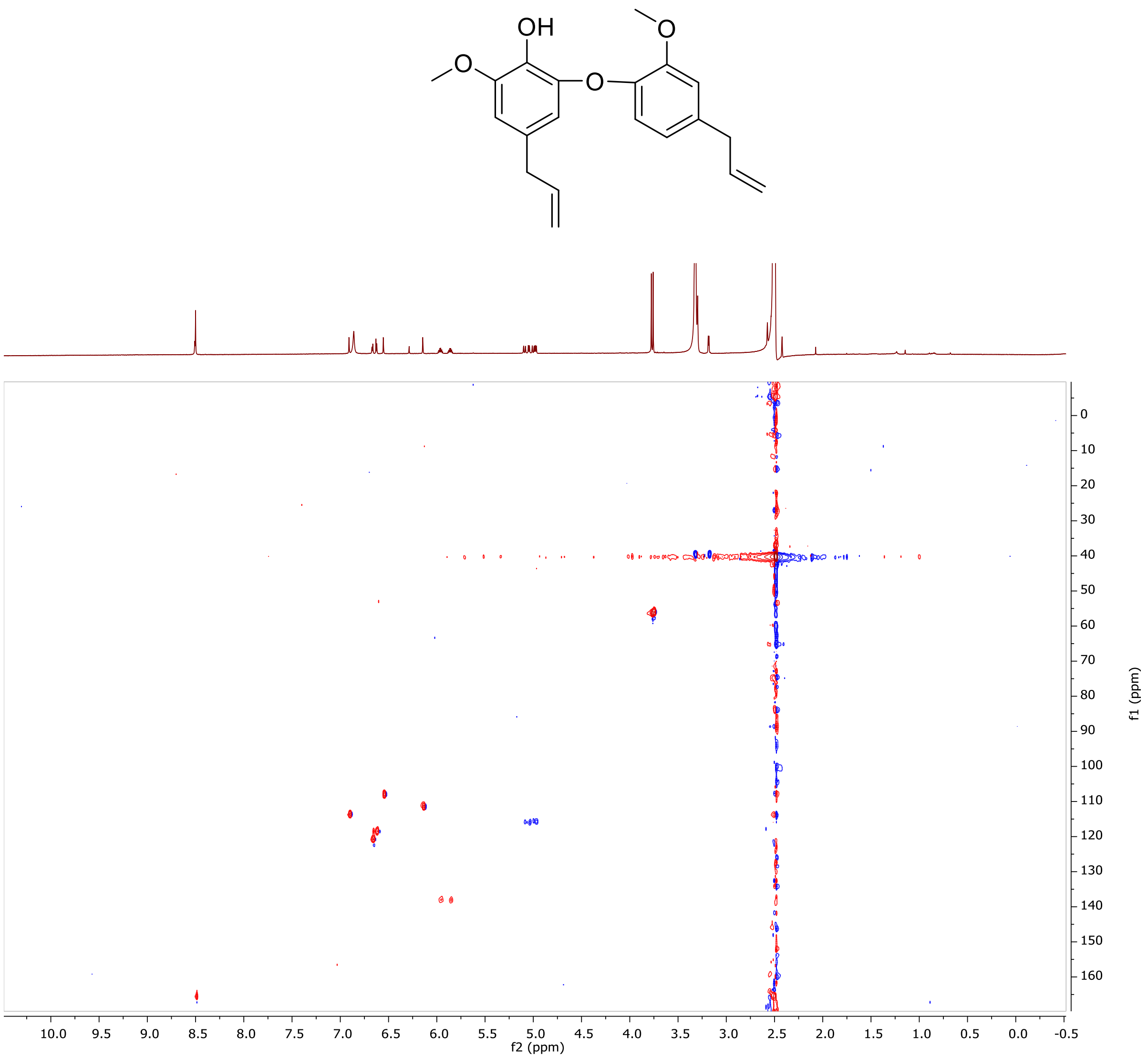

Figure S8 C: ^{1}H-^{13}C HSQC, DMSO-d_6 at 900 MHz data for dehydrodieugenol.

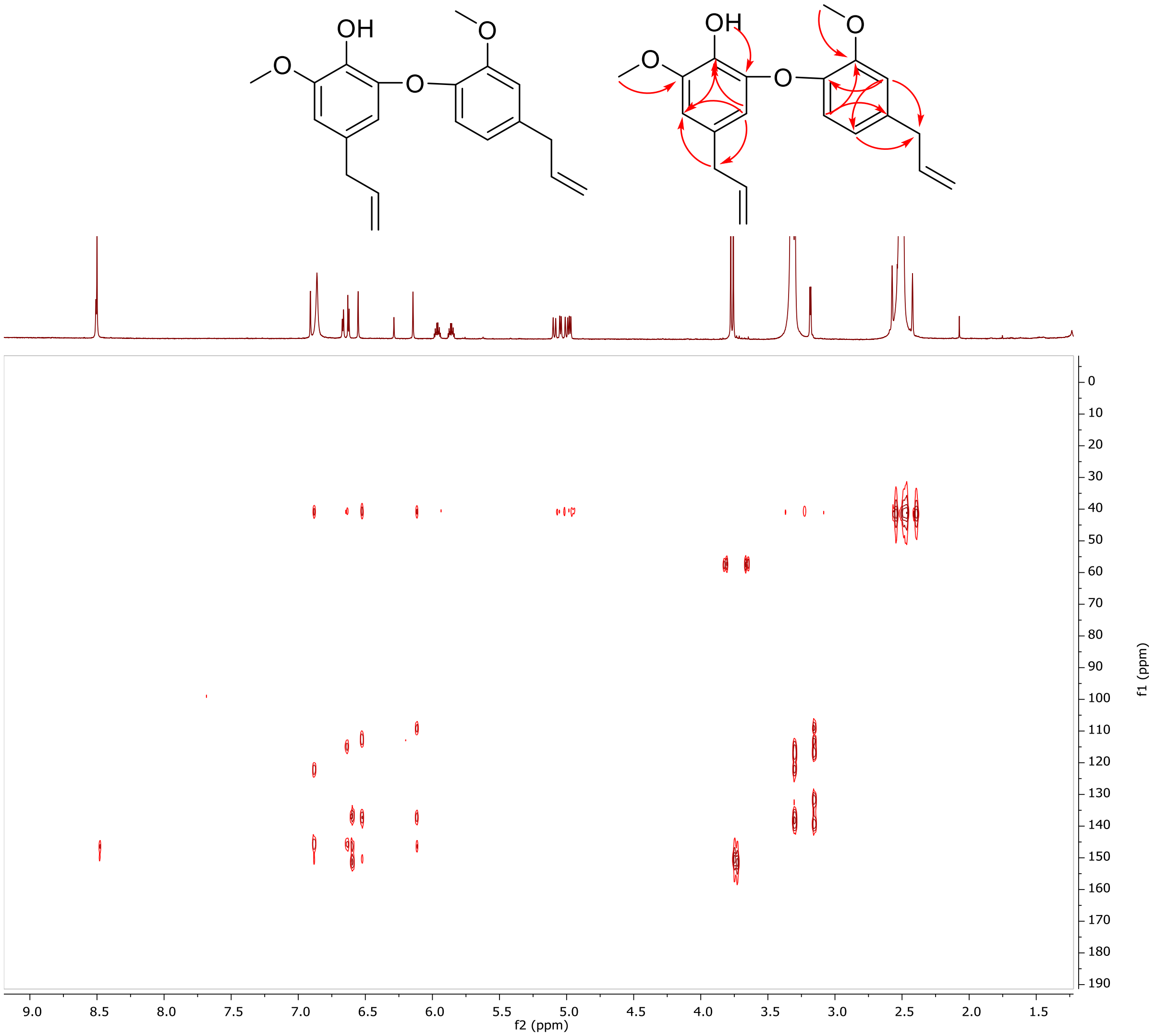

Figure S9 D: ^{1}H-^{13}C HMBC, DMSO-d_6 at 900 MHz data for dehydrodieugenol.

The NMR Data of tulsinol B in DMSO-d_6					
Position	δ_H (J in Hz)	δ_C (C type)	Position	δ_H (J in Hz)	δ_C (C type)
1		131.9, C	1”		133.9, C
2	6.65 d 1.8	110.8, CH	2”	6.76 d 1.8	113.1, CH
3		147.2, C	3”		150.0, C
4		141.2, C	4”		144.4, C
5		120.1, C	5”	6.83 d 8.4	117.8, CH
6	6.46 d 1.8	121.7, CH	6”	6.61 dd 8.4, 1.8	120.8, CH
7	3.24 d 7.0	39.2, CH_2	7”	3.26 d 7.0	39.2, CH_2
8	5.92 ddt 17.2, 11.0, 7.0	138.3, CH	8”	5.92 ddt 17.2, 11.0, 7.0	138.3, CH
9	5.05 m	115.9, CH_2	9”	5.05 m	115.9, CH_2
	5.01 m			5.01 m	
1’		129.5, C	3-OMe	3.68 s	55.9, CH_3
2’	6.94 d 1.8	111.4, CH	3’-OMe	3.72 s	55.9, CH_3
3’		147.2, C	3”-OMe	3.66 s	55.9, CH_3
4’		146.3, C			
5’	6.71 d 7.9	115.6, CH			
6’	6.77 dd 7.9, 1.8	119.9, CH			
7’	5.08 d 6.4	78.8, CH			
8’	4.85 q 6.4	73.8, CH			
9’	2.94 dd 16.7, 6.4	29.4, CH_2			
	2.85 dd 16.7, 6.4				

Figure S9 A: NMR Data for tulsinol B in DMSO-d_6.

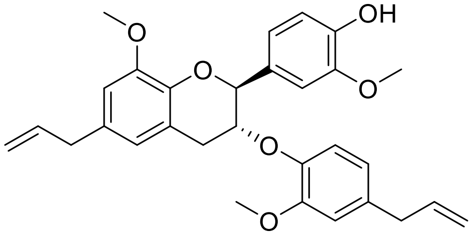

tulsinol B (^{1}H NMR, DMSO-d_6 at 900 MHz)

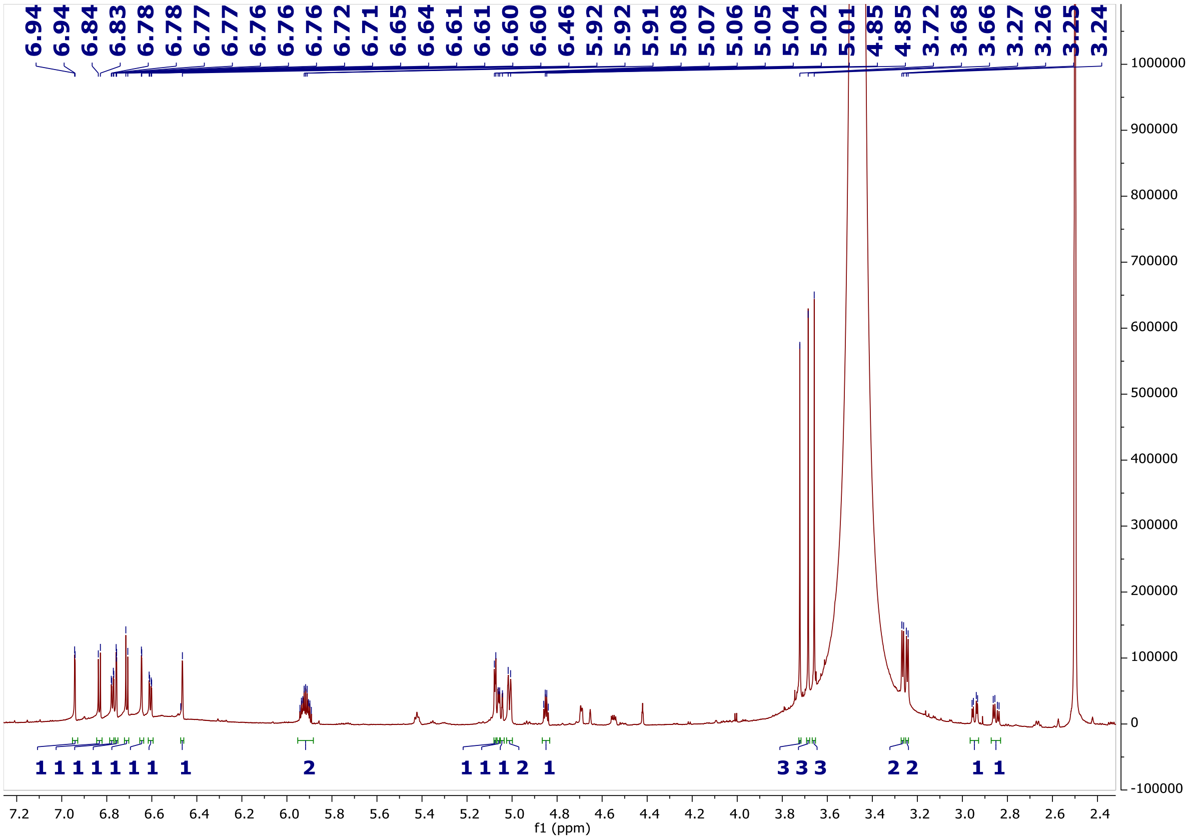

Figure S9 B: ^{1}H NMR, DMSO-d_6 at 900 MHz data for tulsinol B.

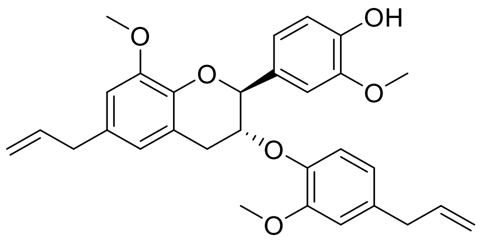

tulsinol B (^{1}H-^{13}C HSQC, DMSO-d_6 at 900 MHz)

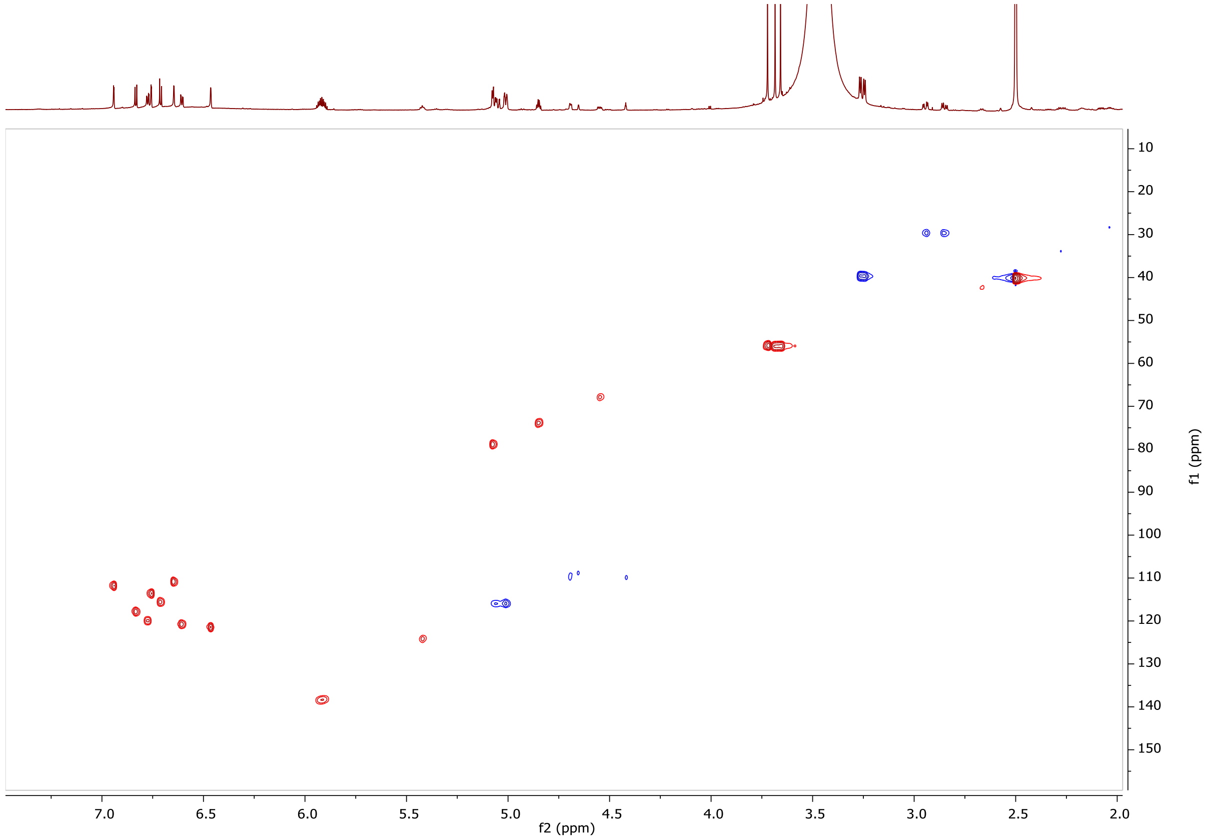

Figure S9 C: ^{1}H-^{13}C HSQC, DMSO-d_6 at 900 MHz data for tulsinol B.

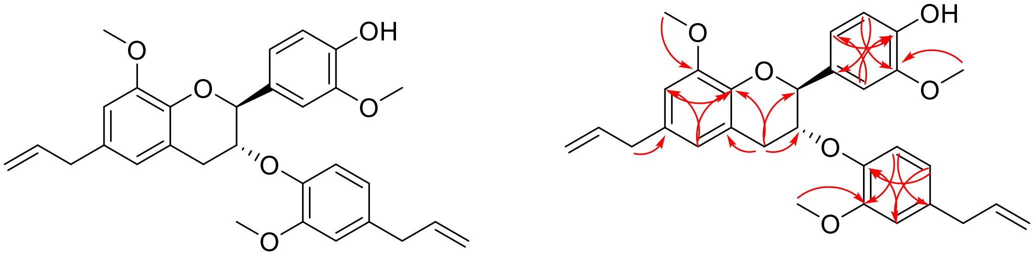

tulsinol B (^{1}H-^{13}C HMBC, DMSO-d_6 at 900 MHz)

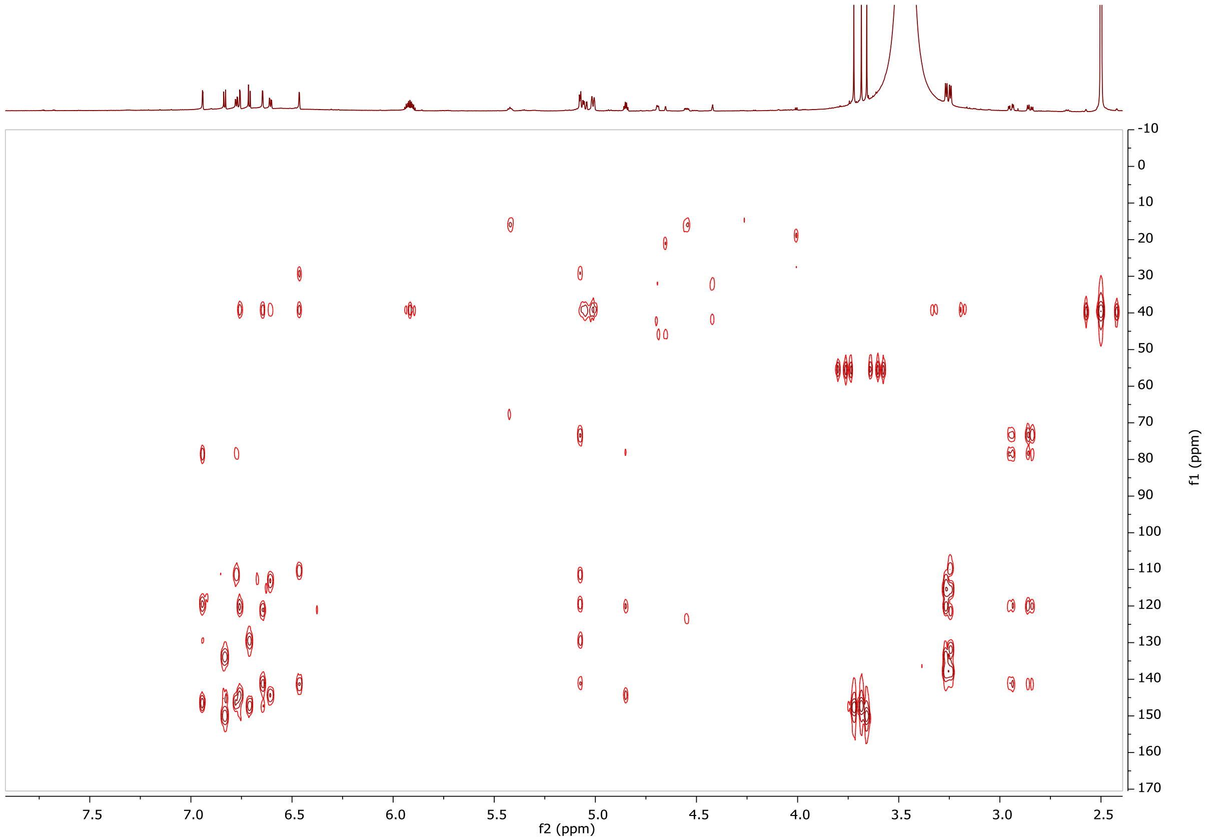

Figure S9 D: ^{1}H-^{13}C HMBC, DMSO-d_6 at 900 MHz data for tulsinol B.

The NMR Data of tulsinol C in DMSO-d_6					
Position	δ_H (J in Hz)	δ_C (C type)	Position	δ_H (J in Hz)	δ_C (C type)
1		132.7, C	1”		133.2, C
2	6.72 d 1.8	112.6, CH	2”	6.64 d 2.6	112.7, CH
3		143.5, C	3”		149.2, C
4		145.8, C	4”		146.2, C
5		n.d.	5”	6.93 d 8.4	113.9, CH
6	6.84 d 1.8	116.9, CH	6”	6.67 dd 8.4, 2.6	120.1, CH
7	3.29 d 6.2	39.1, CH_2	7”	3.30 d 6.2	39.1, CH_2
8	5.93 ddt 17.3, 10.6, 6.2	137.3, CH	8”	5.93 ddt 17.3, 10.6, 6.2	137.3, CH
9	5.07 m	115.3, CH_2	9”	5.07 m	115.3, CH_2
	5.02 m			5.02 m	
1’		131.9, C	3-OMe	3.77 s	56.0, CH_3
2’	6.94 d 1.9	109.9, CH	3’-OMe	3.73 s	56.0, CH_3
3’		147.2, C	3”-OMe	3.73 s	56.0, CH_3
4’		146.9, C			
5’	6.75 d 8.4	115.0, CH			
6’	6.80 dd 8.4, 1.9	118.3, CH			
7’	5.52 d 6.6	86.7, CH			
8’	3.77 m	50.4, CH			
9’	4.19 dd 9.7, 6.2	70.2, CH_2			

	4.14 dd 9.7, 7.0				

Figure S10 A: NMR Data for tulsinol C in DMSO-d_6.

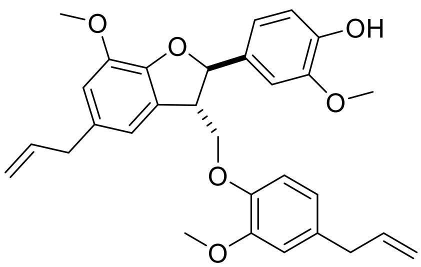

tulsinol C (^{1}H NMR, DMSO-d_6 at 900 MHz)

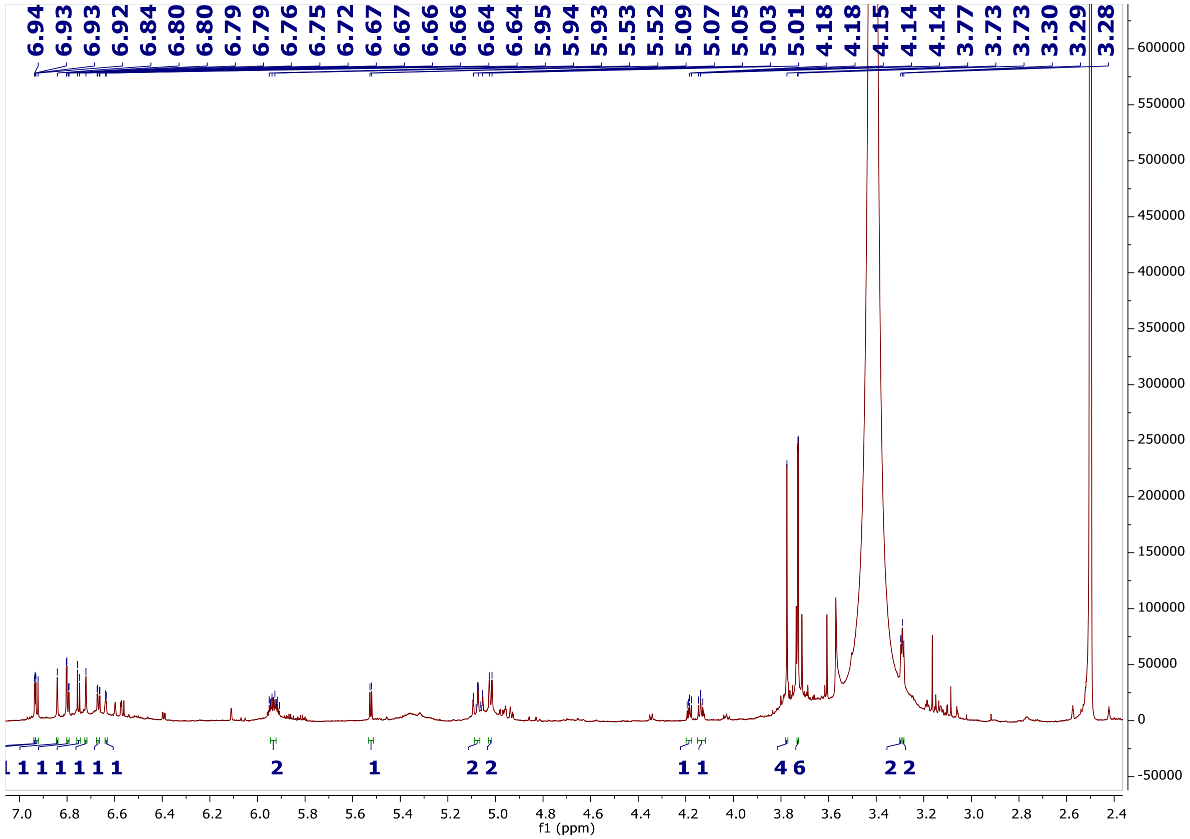

Figure S10 B: ^{1}H NMR, DMSO-d_6 at 900 MHz data for tulsinol C.

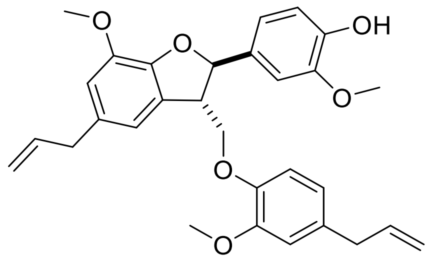

tulsinol C (^{1}H-^{13}C HSQC, DMSO-d_6 at 900 MHz)

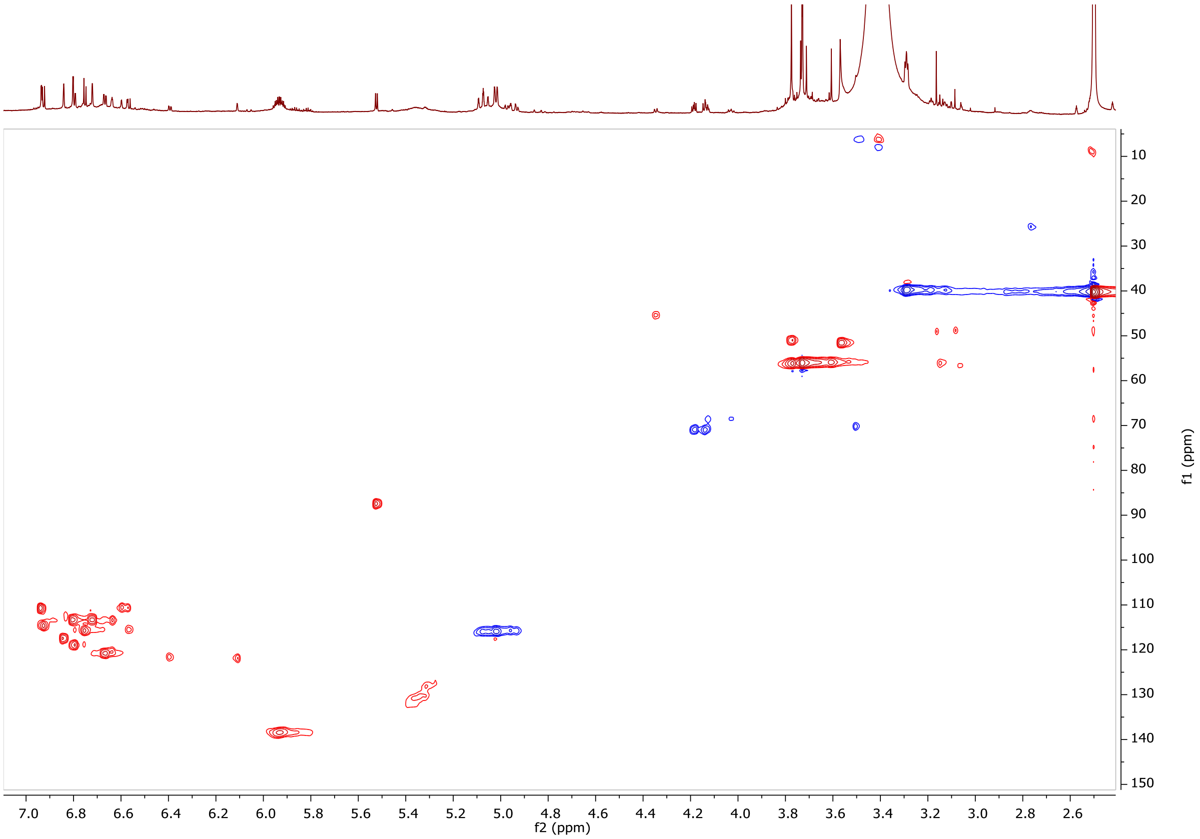

Figure S10 C: ^{1}H-^{13}C HSQC, DMSO-d_6 at 900 MHz data for tulsinol C.

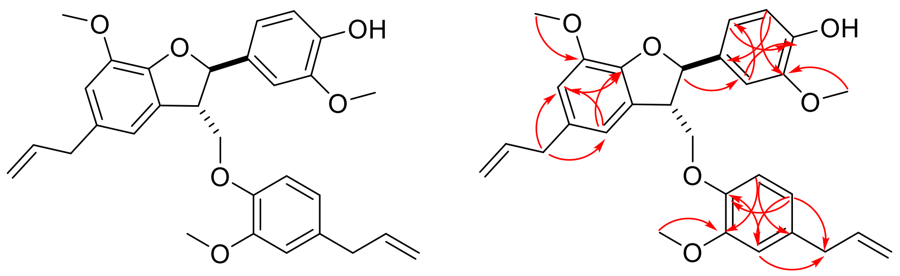

tulcinol C (^{1}H-^{13}C HMBC, DMSO-d_6 at 900 MHz)

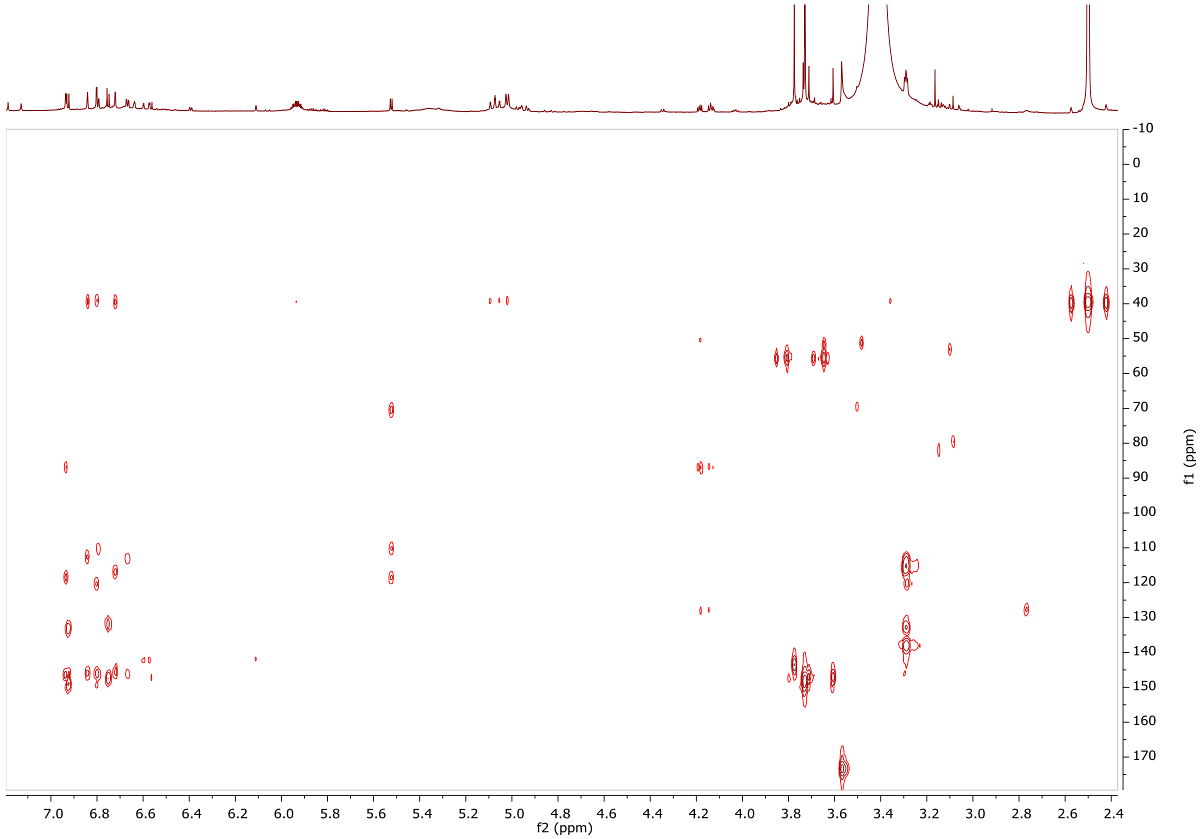

Figure S10 D: ^{1}H-^{13}C HMBC, DMSO-d_6 at 900 MHz data for tulsinol C.

The NMR Data of tulsinol D in DMSO-d_6					
Position	δ_H (J in Hz)	δ_C (C type)	Position	δ_H (J in Hz)	δ_C (C type)
1		128.5, C	1”		133.3, C
2	6.93 d 1.8	111.3, CH	2”	6.74 d 2.6	112.8, CH
3		149.2, C	3”		149.5, C
4		146.5, C	4”		146.1, C
5	6.77 m	113.8, CH	5”	6.93 d 8.0	116.6, CH
6	6.76 m	120.3, CH	6”	6.63 dd 8.0, 2.6	120.1, CH
7	4.47 d 5.3	81.6, CH	7”	3.26 d 7.4	38.8, CH_2
8	4.63 q 5.3	80.5, CH	8”	5.92 ddt 16.7, 10.1, 7.4	137.6, CH
9	4.10 dd 10.6, 5.3	67.8, CH_2	9”	5.05 m	115.3, CH_2
	4.08 dd 10.6, 5.3			5.01 m	
1’		128.4, C	3-OMe	3.69 s	55.3, CH_3
2’	6.77 m	112.9, CH	7-OMe	3.15 s	56.3, CH_3
3’		147.1, C	3’-OMe	3.70 s	55.3, CH_3
4’		145.8, C	3”-OMe	3.66 s	55.3, CH_3
5’	6.72 d 7.9	114.7, CH			
6’	6.62 dd 7.9, 2.6	120.1, CH			
7’	3.27 d 7.4	38.8, CH_2			
8’	5.92 ddt 16.7, 10.1, 7.4	137.6, CH			
9’	5.05 m	115.3, CH_2			
	5.01 m				

Figure S11 A: NMR Data for tulsinol D in DMSO-d_6.

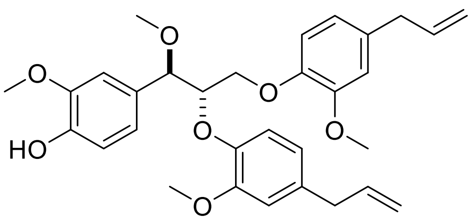

tulsinol D (^{1}H NMR, DMSO-d_6 at 900 MHz)

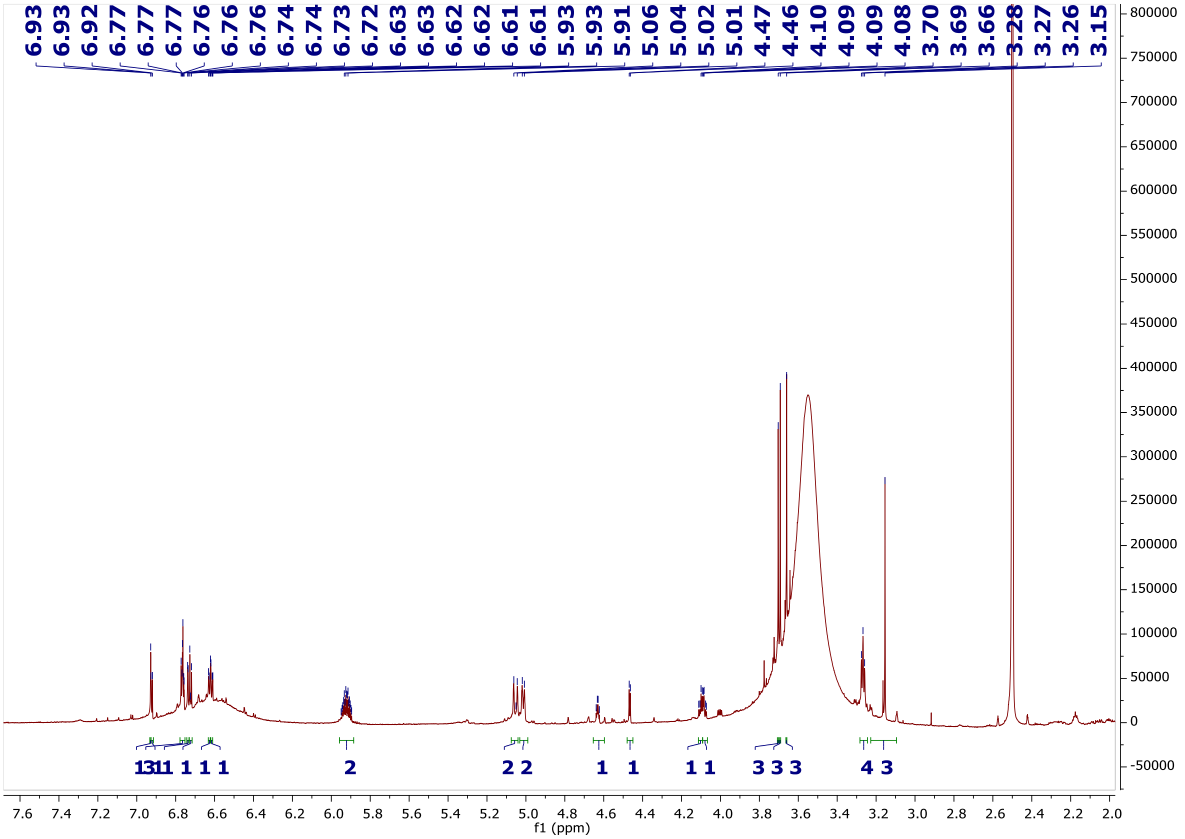

Figure S11 B: ^{1}H NMR, DMSO-d_6 at 900 MHz data for tulsinol D.

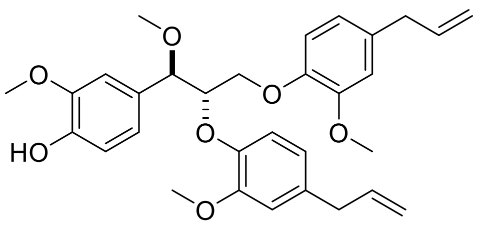

tulsinol D (^{1}H-^{13}C HSQC, DMSO-d_6 at 900 MHz)

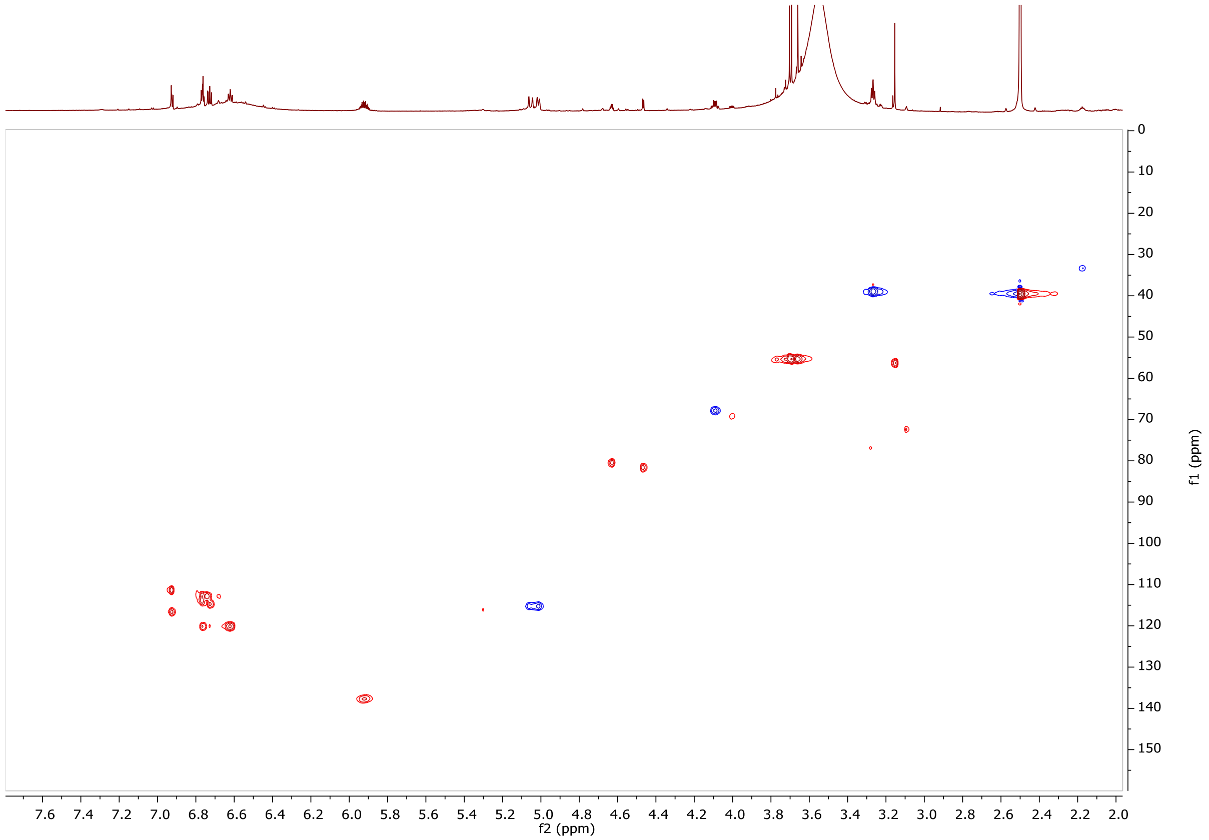

Figure S11 C: ^{1}H-^{13}C HSQC, DMSO-d_6 at 900 MHz data for tulsinol D.

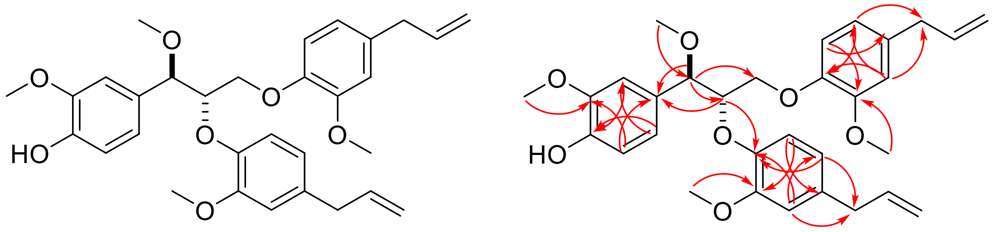

tulsinol D (^{1}H-^{13}C HMBC, DMSO-d_6 at 900 MHz)

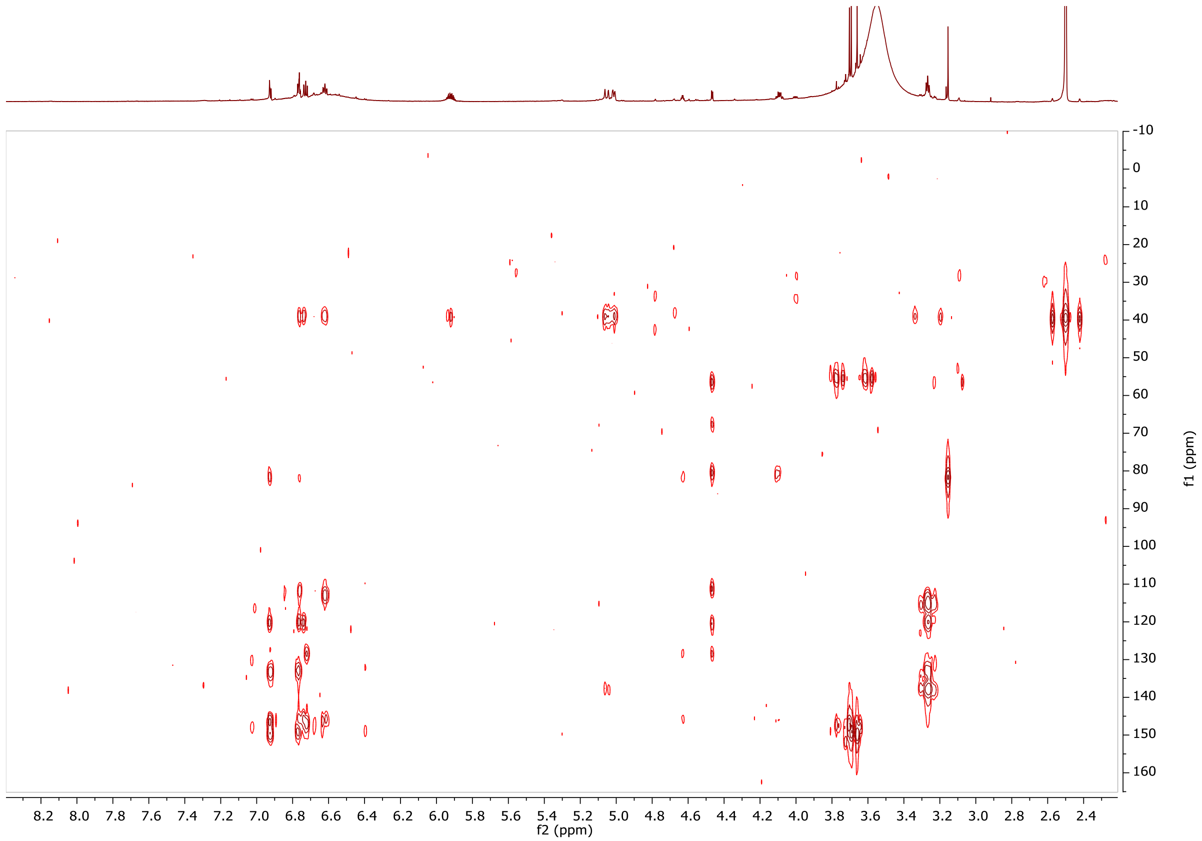

Figure S11 D: ^{1}H-^{13}C HMBC, DMSO-d_6 at 900 MHz data for tulsinol D.

The NMR Data of tulsinol F in DMSO-d_6					
Position	δ_H (J in Hz)	δ_C (C type)	Position	δ_H (J in Hz)	δ_C (C type)
1		136.7, C	1”		n.d.
2	6.93 d 2.2	113.0, CH	2”	6.40 d 2.2	105.7, CH
3		150.7, C	3”		n.d.
4		141.5, C	4”		133.3, C
5	6.79 d 7.9	120.7, CH	5”		148.1, C
6	6.72 dd 7.9, 2.2	120.5, CH	6”	6.00 d 2.2	106.1, CH
7	3.35 d 5.3	39.1, CH_2	7”	3.13 d 6.6	39.2, CH_2
8	5.96 m	137.6, CH	8”	5.81 m	137.6, CH
9	5.08 d 18.0	115.5, CH_2	9”	4.97 m	115.1, CH_2
	5.05 d 8.8			4.95 m	
1’		n.d.	3-OMe	3.67 s	55.4, CH_3
2’	6.07 d 2.2	108.4, CH	5’-OMe	3.70 s	55.5, CH_3
3’		n.d.	5”-OMe	3.74 s	55.5, CH_3
4’		130.6, C			
5’		151.7, C			
6’	6.66 d 2.2	106.6, CH			
7’	3.25 d 6.6	39.1, CH_2			
8’	5.88 m	137.3, CH			
9’	5.02 m	115.4, CH_2			
	5.01 m				

Figure S12 A: NMR Data for tulsinol F in DMSO-d_6.

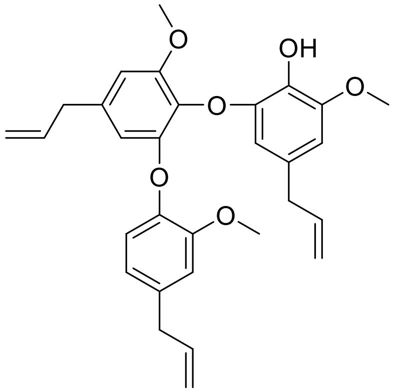

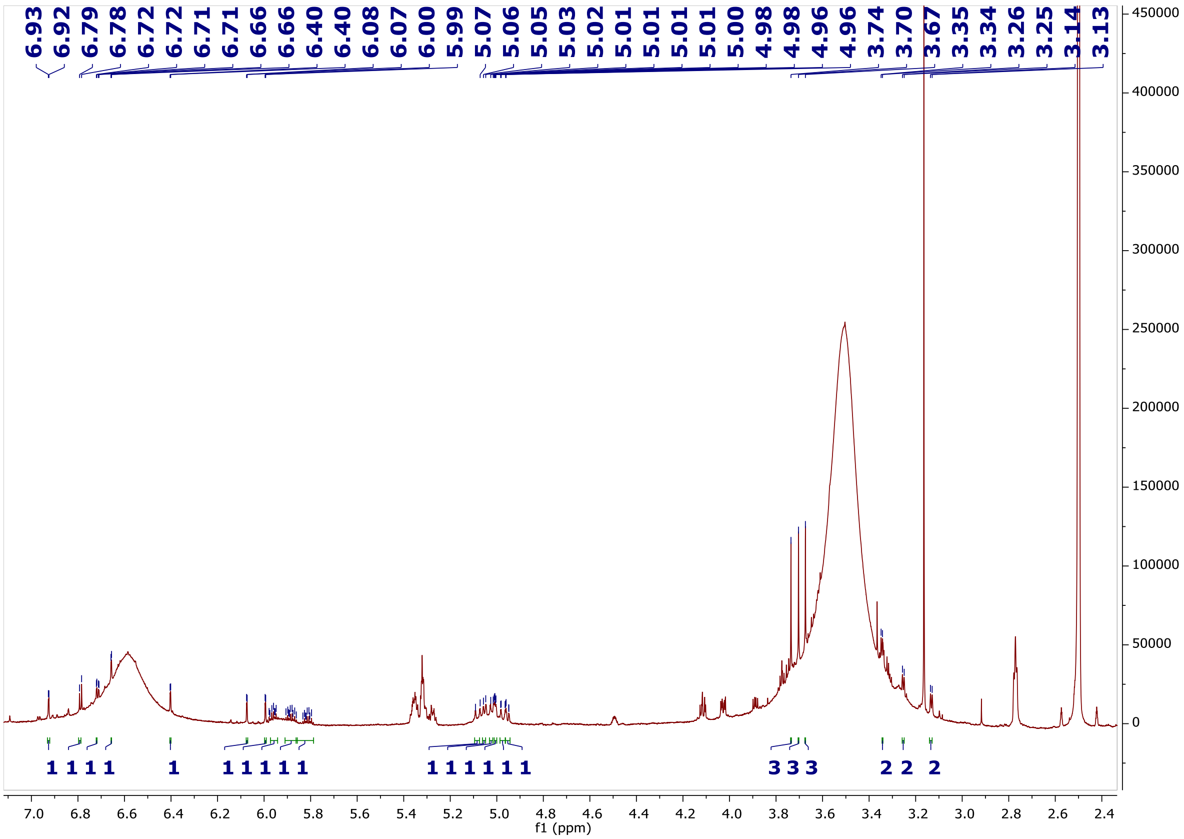

Figure S12 B: ^{1}H NMR, DMSO-d_6 at 900 MHz data for tulsinol F.

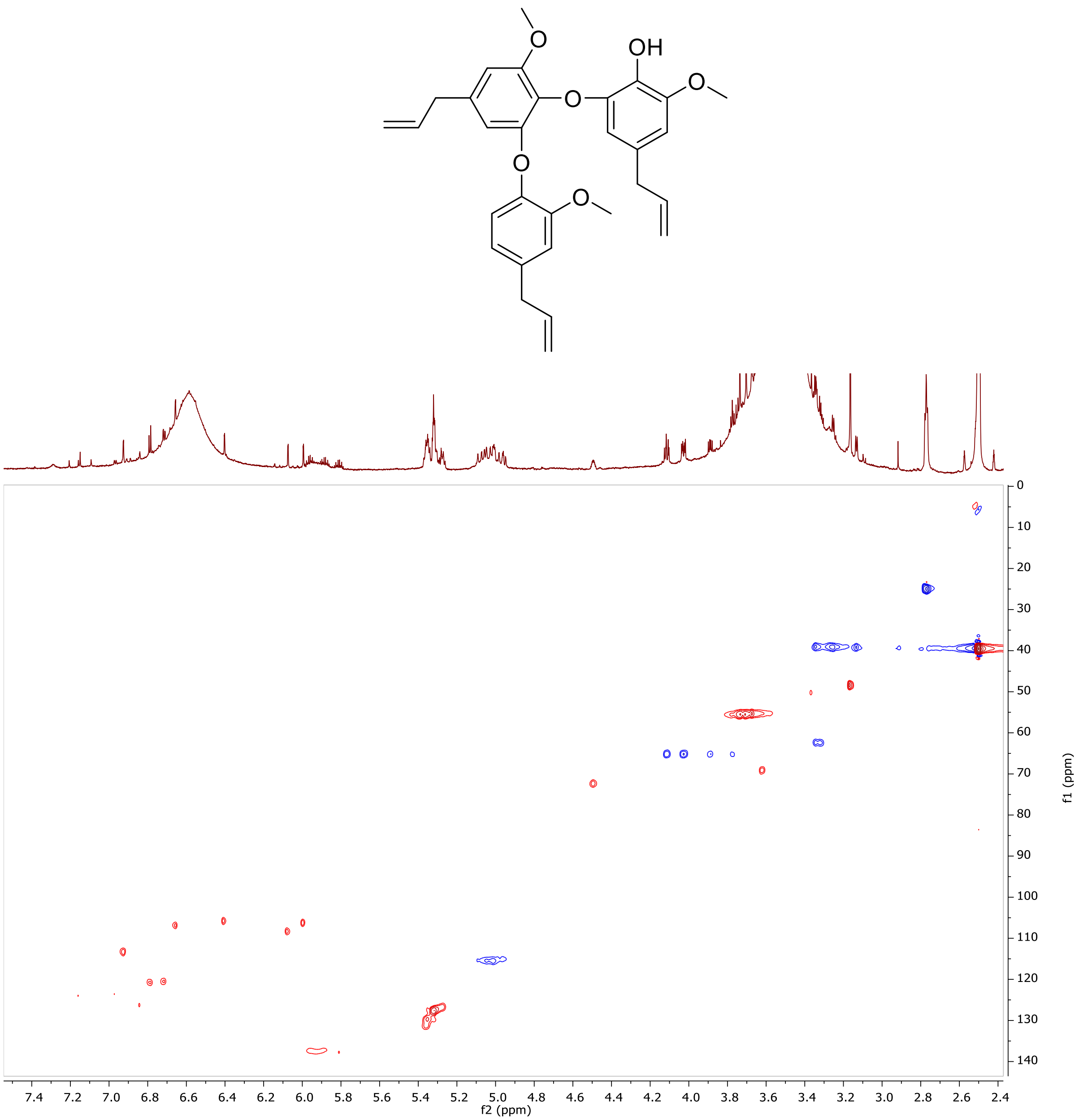

Figure S12 C: ^{1}H-^{13}C HSQC, DMSO-d_6 at 900 MHz data for tulsinol F.

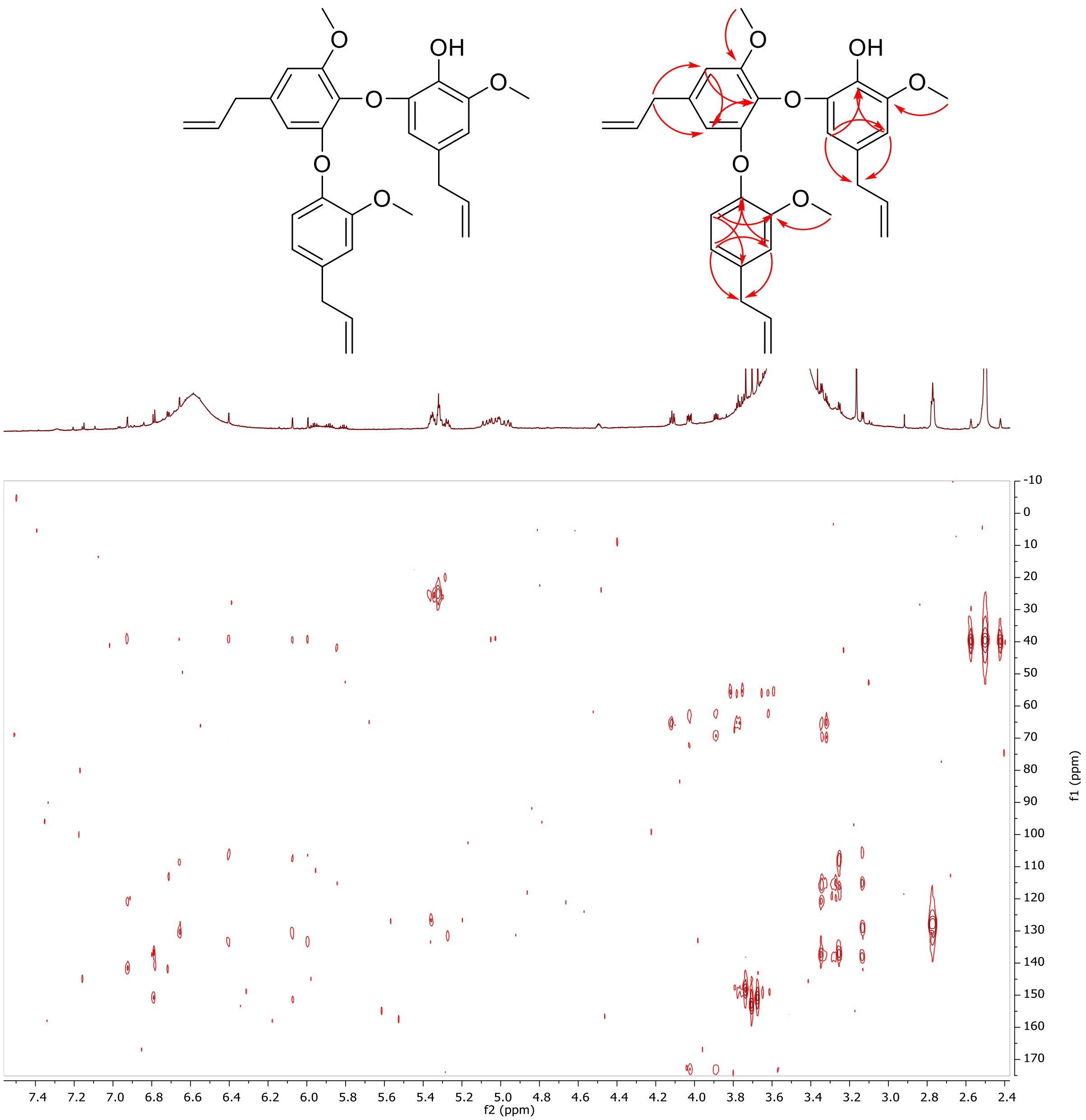

Figure S12 D: ^{1}H-^{13}C HMBC, DMSO-d_6 at 900 MHz data for tulsinol F.

The NMR Data of tulsinol H in DMSO-d_6					
Position	δ_H (J in Hz)	δ_C (C type)	Position	δ_H (J in Hz)	δ_C (C type)
1		128.9, C	1”		133.2, C
2	6.90 d 1.8	111.3, CH	2”	6.76 d 1.8	112.7, CH
3		147.1, C	3”		149.7, C
4		146.5, C	4”		146.3, C
5	6.70 d 8.0	114.9, CH	5”	6.97 d 8.3	116.0, CH
6	6.77 m	119.6, CH	6”	6.63 dd 8.3, 1.8	120.1, CH
7	4.51 d 6.2	82.6, CH	7”	3.26 m	39.3, CH_2
8	4.55 m	80.8, CH	8”	5.92 ddt 18.0, 11.0, 5.5	137.8, CH
9	4.05 dd 11.0, 4.4	68.0, CH_2	9”	5.03 m	115.3, CH_2
	3.59 dd 11.0, 4.8		4-OMe	3.61 s	55.1, CH_3
1’		133.0, C	7-OMe	3.14 s	56.3, CH_3
2’	6.77 m	112.6, CH	3’-OMe	3.72 s	55.4, CH_3
3’		148.9, C	3”-OMe	3.72 s	55.4, CH_3
4’		146.2, C			
5’	6.65 d 8.2	113.5, CH			
6’	6.59 dd 8.2, 1.8	120.1, CH			
7’	3.26 m	39.0, CH_2			

8’	5.92 ddt 18.0, 11.0, 5.5	137.8, CH			
9’	5.03 m	115.3, CH_2			

Figure S13 A: NMR Data for tulsinol H in DMSO-d_6

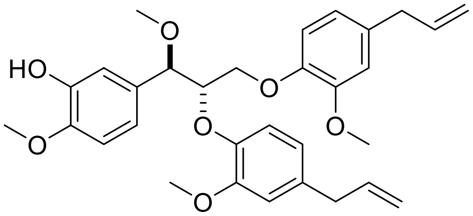

tulsinol H (^{1}H NMR, DMSO-d_6 at 900 MHz)

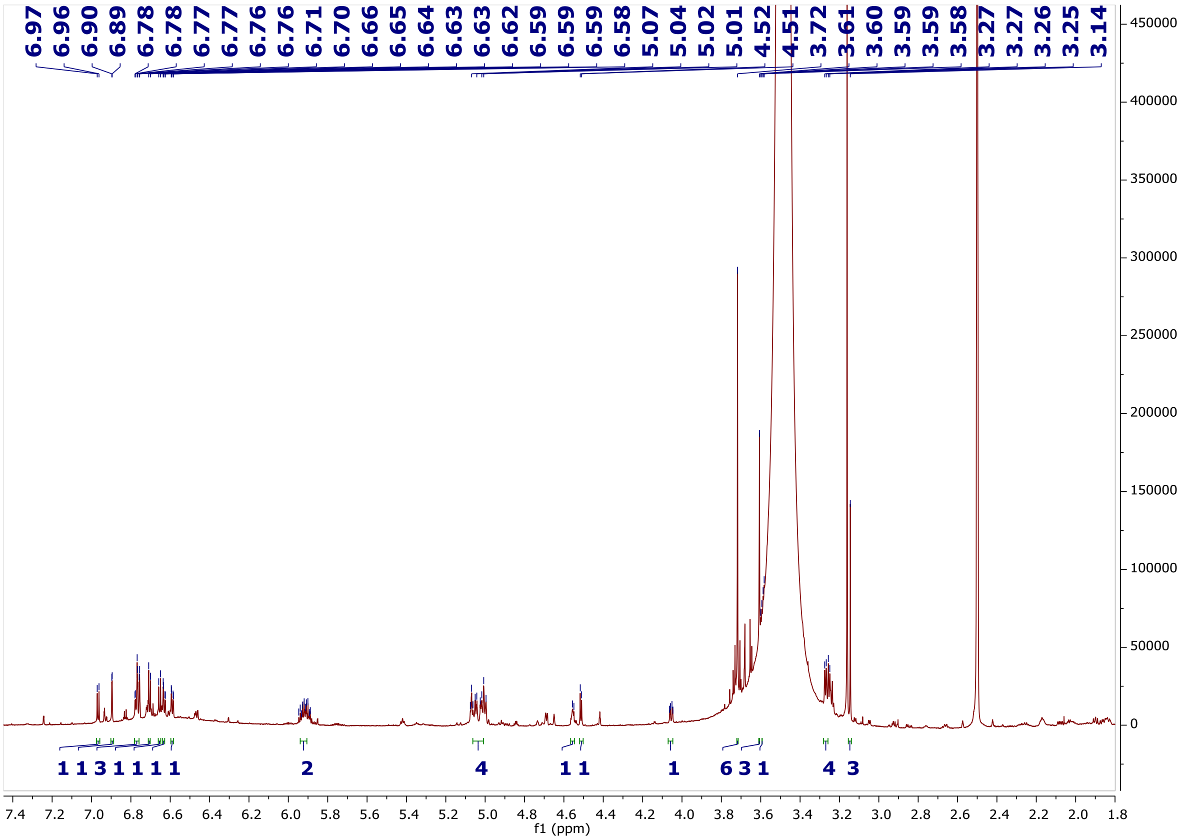

Figure S13 B: ^{1}H NMR, DMSO-d_6 at 900 MHz data for tulsinol H.

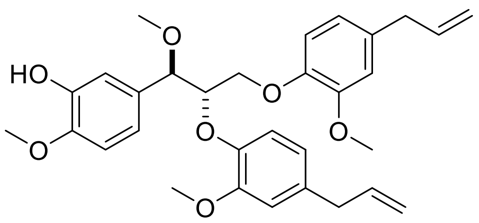

tulsinol H (^{1}H-^{13}C HSQC, DMSO-d_6 at 900 MHz)

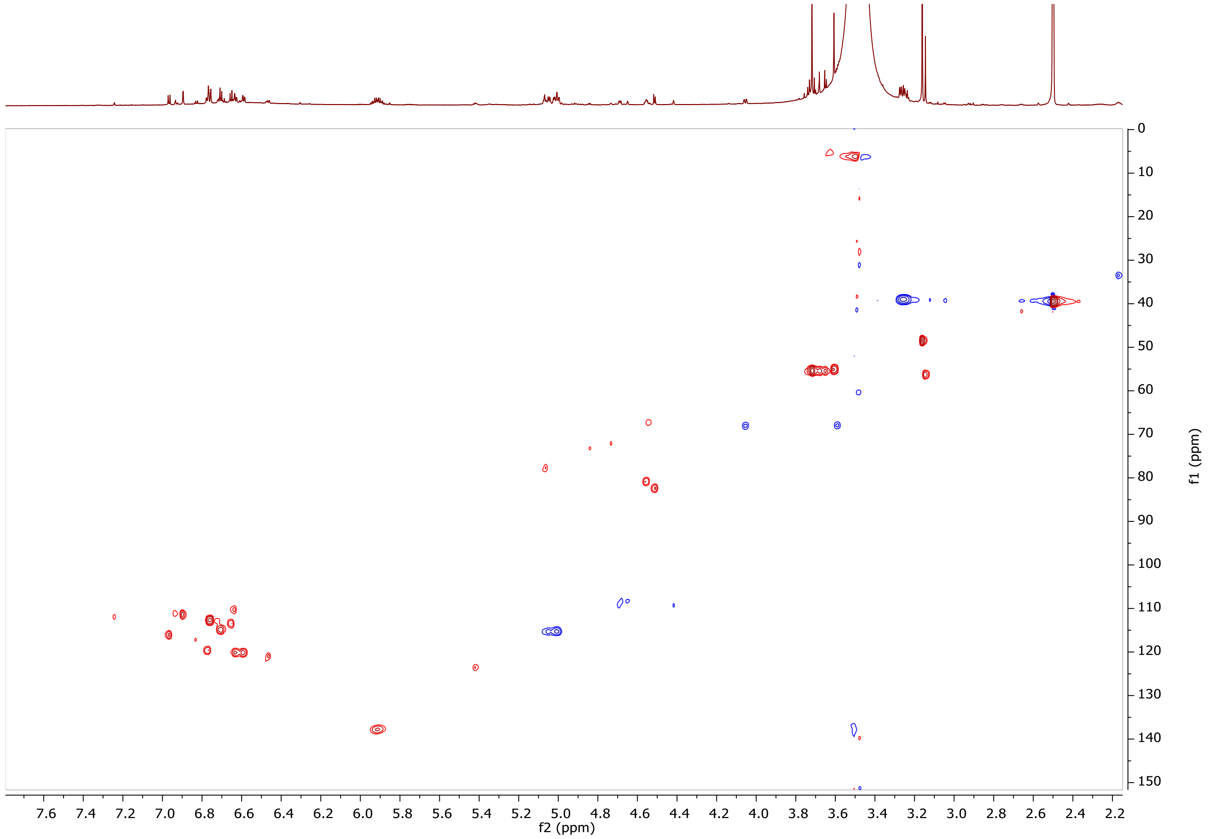

Figure S13 C: ^{1}H-^{13}C HSQC, DMSO-d_6 at 900 MHz data for tulsinol H.

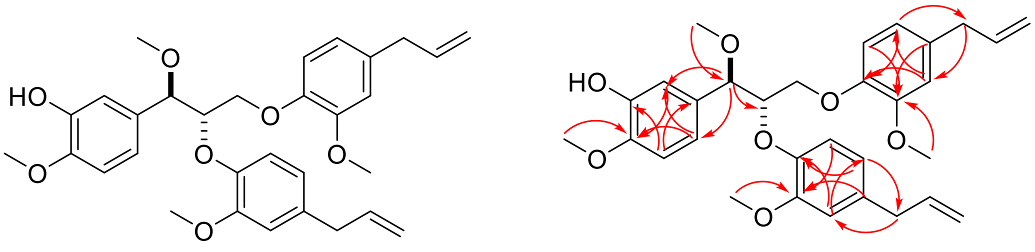

tulsinol H (^{1}H-^{13}C HMBC, DMSO-d_6 at 900 MHz)

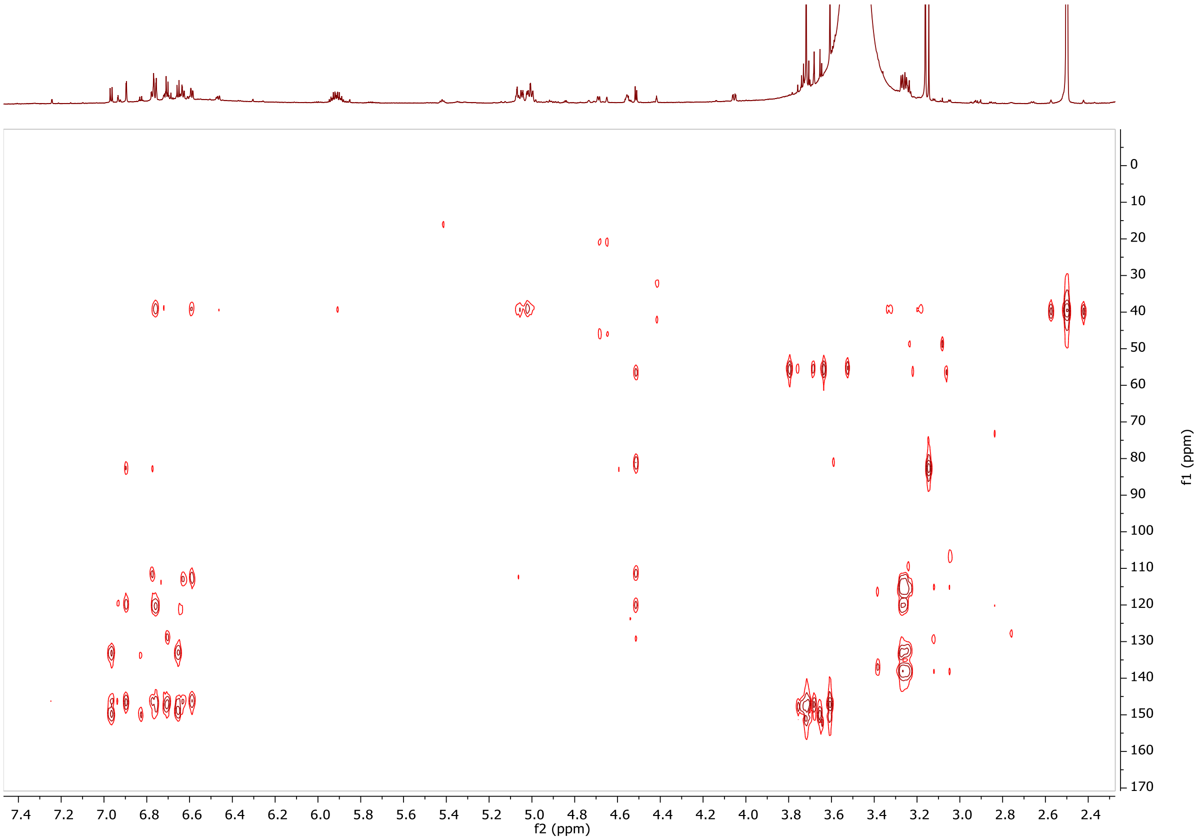

Figure S13 D: ^{1}H-^{13}C HMBC, DMSO-d_6 at 900 MHz data for tulsinol H.

The NMR Data of tulsinol I in DMSO-d_6					
Position	δ_H (*J* in Hz)	δ_C (C type)	Position	δ_H (*J* in Hz)	δ_C (C type)
1		127.7, C	1”		129.4, C
2	6.90 d 2.3	111.7, CH	2”	6.80 d 2.4	111.5, CH
3		146.9, C	3”		151.8, C
4		146.1, C	4”		143.6, C
5	6.64 d 7.9	114.2, CH	5”		n.d.
6	6.47 dd 7.9, 2.3	120.9, CH	6”	6.58 d 2.4	122.5, CH
7	4.07 d 4.0	81.7, CH	7”	3.27 d 6.8	39.0, CH_2
8	4.36 m	79.3, CH	8”	5.93 m	137.6, CH
9	3.80 m	70.2, CH_2	9”	5.08 m	115.2, CH_2
	3.57 m			5.02 m	
1’		132.3, C	1’”		135.4, C
2’	6.69 br	112.5, CH	2’”	6.79 d 2.5	110.8, CH
3’		149.1, C	3’”		148.1, C
4’		145.9, C	4’”		141.8, C
5’	6.53 m	114.4, CH	5’”		n.d.
6’	6.52 m	120.1, CH	6’”	6.52 m	122.4, CH
7’	3.25 d 6.6	39.0, CH_2	7’”	3.32 d 6.8	39.1, CH_2
8’	5.93 m	137.6, CH	8’”	5.93 m	137.6, CH
9’	5.06 m	115.2, CH_2	9’”	5.10 m	115.2, CH_2
	4.99 m			5.04 m	
3-OMe	3.70 s	55.2, CH_3	3”-OMe	3.72 s	55.5, CH_3
7-OMe	2.97 s	55.6, CH_3	3’”-OMe	3.80 s	55.6, CH_3
3’-OMe	3.61 s	55.2, CH_3			

Figure S14 A: NMR Data for tulsinol I in DMSO-d_6.

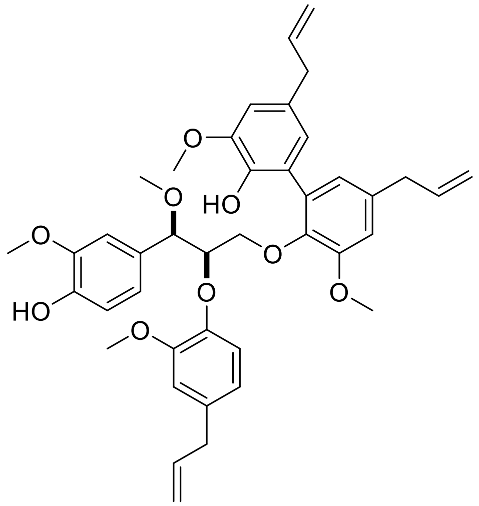

tulsinol I (^{1}H NMR, DMSO-d_6 at 900 MHz)

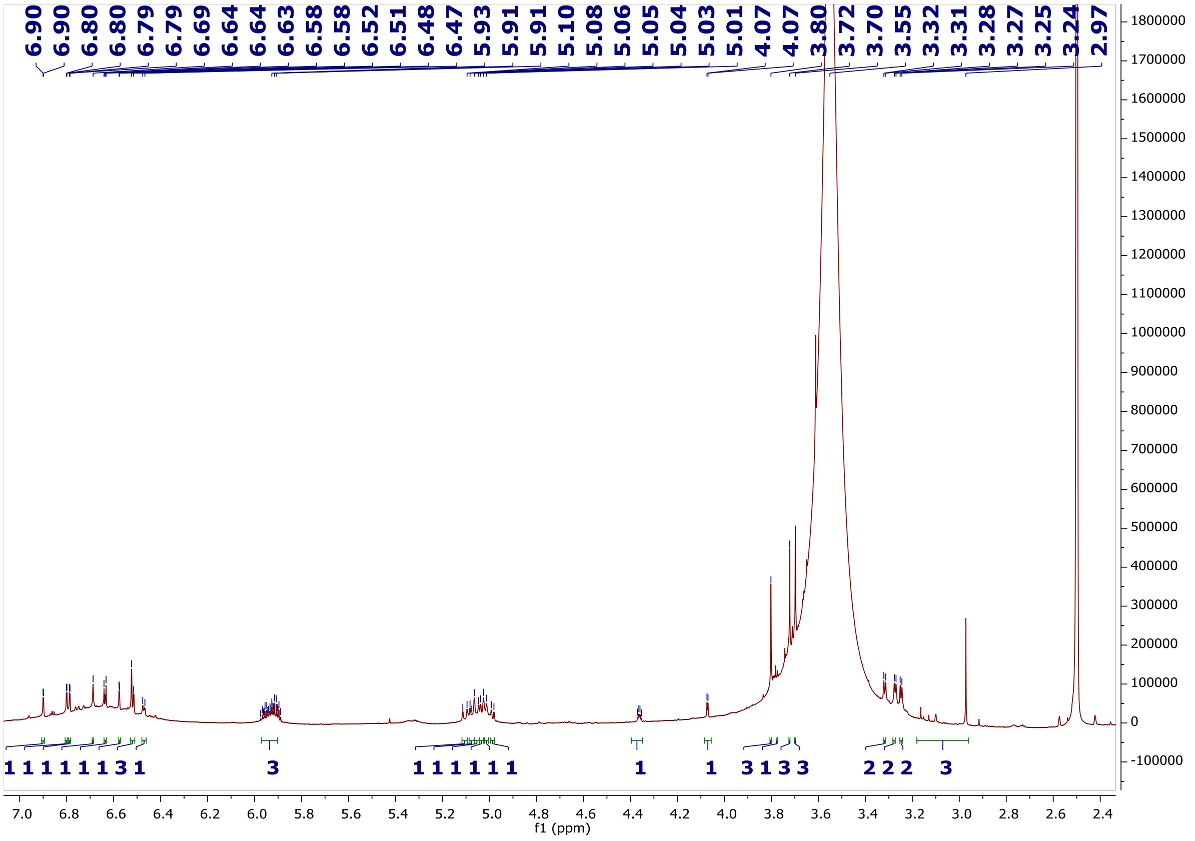

Figure S14 B: ^{1}H NMR, DMSO-d_6 at 900 MHz data for tulsinol I.

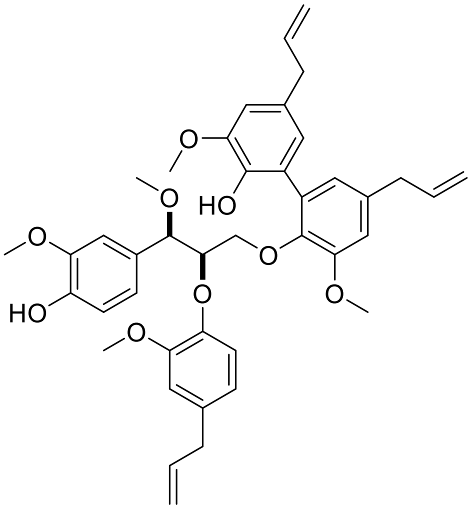

tulsinol I (^{1}H-^{13}C HSQC, DMSO-d_6 at 900 MHz)

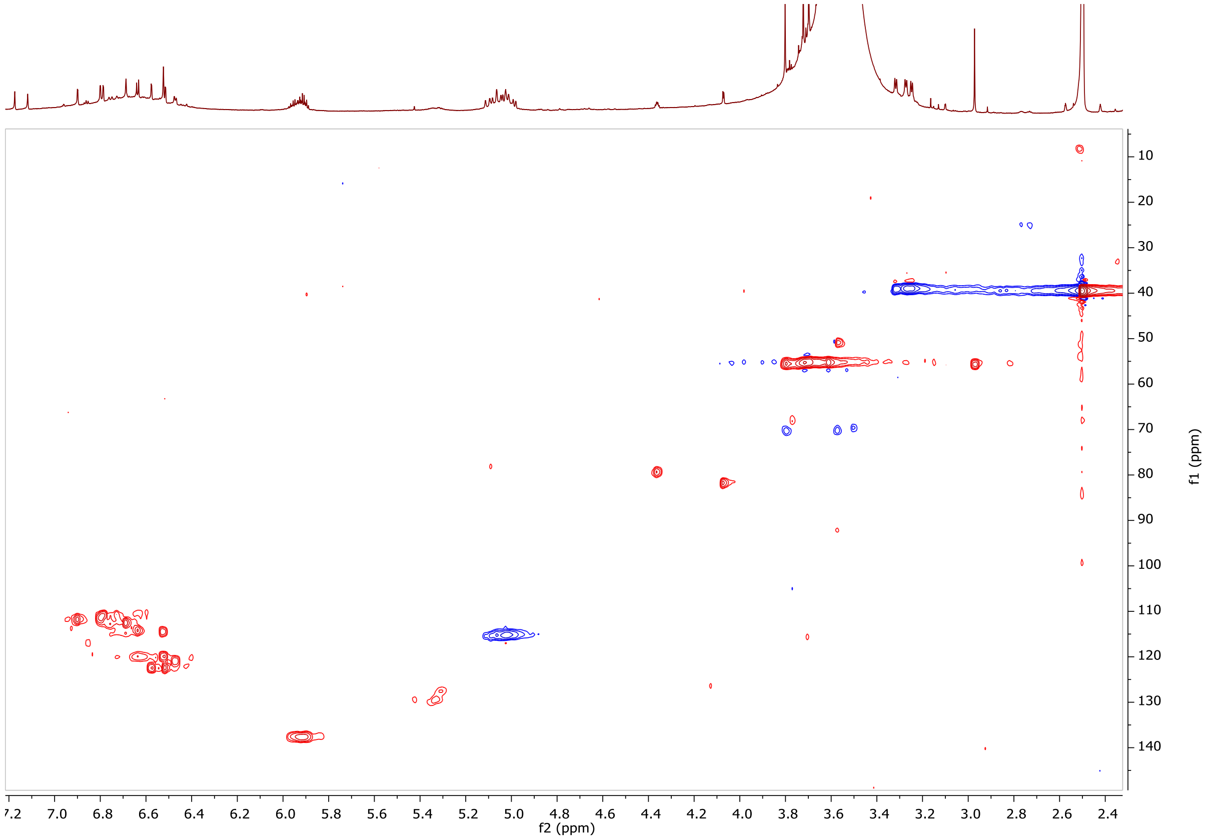

Figure S14 C: ^{1}H-^{13}C HSQC, DMSO-d_6 at 900 MHz data for tulsinol I.

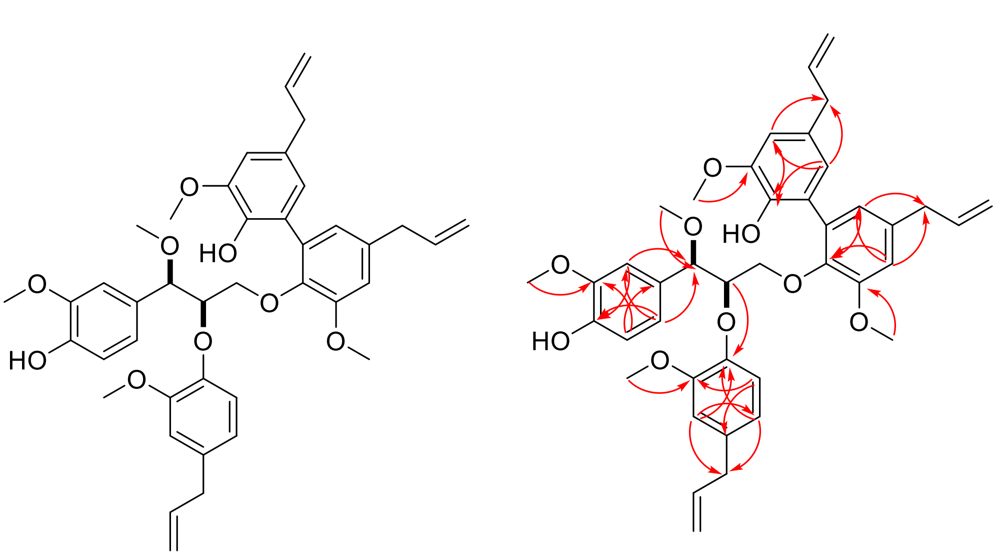

Tulsinol I (^{1}H-^{13}C HMBC, DMSO-d_6 at 900 MHz)

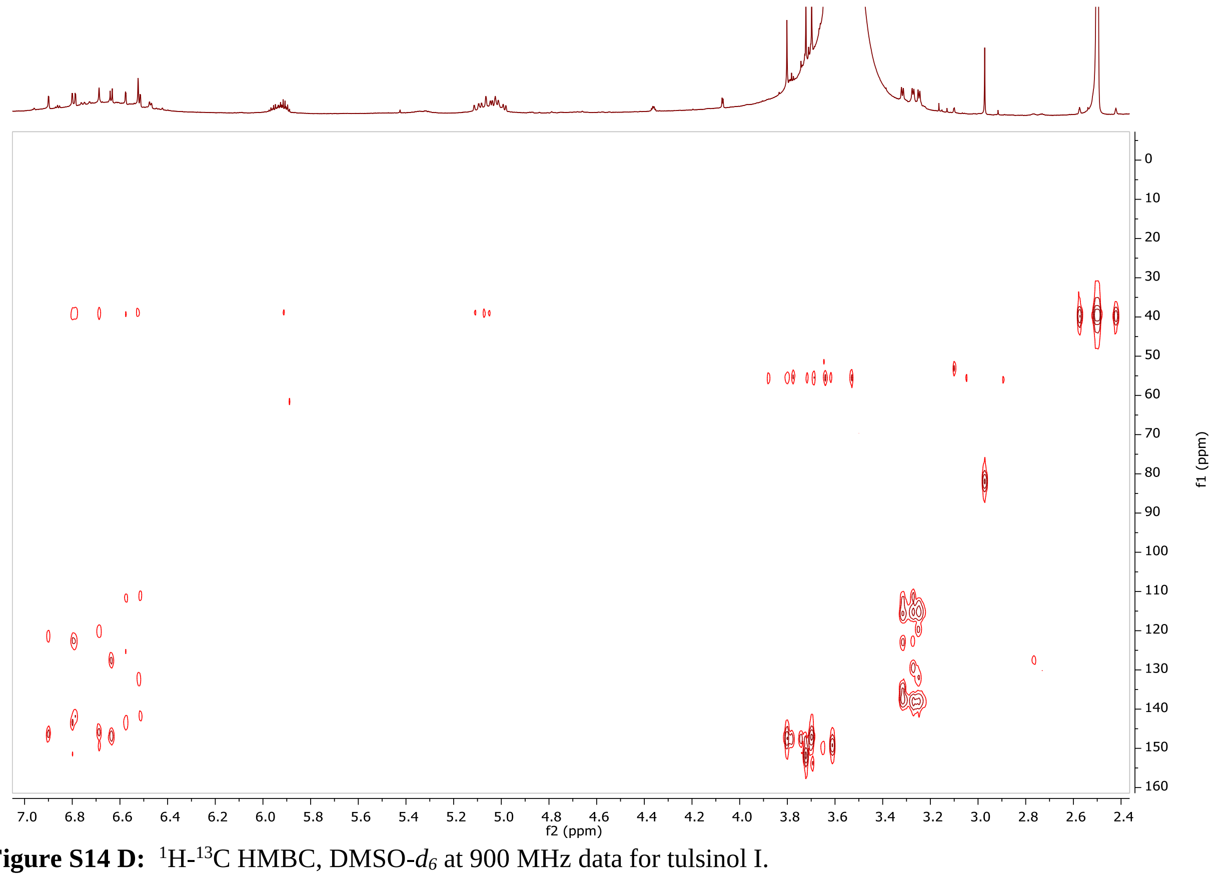

Figure S14 D: ^{1}H-^{13}C HMBC, DMSO-d_6 at 900 MHz data for tulsinol I.

The NMR Data of tulsinol J in DMSO-d_6					
Position	δ_H (*J* in Hz)	δ_C (C type)	Position	δ_H (*J* in Hz)	δ_C (C type)
1		129.1, C	1”		129.4, C
2	6.86 d 2.2	111.6, CH	2”	6.81 d 2.2	111.9, CH
3		147.1, C	3”		151.8, C
4		145.8, C	4”		143.8, C
5	6.64 d 8.4	114.5, CH	5”		n.d.
6	6.54 dd 8.4, 2.2	119.9, CH	6”	6.57 d 2.2	122.5, CH
7	4.08 d 3.3	80.8, CH	7”	3.26 d 7.0	39.0, CH_2
8	4.09 dt 3.3, 5.5	80.5, CH	8”	5.88 ddt 16.7, 9.2, 7.0	137.7, CH
9	3.87 dd 10.3, 5.5	70.4, CH_2	9”	5.03 m	115.1, CH_2
	3.80 m			5.00 m	
1’		132.1, C	1’”		134.9, C
2’	6.67 d 2.2	112.4, CH	2’”	6.77 d 2.6	110.7, CH
3’		148.8, C	3’”		148.3, C
4’		146.1, C	4’”		142.1, C
5’	6.29 d 8.4	113.7, CH	5’”		n.d.
6’	6.44 dd 8.4, 2.2	119.8, CH	6’”	6.50 d 2.6	122.4, CH
7’	3.22 d 7.0	39.0, CH_2	7’”	3.32 d 6.6	39.1, CH_2
8’	5.88 ddt 16.7, 9.2, 7.0	137.7, CH	8’”	5.95 ddt 17.2, 9.7, 6.6	137.5, CH
9’	5.03 m	115.1, CH_2	9’”	5.11 m	115.1, CH_2
	4.96 m			5.06 m	
3-OMe	3.66 s	55.2, CH_3	3”-OMe	3.75 s	55.4, CH_3
7-OMe	3.01 s	56.2, CH_3	3’”-OMe	3.79 s	55.5, CH_3
3’-OMe	3.66 s	55.2, CH_3			

Figure S15 A: NMR Data for tulsinol J in DMSO-d_6.

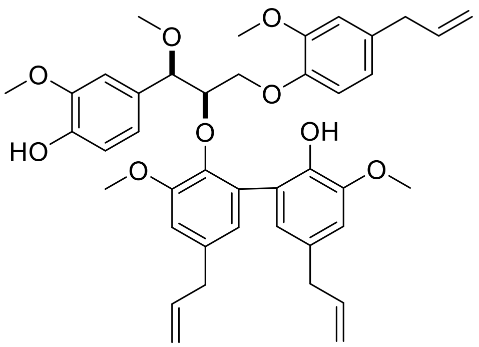

tulsinol J (^{1}H NMR, DMSO-d_6 at 900 MHz)

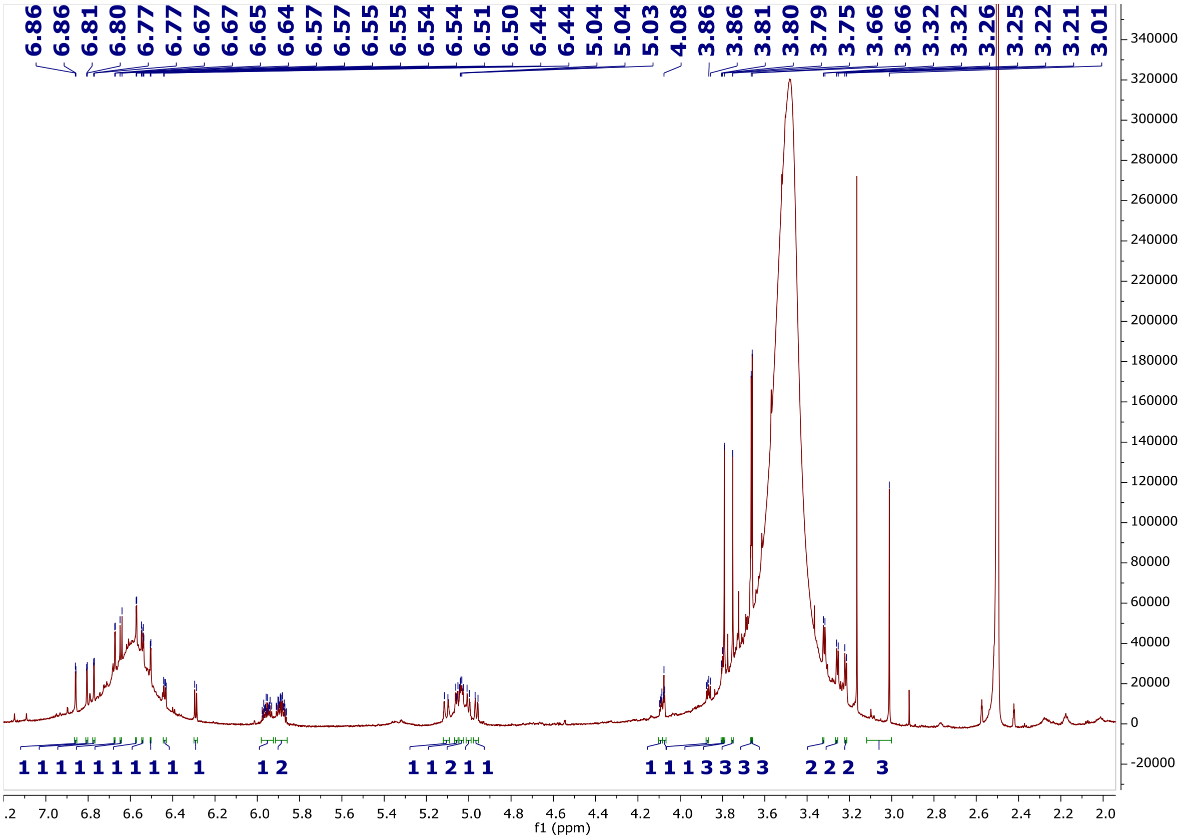

Figure S15 B: ^{1}H NMR, DMSO-d_6 at 900 MHz data for tulsinol J.

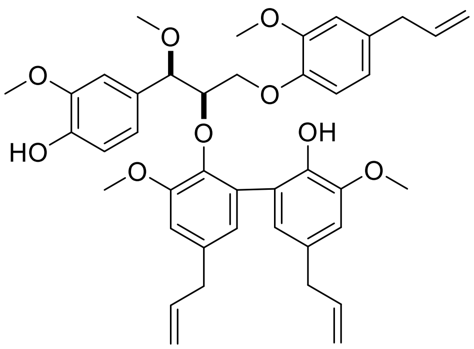

tulsinol J (^{1}H-^{13}C HSQC, DMSO-d_6 at 900 MHz)

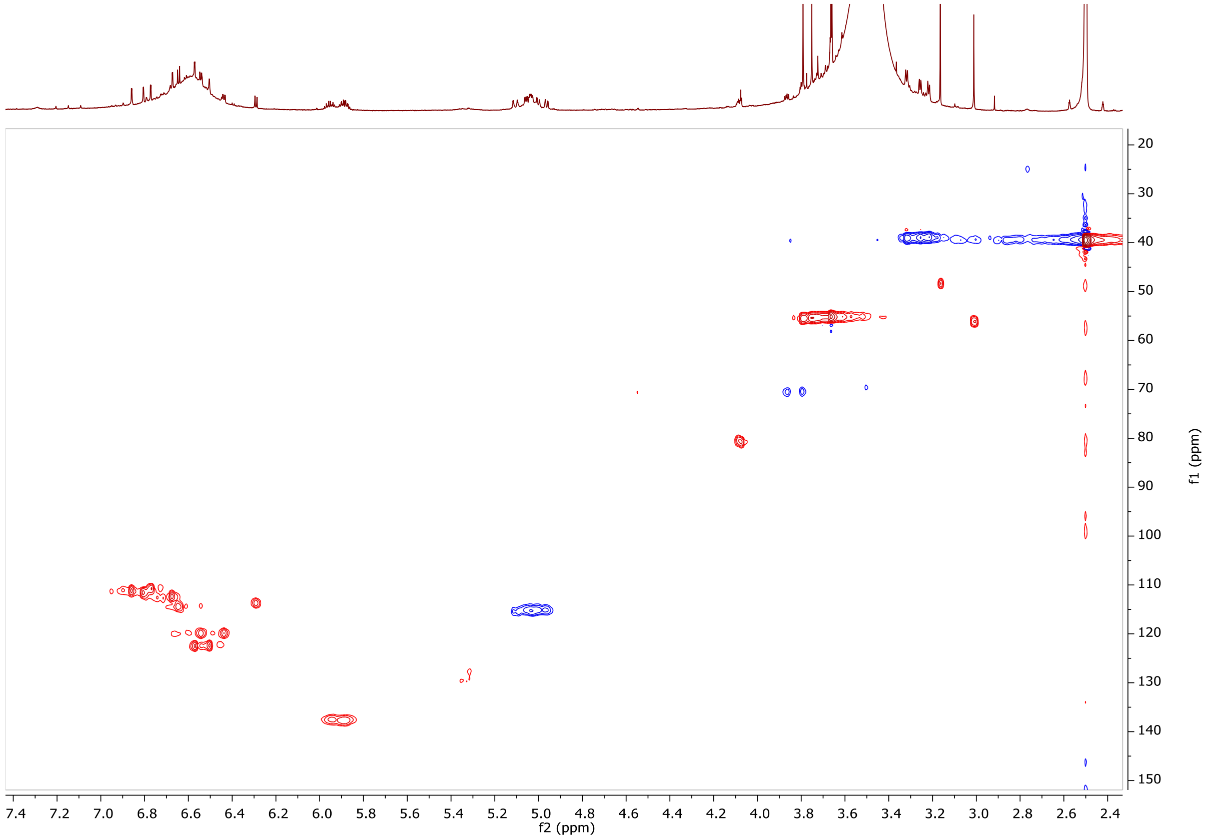

Figure S15 C: ^{1}H-^{13}C HSQC, DMSO-d_6 at 900 MHz data for tulsinol J.

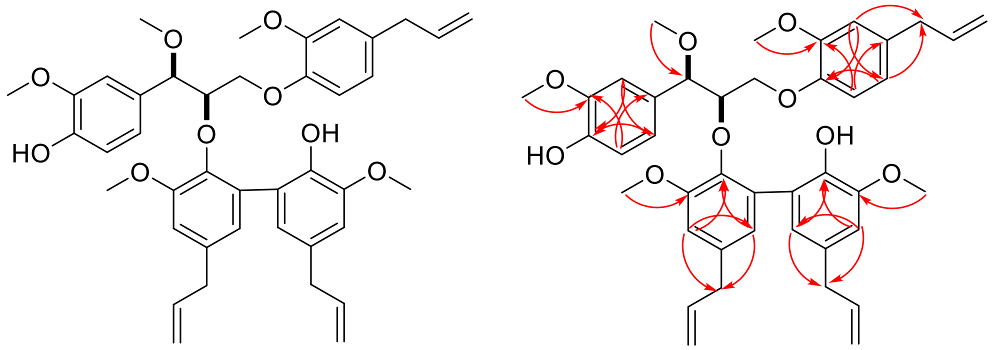

compound J (^{1}H-^{13}C HMBC, DMSO-d_6 at 900 MHz)

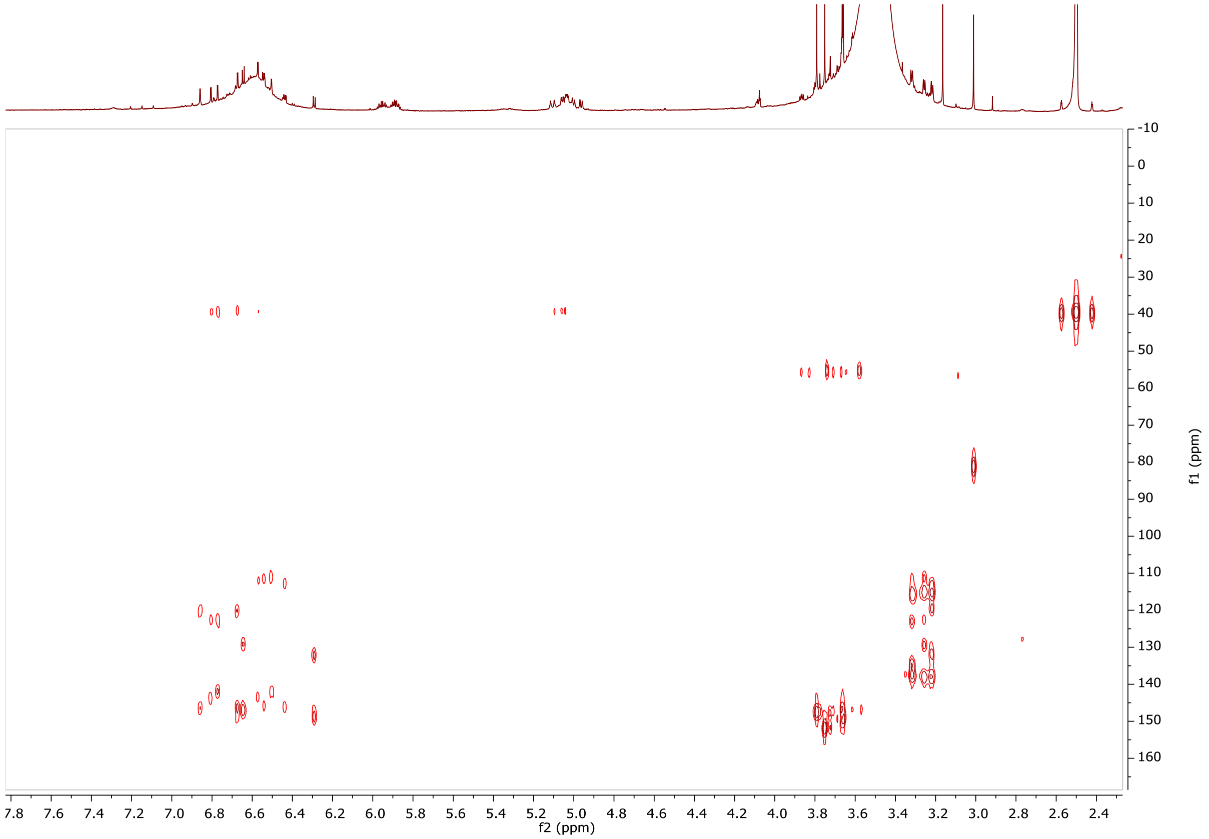

Figure S15 D: ^{1}H-^{13}C HMBC, DMSO-d_6 at 900 MHz data for tulsinol J.

The NMR Data of valeraninium B in DMSO-d_6					
Position	δ_H (*J* in Hz)	δ_C (C type)	Position	δ_H (*J* in Hz)	δ_C (C type)
1		126.1, C	1”		137.4, C
2	6.94 d 8.4	129.5, CH	2”	2.05 m	41.3, CH_2
3	6.65 d 8.4	115.1, CH		1.95 m	
4		156.4, C	3”	1.77 m	44.6, CH
5	6.65 d 8.4	115.1, CH	4”	2.31 m	49.7, CH
6	6.94 d 8.4	129.5, CH	5”		135.2, C
7	3.14 m	35.6, CH_2	6”	0.79 d 7.9	26.3, CH
	3.09 m		7”	0.98 m	27.3, CH
8	4.67 m	61.1, CH_2	8”	1.96 m	22.5, CH_2
	4.74 m			1.04 m	
1’	8.63 s	136.6, CH	9”	1.67 m	34.4, CH_2
2’		148.1, C		1.38 m	
3’	3.37 m	37.6, CH	10”	2.08 m	38.1, CH
4’	2.41 m	33.2, CH_2	11”		20.3, C
	1.66 m		12”	1.08 s	27.8, CH_3
5’	3.16 m	30.2, CH_2	13”	0.81 s	17.2, CH_3
	3.00 m		14”	0.90 d 7.0	21.6, CH_3
6’		162.5, C	15”	0.96 d 6.6	17.2, CH_3
7’		137.6, C			
8’	8.68 s	141.2, CH			
9’	1.25 d 6.8	18.7, CH_3			
10’	2.97 dd 13.9, 4.6	34.2, CH_2			
	2.64 dd 13.9, 10.1				

Figure S16 A: NMR Data for valeraninium A in DMSO-d_6.

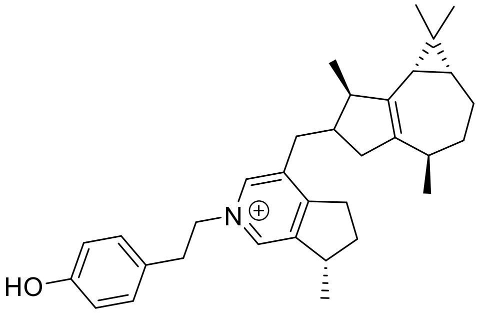

valeraninium A (^{1}H NMR, DMSO-d_6 at 900 MHz)

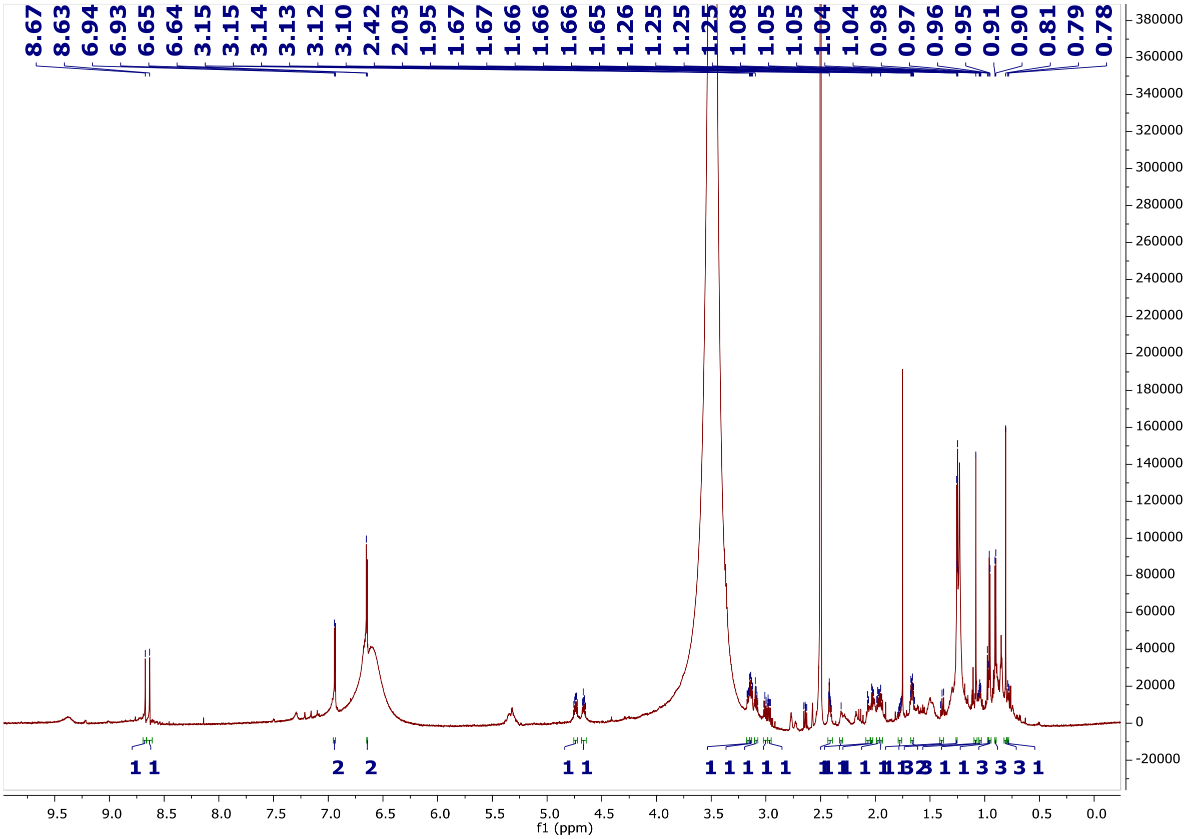

Figure S16 B: ^{1}H NMR, DMSO-d_6 at 900 MHz data for valeraninium A.

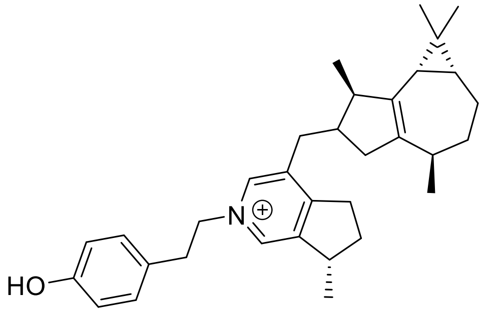

valeraninium A (^{1}H-^{13}C HSQC, DMSO-d_6 at 900 MHz)

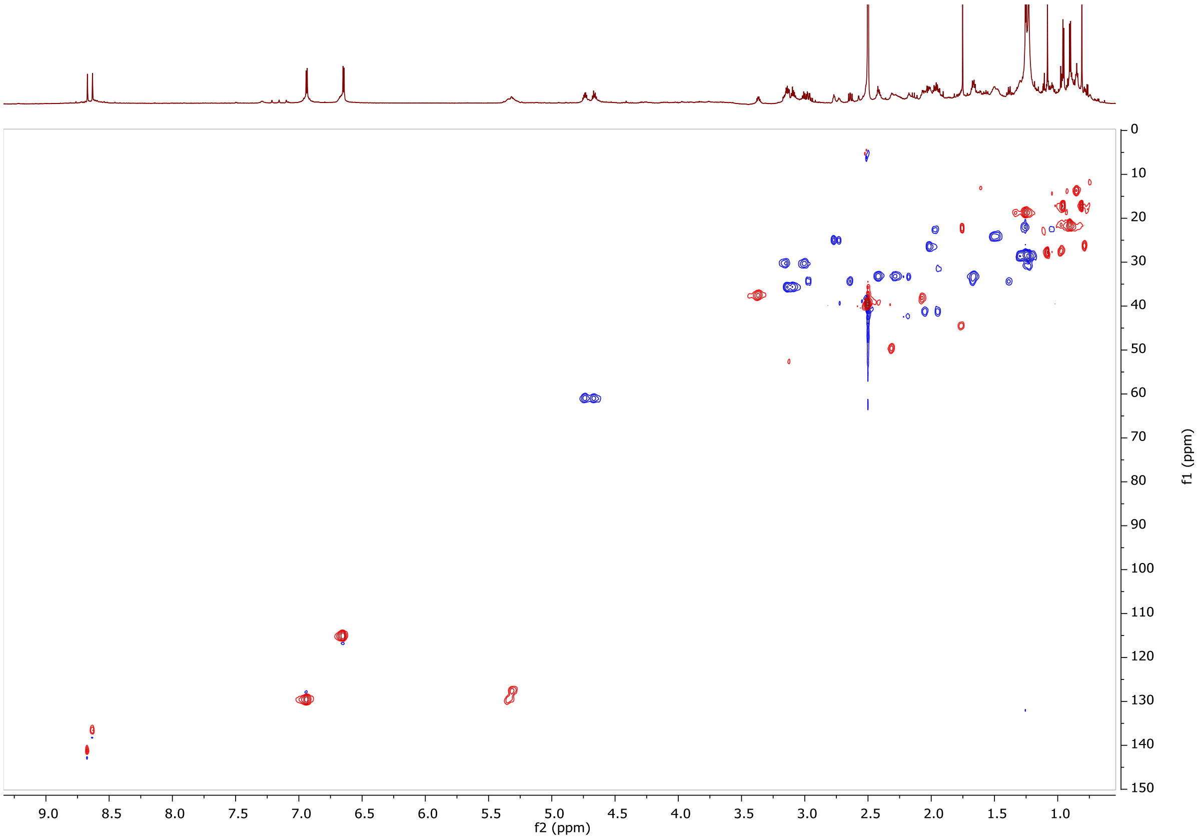

Figure S16 C: ^{1}H-^{13}C HSQC, DMSO-d_6 at 900 MHz data for valeraninium A.

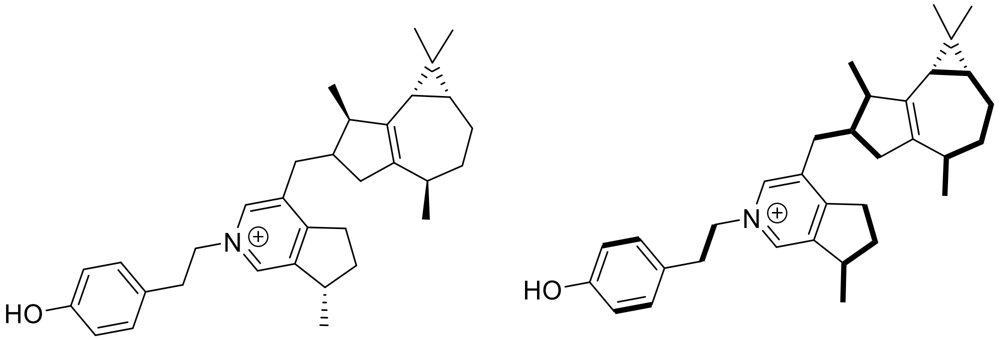

valeraninium A (^{1}H-^{1}H COSY, DMSO-d_6 at 900 MHz)

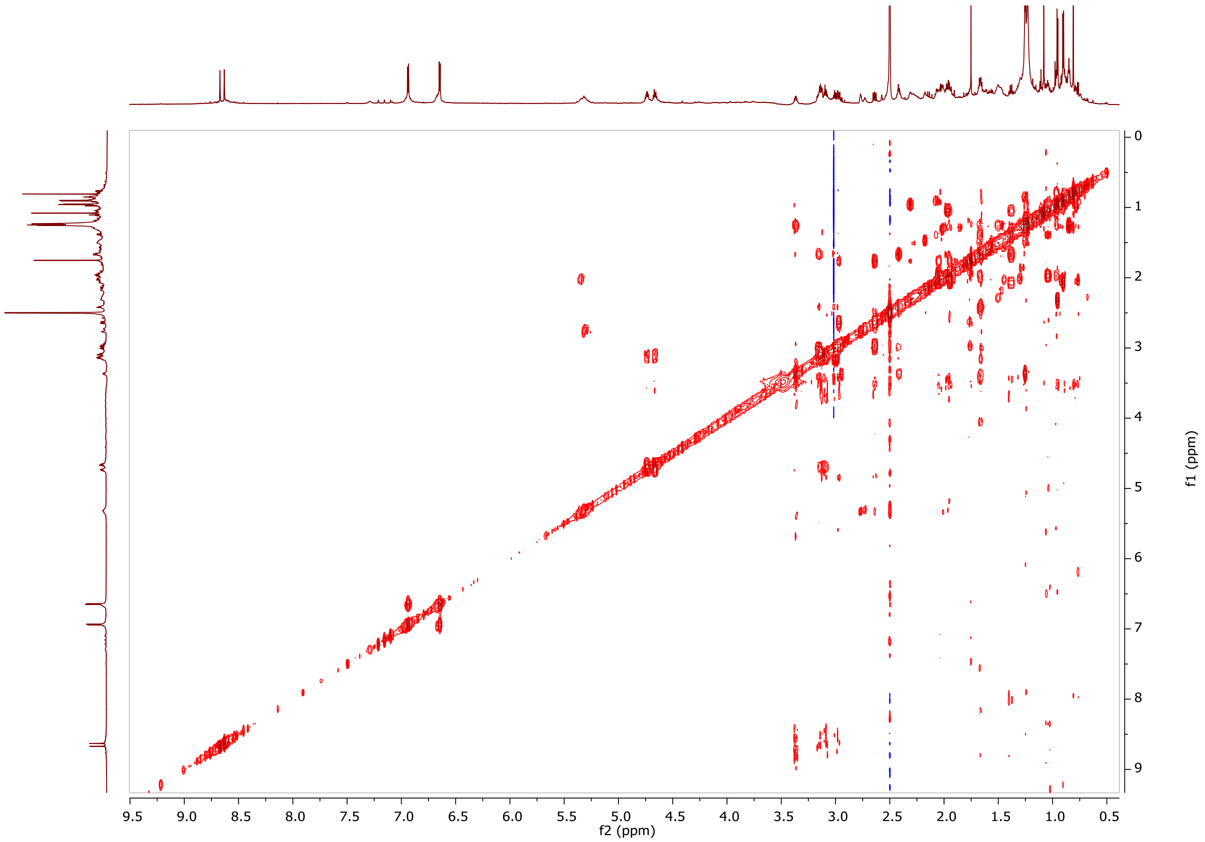

Figure S16 D: ^{1}H-^{13}C COSY, DMSO-d_6 at 900 MHz data for valeraninium A.

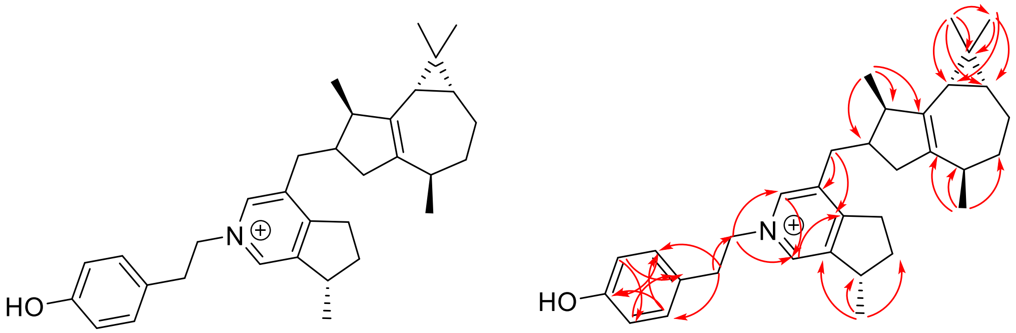

valeraninium A (^{1}H-^{13}C HMBC, DMSO-d_6 at 900 MHz)

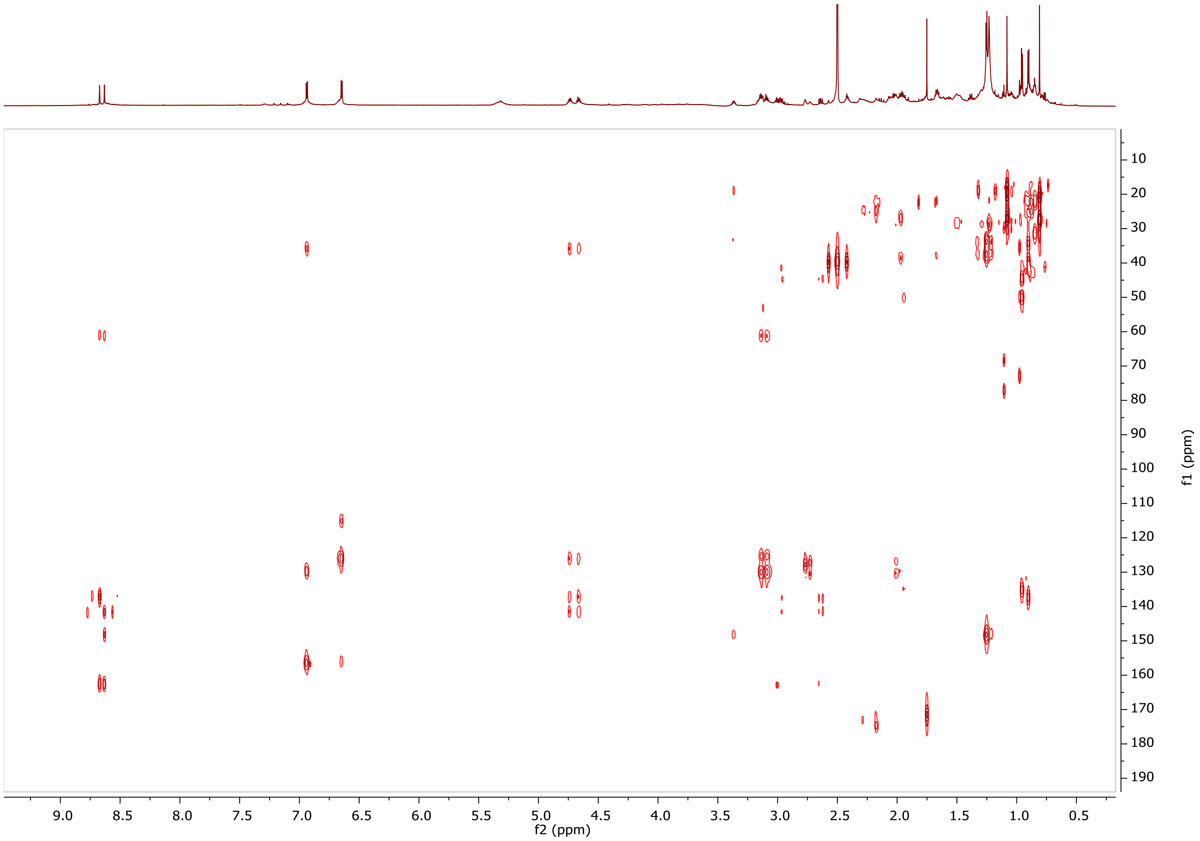

Figure S16 E: ^{1}H-^{13}C HMBC, DMSO-d_6 at 900 MHz data for valeraninium A.

The NMR Data of valeraninium B in DMSO-d_6					
Position	δ_H (J in Hz)	δ_C (C type)	Position	δ_H (J in Hz)	δ_C (C type)
1		125.9, C	1”		150.3, C
2	6.92 d 8.4	129.5, CH	2”	5.24 m	124.6, CH
3	6.65 d 8.4	115.1, CH	3”	1.89 m	41.5, CH_2
4		156.4, C		1.78 m	
5	6.65 d 8.4	115.1, CH	4”		45.8, C
6	6.92 d 8.4	129.5, CH	5”	2.91 d 6.6	51.8, CH
7	3.14 m	35.7, CH_2	6”	0.53 dd 9.9, 6.6	27.4, CH
	3.08 m		7”	0.83 m	26.2, CH
8	4.76 m	60.9, CH_2	8”	1.94 m	21.3, CH_2
	4.69 m			1.03 m	
1’	8.69 s	136.6, CH	9”	1.66 m	33.1, CH_2
2’		148.4, C		1.24 m	
3’	3.38 m	37.6, CH	10”	2.54 m	36.8, CH
4’	2.40 m	33.3, CH_2	11”		19.2, C
	1.63 m		12”	1.05 s	14.4, CH_3
5’	3.05 m	31.2, CH_2	13”	0.96 s	30.2, CH_3
	2.96 m		14”	0.86 d 7.0	22.5, CH_3
6’		163.9, C	15”	0.94 s	22.1, CH_3
7’		134.0, C			
8’	8.45 s	142.6, CH			
9’	1.28 d 7.0	18.6, CH_3			
10’	2.62 s	41.7, CH_2			

Figure S17 A: NMR Data for valeraninium B in DMSO-d_6.

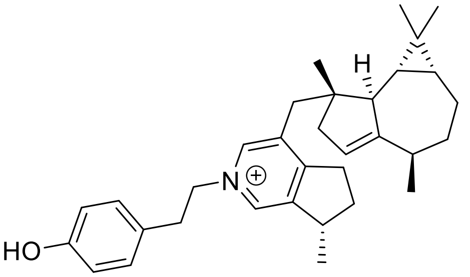

valeraninium B (^{1}H NMR, DMSO-d_6 at 900 MHz)

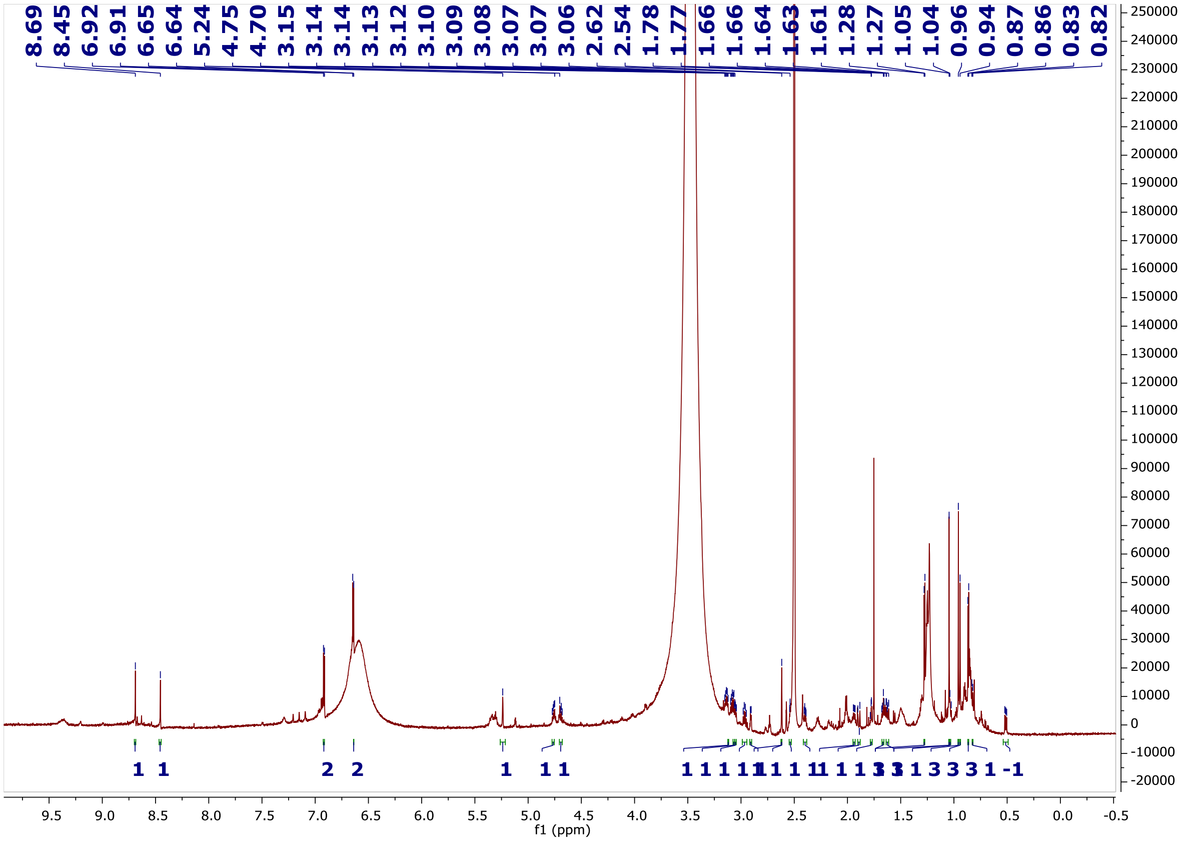

Figure S17 B: ^{1}H NMR, DMSO-d_6 at 900 MHz data for valeraninium B.

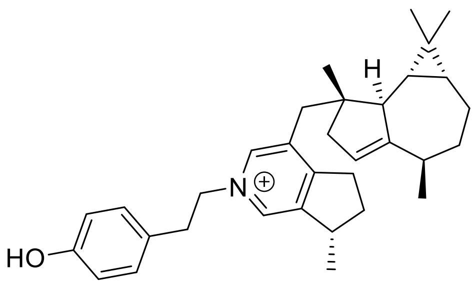

valeraninium B (^{1}H-^{13}C HSQC, DMSO-d_6 at 900 MHz)

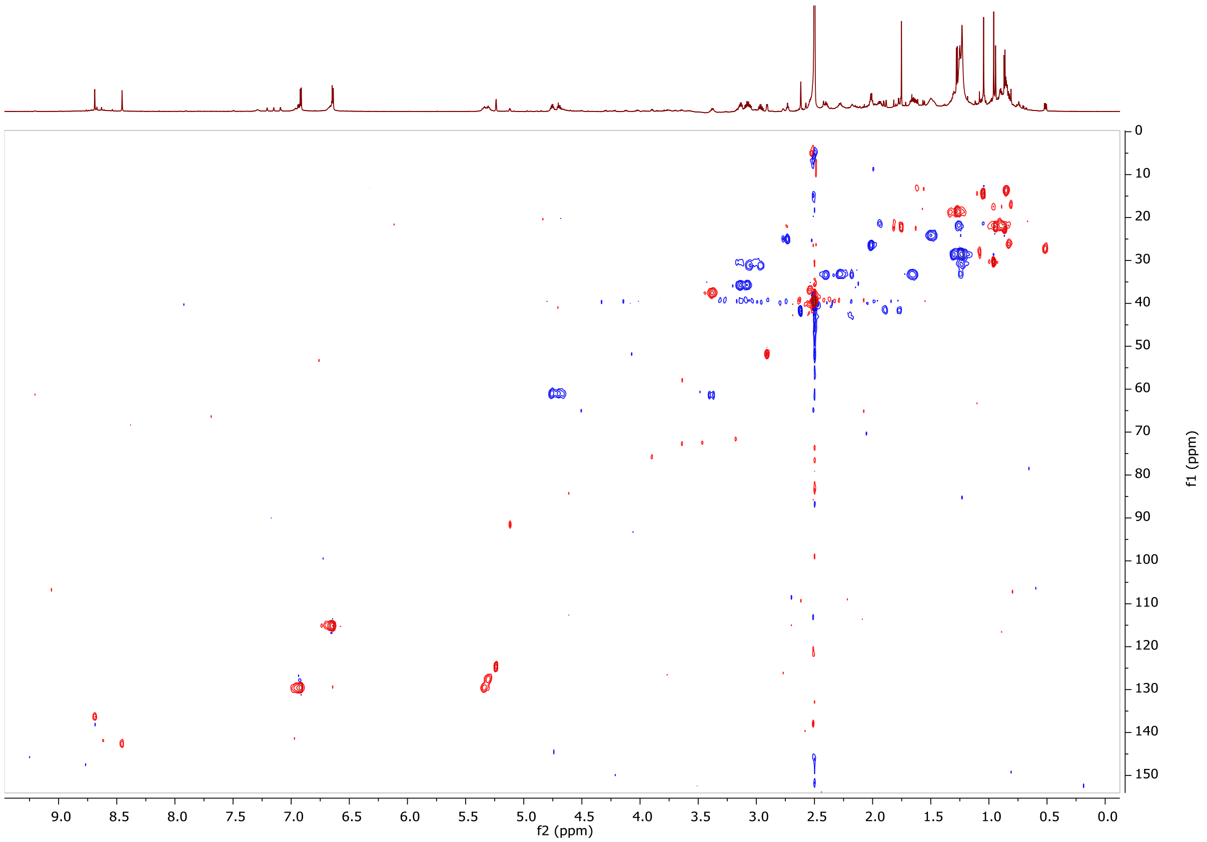

Figure S17 C: ^{1}H-^{13}C HSQC, DMSO-d_6 at 900 MHz data for valeraninium B.

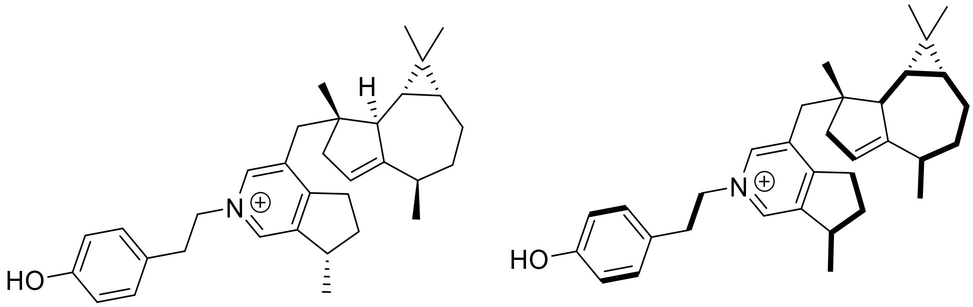

valeraninium B (^{1}H-^{1}H COSY, DMSO-d_6 at 900 MHz)

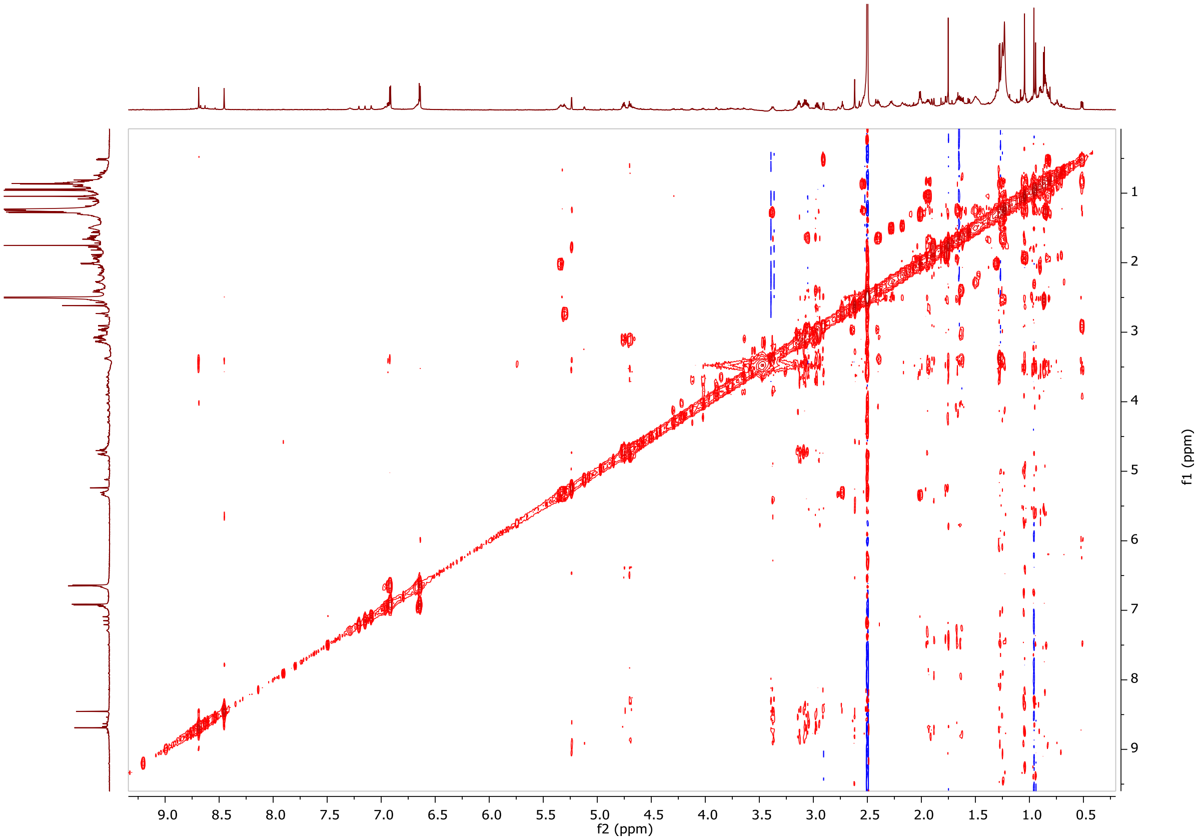

Figure S17 D: ^{1}H-^{13}C COSY, DMSO-d_6 at 900 MHz data for valeraninium B.

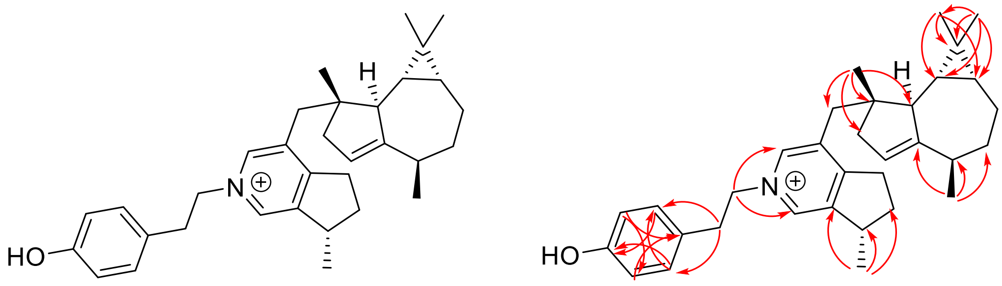

valeraninium B (^{1}H-^{13}C HMBC, DMSO-d_6 at 900 MHz)

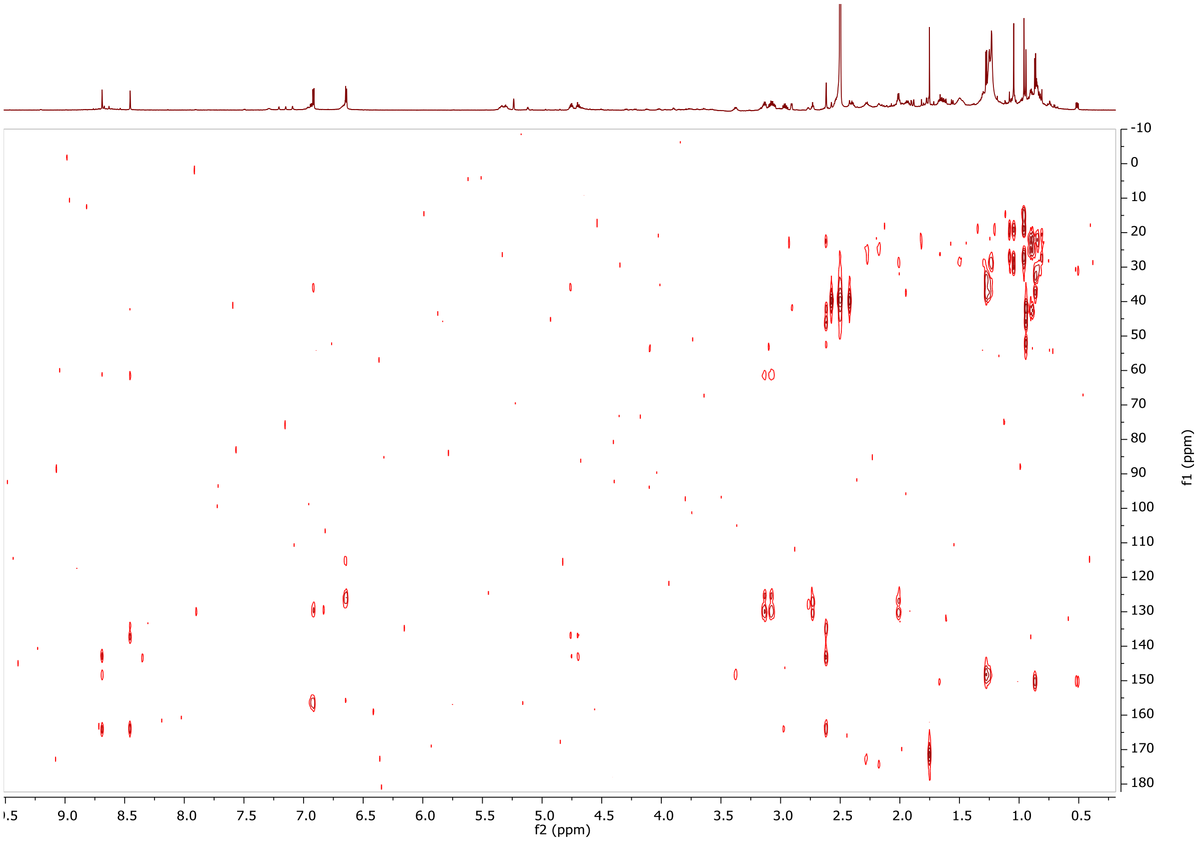

Figure S17 E: ^{1}H-^{13}C HMBC, DMSO-d_6 at 900 MHz data for valeraninium B.

The NMR Data of valeraninium C in DMSO-d_6					
Position	δ_H (J in Hz)	δ_C (C type)	Position	δ_H (J in Hz)	δ_C (C type)
1		126.1, C	1''	1.75 m	24.0, CH_2
2	6.97 d 8.4	129.6, CH		1.50 m	
3	6.66 d 8.4	115.1, CH	2''	2.15 m	36.9, CH_2
4		156.4, C		2.14 m	
5	6.66 d 8.4	115.1, CH	3''		128.6, C
6	6.97 d 8.4	129.6, CH	4''		134.4, C
7	3.13 m	35.7, CH_2	5''	3.46 m	32.5, CH
	3.06 m		6''	1.66 m	25.6, CH_2
8	4.76 m	61.3, CH_2		1.30 m	
	4.68 m		7''	1.78 m	27.8, CH_2
1'	8.71 s	137.9, CH		1.34 m	
2'		148.1, C	8''	1.93 m	32.5, CH
3'	3.38 m	37.4, CH	9''	2.87 m	46.6, CH
4'	2.42 m	33.1, CH_2	10''	1.61 s	13.1, CH_3
	1.68 m		11''	5.75 d 8.8	129.7, CH
5'	3.10 m	30.1, CH_2	12''		130.1, C
	2.98 m		13''	3.91 d 15.8	76.1, CH_2
6'		162.0, C		3.90 d 15.8	
7'		134.7, C	14''	1.67 s	13.6, CH_3
8'	8.69 s	140.5, CH	15''	0.74 d 7.0	11.7, CH_3
9'	1.26 d 6.6	18.7, CH_3	4-OH	9.34 s	
10'	4.51 d 17.2	65.1, CH_2			
	4.49 d 17.2				

Figure S18 A: NMR Data for valeraninium C in DMSO-d_6.

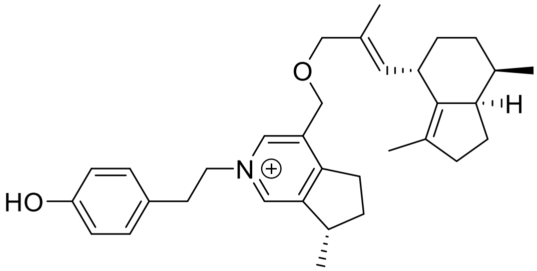

valeraninium C (^{1}H NMR, DMSO-d_6 at 900 MHz)

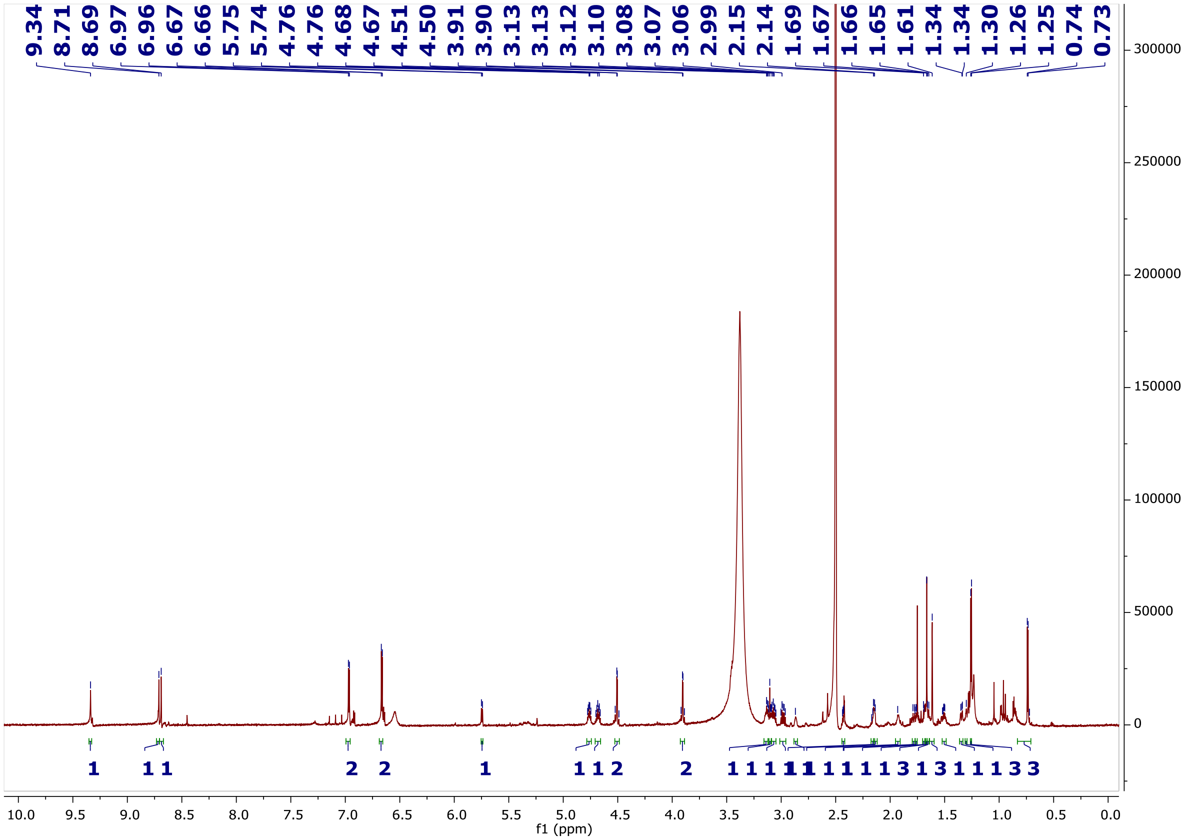

Figure S18 B: ^{1}H NMR, DMSO-d_6 at 900 MHz data for valeraninium C.

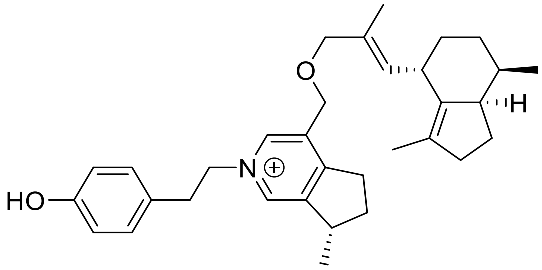

valeraninium C (^{1}H-^{13}C HSQC, DMSO-d_6 at 900 MHz)

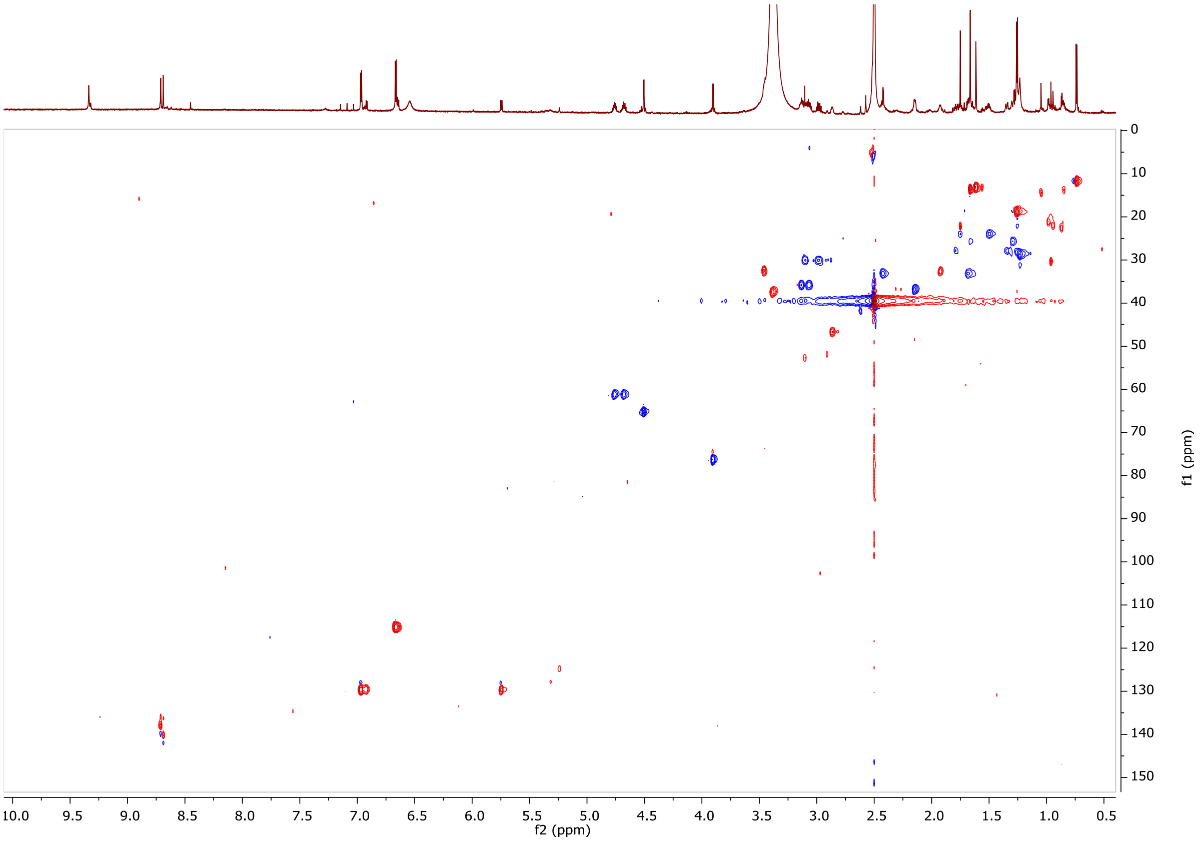

Figure S18 C: ^{1}H-^{13}C HSQC, DMSO-d_6 at 900 MHz data for valeraninium C.

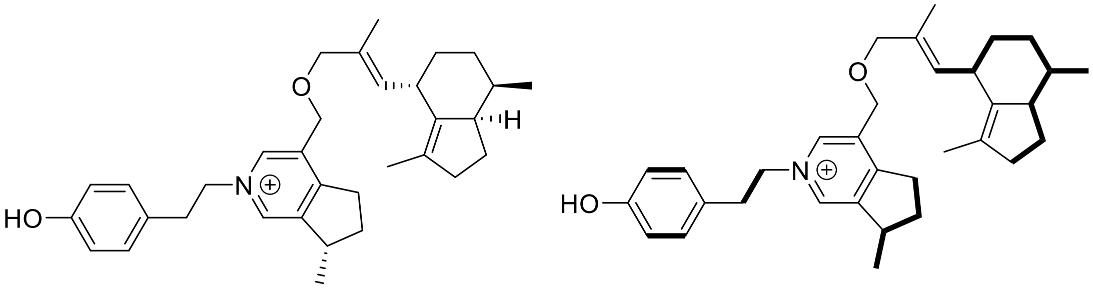

valeraninium C (^{1}H-^{1}H COSY, DMSO-d_6 at 900 MHz)

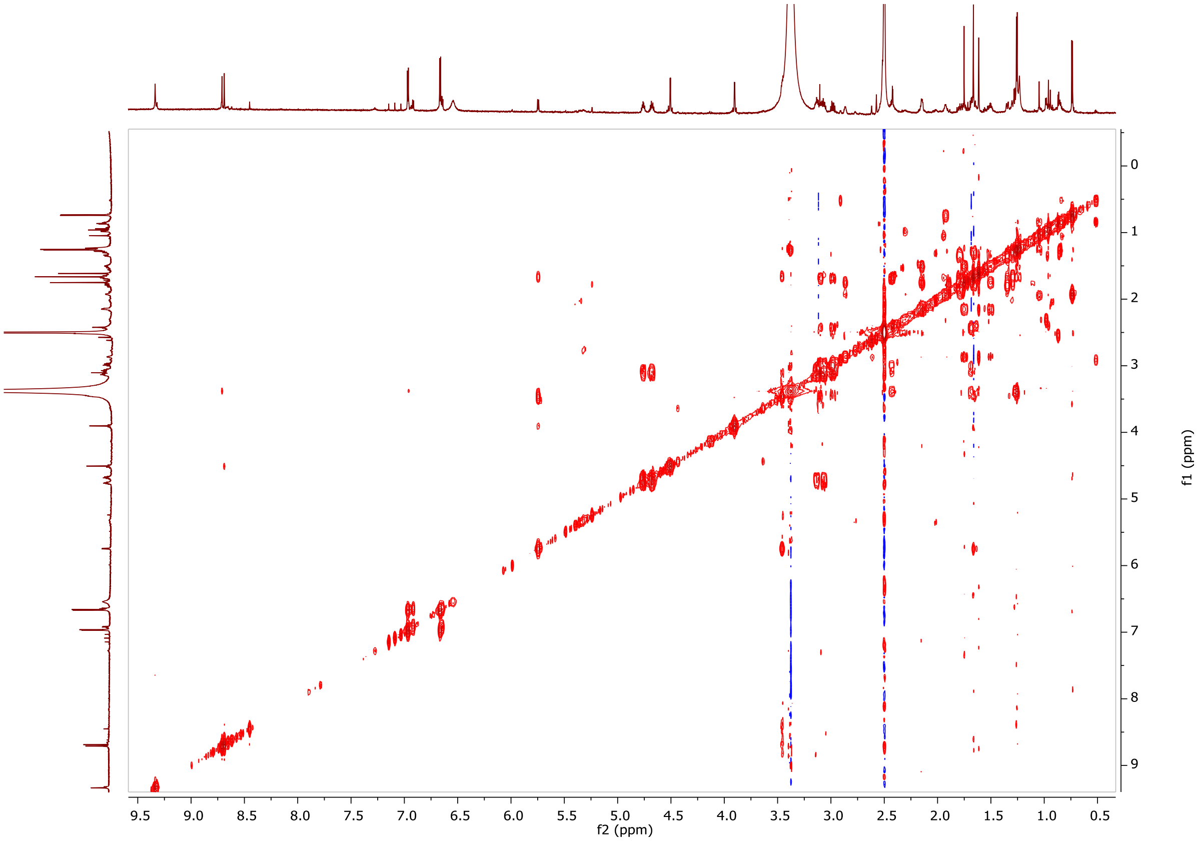

Figure S18 D: ^{1}H-^{13}C COSY, DMSO-d_6 at 900 MHz data for valeraninium C.

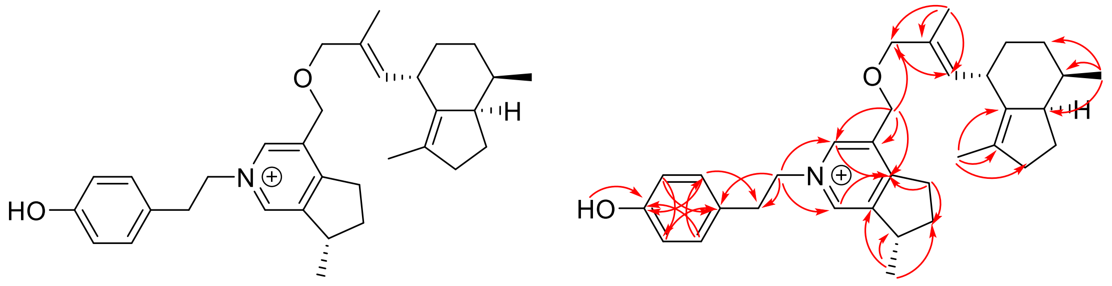

valeraninium C (^{1}H-^{13}C HMBC, DMSO-d_6 at 900 MHz)

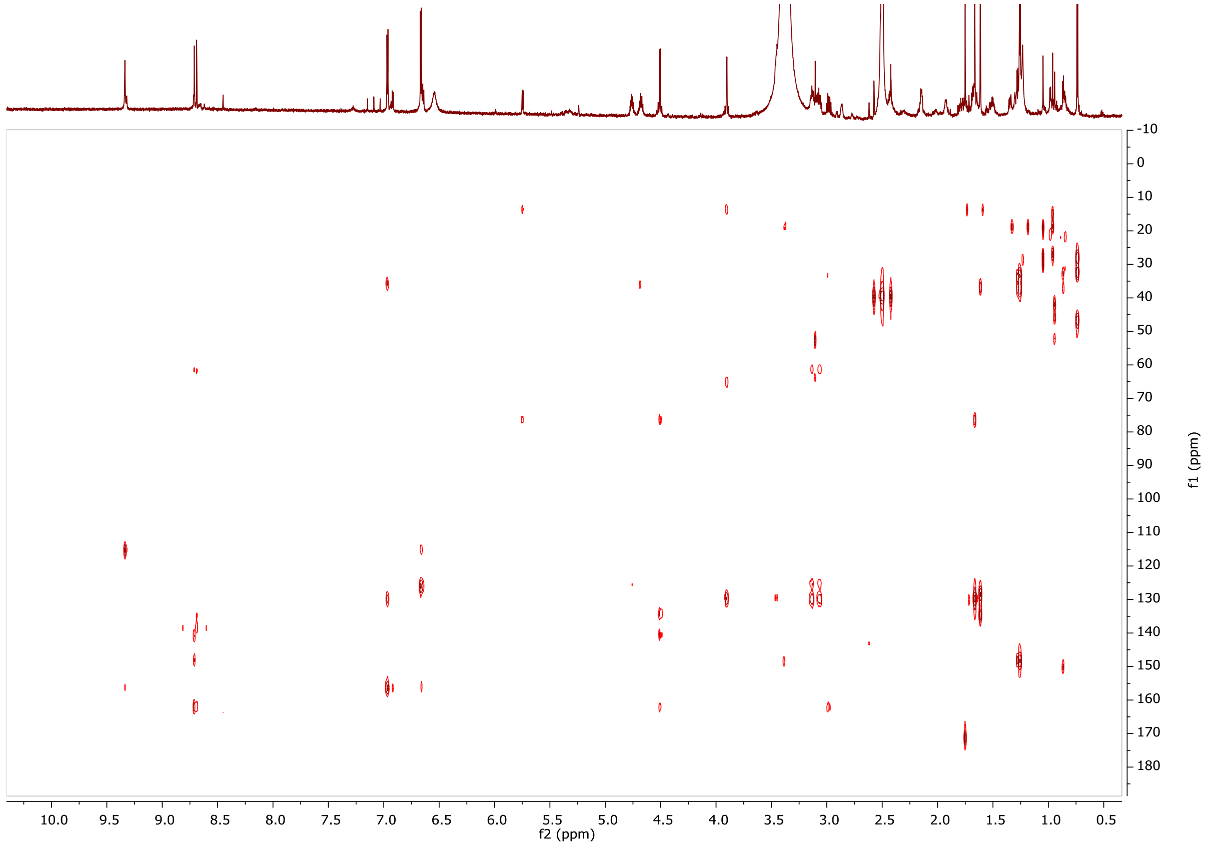

Figure S18 E: ^{1}H-^{13}C HMBC, DMSO-d_6 at 900 MHz data for valeraninium C.

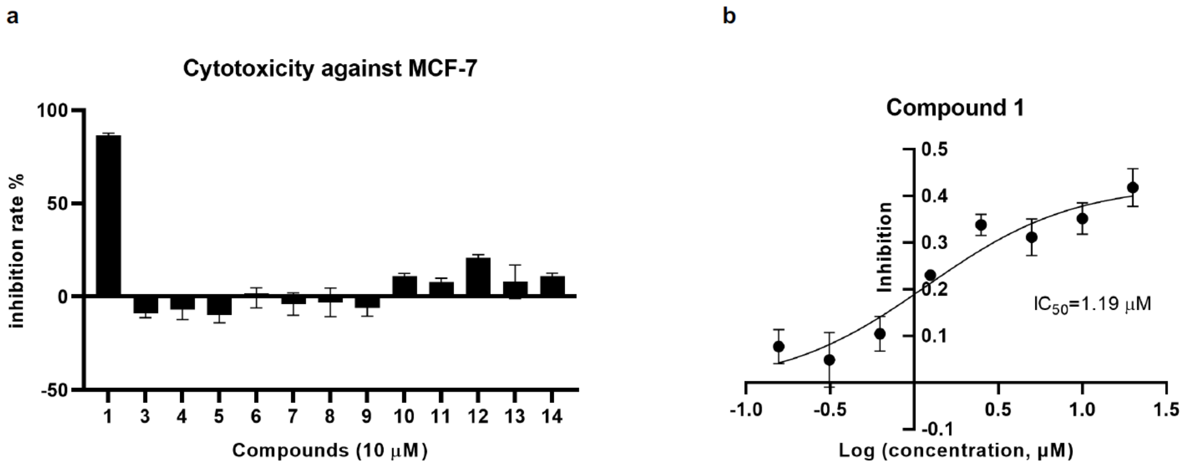

Figure S19: SRB assay results for isolated compounds in MCF-7 cells.

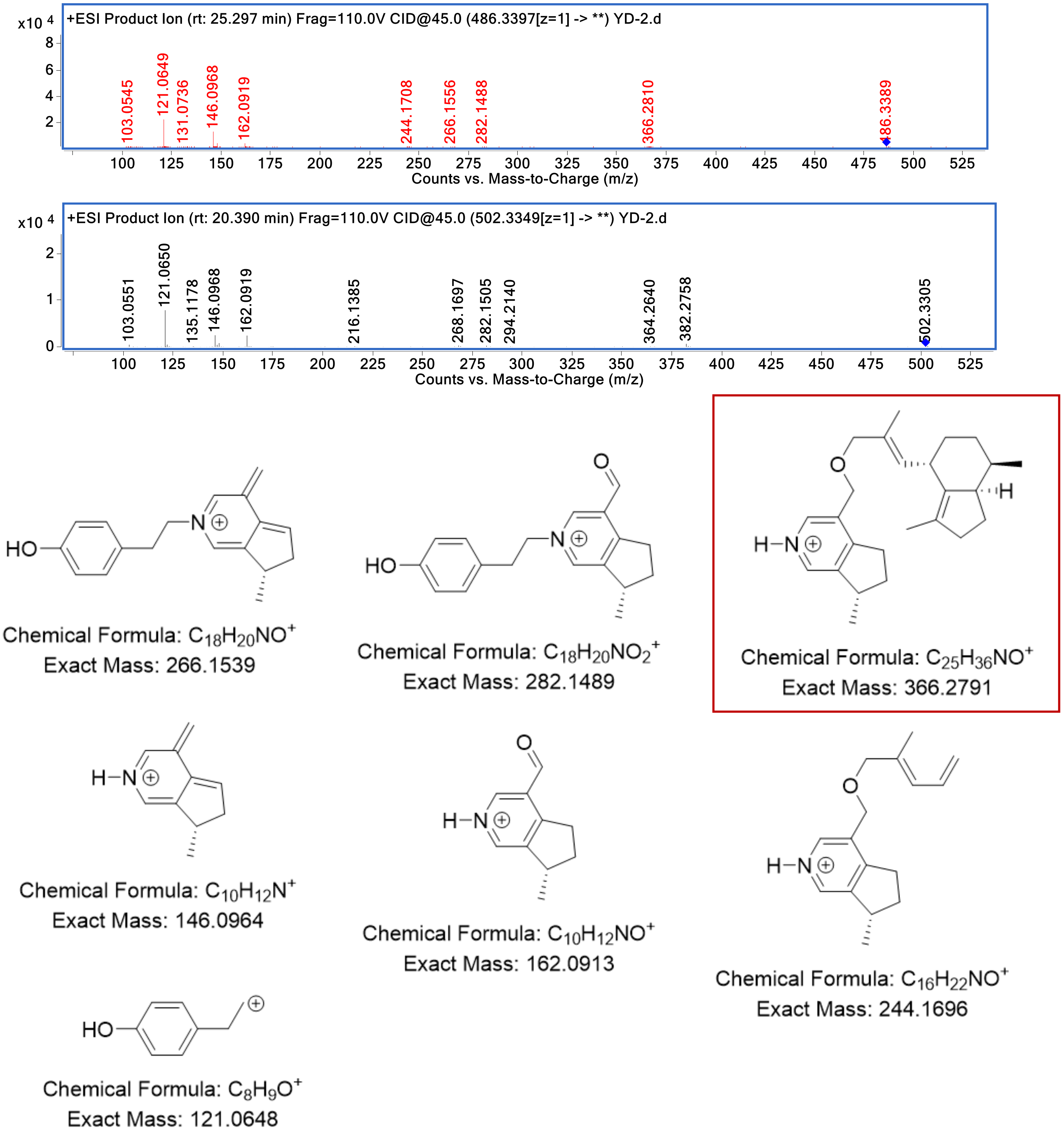

Figure S20: MS/MS fragmentation spectra for valeraninium C and its predicted variant along with assigned fragment structures. All fragment masses are equivalent, except for the fragment boxed in red. In the variant of valeraninium C, the exact mass for the equivalent fragment is 15.9948 m/z. This is nearly identical to the oxygen exact mass of 15.9949, showing that these two fragments are differentiated by the addition of a single oxygen atom.

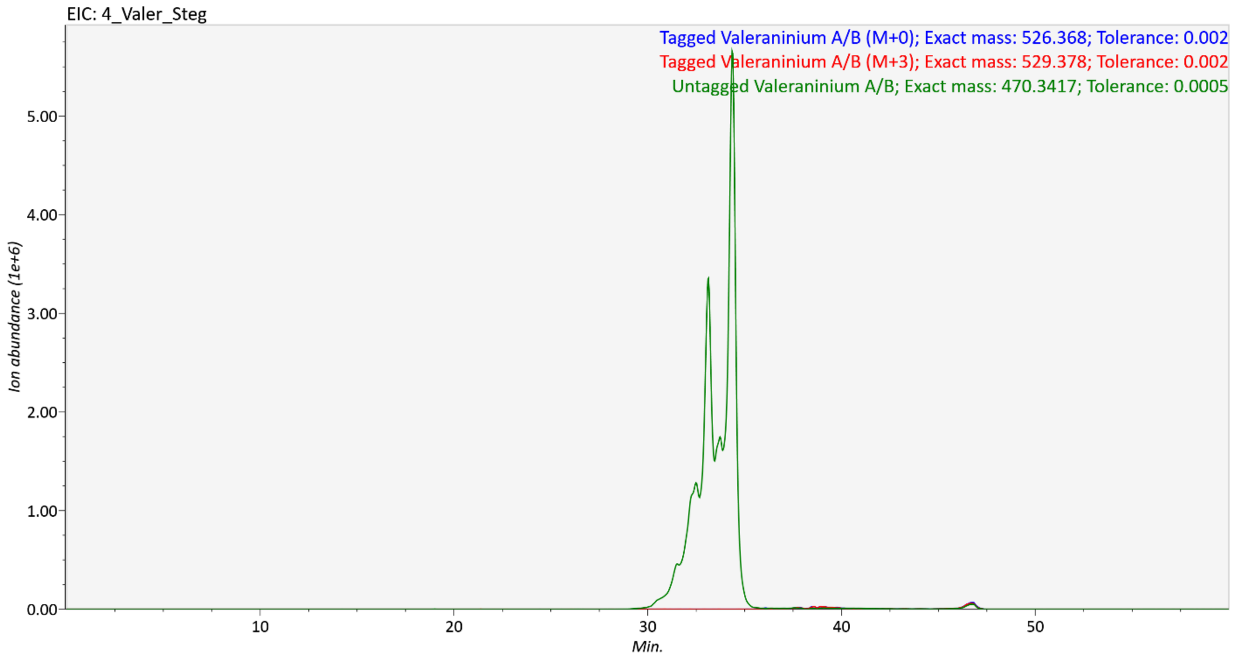

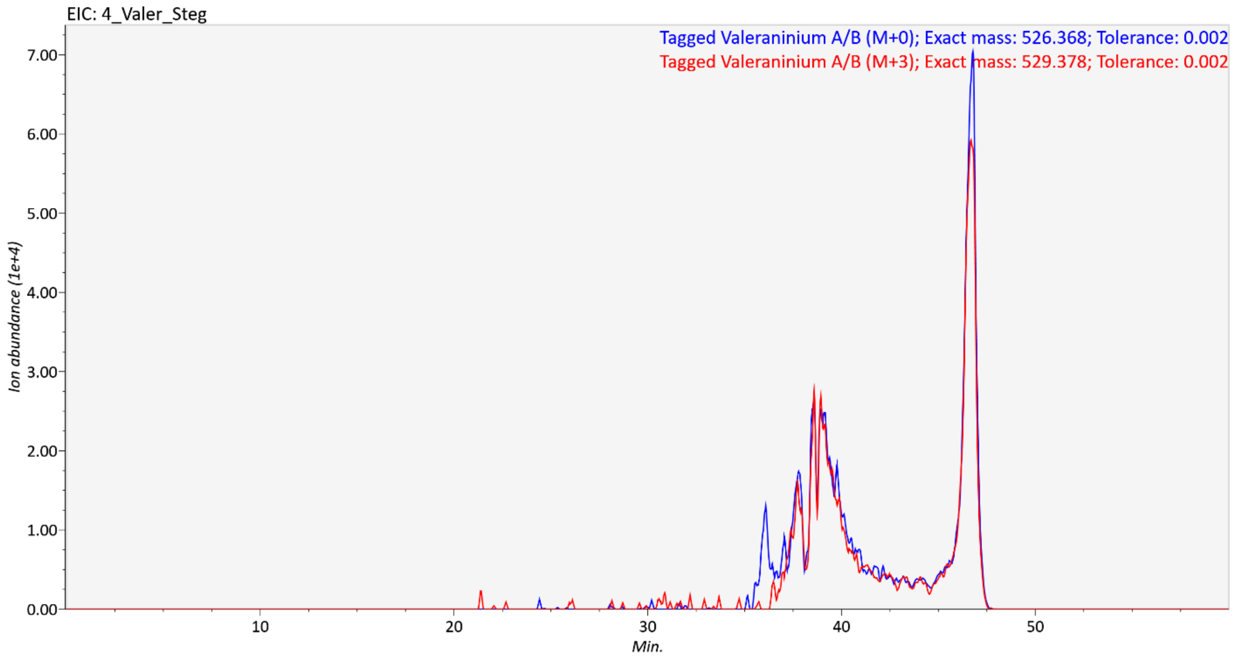

Figure S21 A: EIC Chromatograms for tagged and untagged ions derived from valeraninium A and B before the cell affinity assay. This result shows that the tagging efficiency was too low to observe the tagged masses after the cell binding assay.

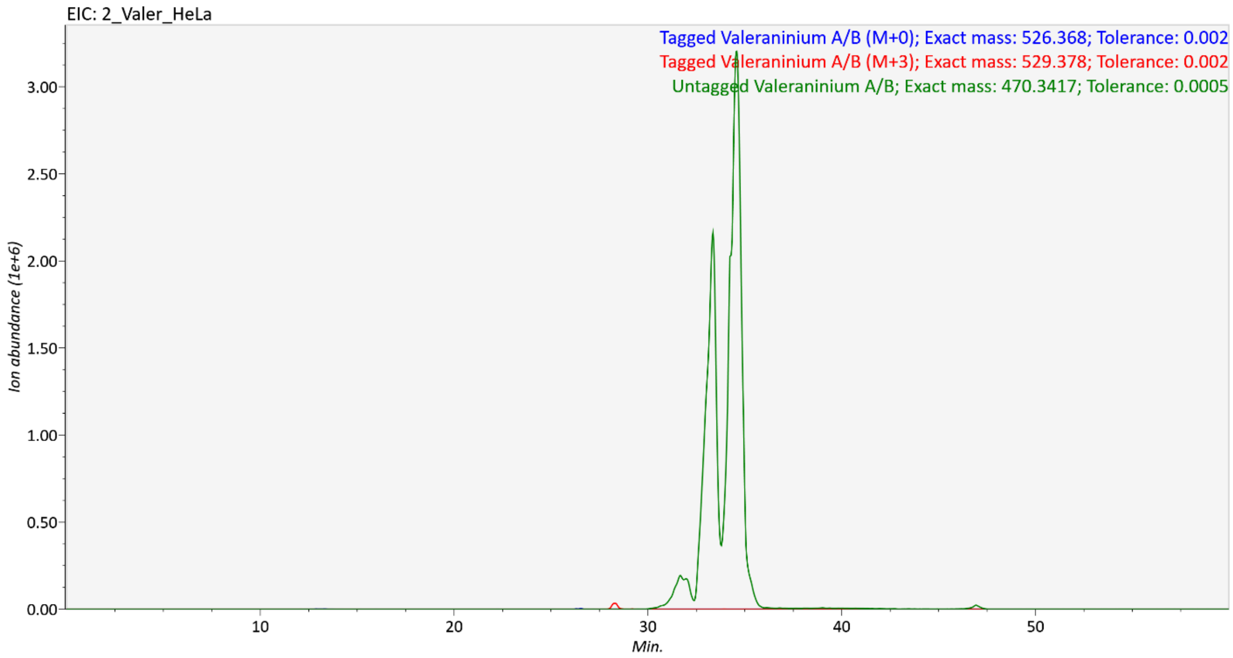

Figure S21 B: EIC Chromatograms for tagged and untagged ions derived from valeraninium A and B after the cell affinity assay. This result shows that the untagged ions are present after binding.

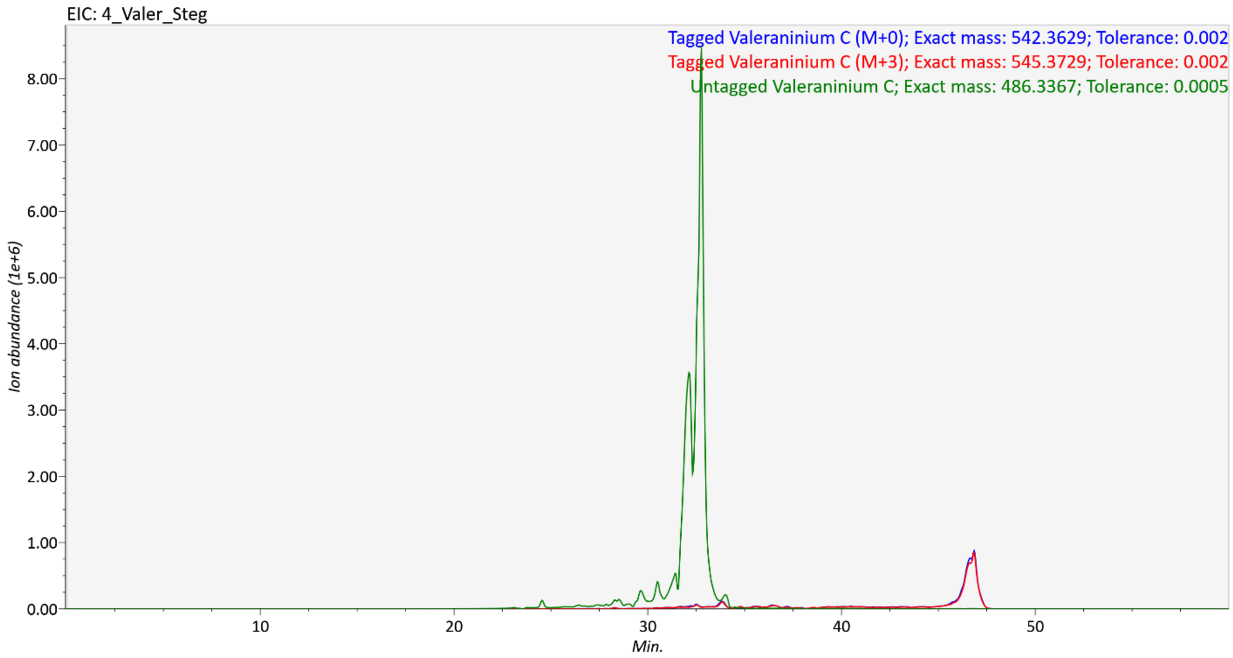

Figure S21 C EIC Chromatograms for tagged and untagged ions derived from valeraninium C before the cell affinity assay. This result shows that valeraninium C was successfully tagged during shotgun derivatization.

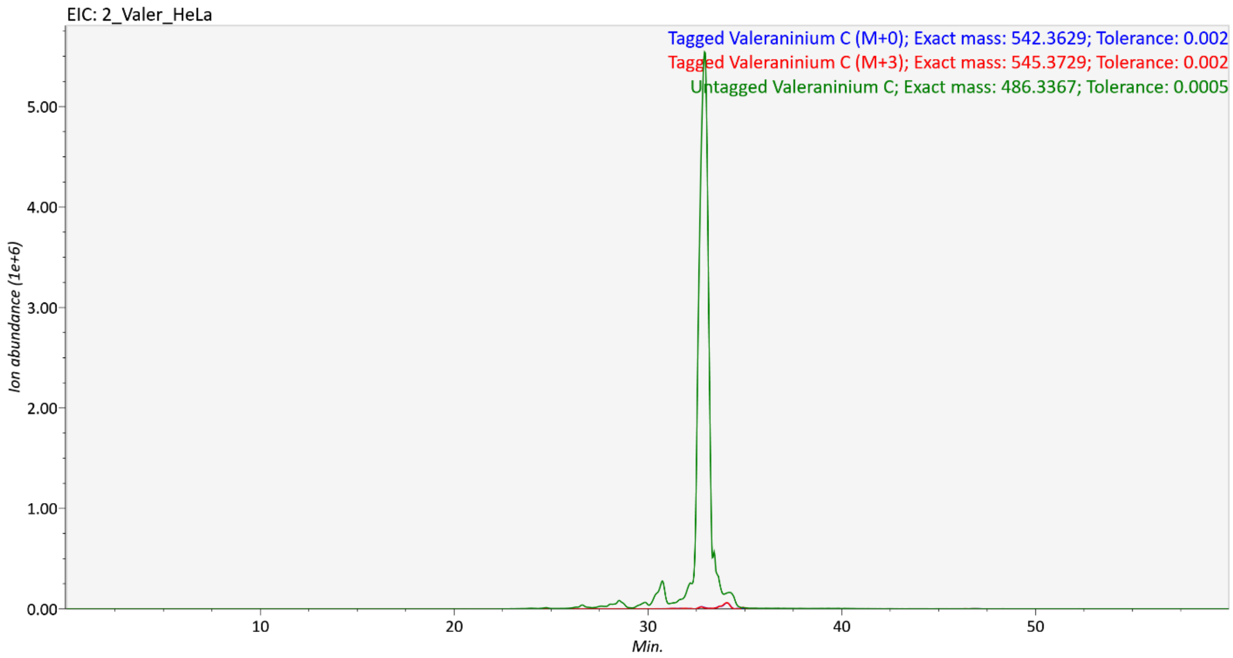

Figure S21 D: EIC Chromatograms for tagged and untagged ions derived from valeraninium C after the cell affinity assay. This result shows that only the untagged ion was present after the cell affinity assay. Additionally, the isomeric peak present RT=32 minutes is no longer present after the cell affinity assay.

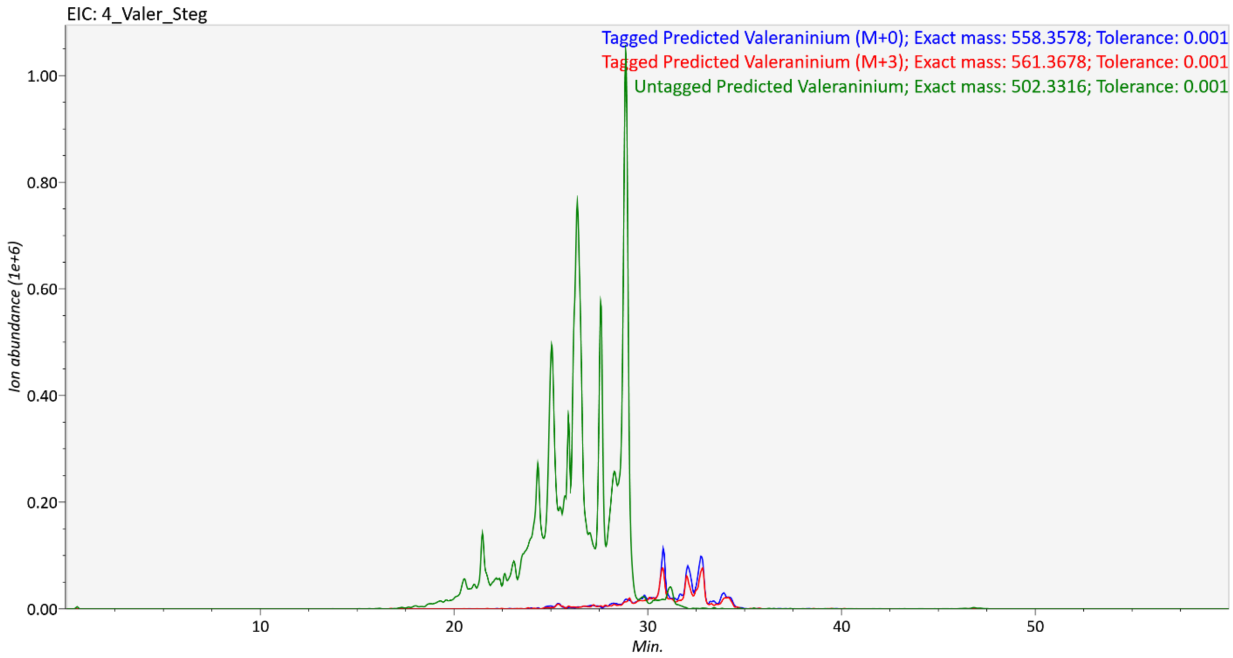

Figure S21 E: EIC Chromatograms for tagged and untagged ions derived from the predicted variant of valeraninium C before the cell affinity assay. This result shows that many isomers of this compound were present in both the tagged an untagged state.

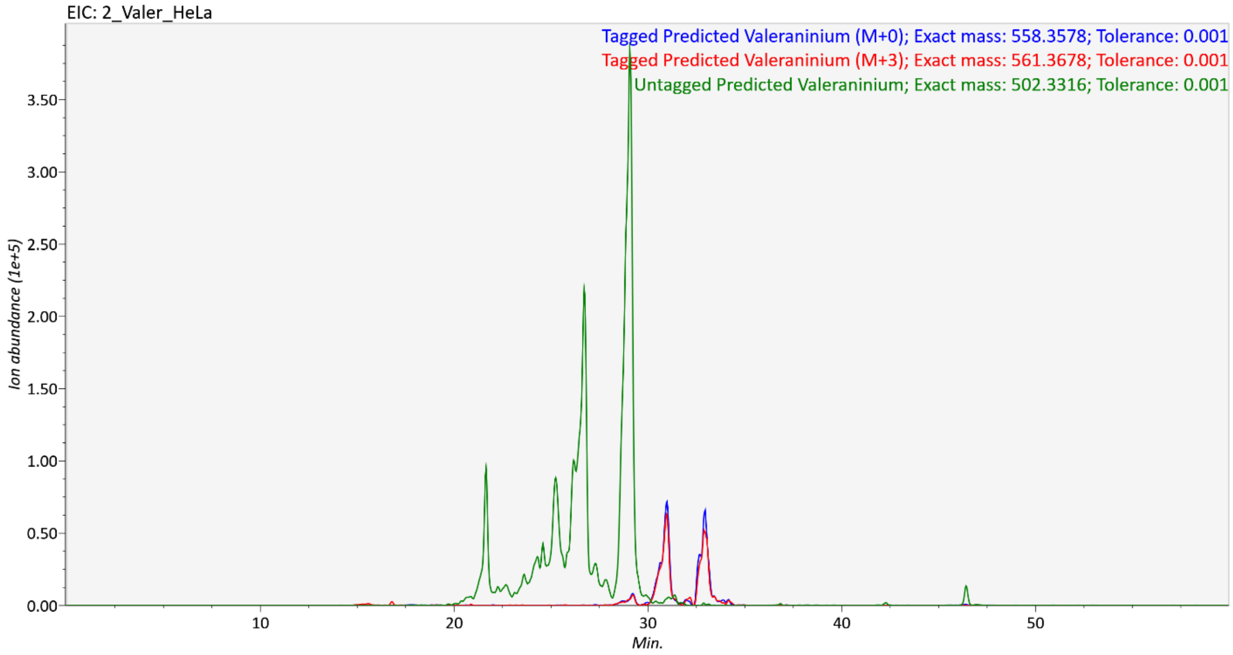

Figure S21 F: EIC Chromatograms for tagged and untagged ions derived from the predicted variant of valeraninium C after the cell affinity assay. This result shows that 4 out of 6 untagged isomers bound while only 2 out of 3 tagged isomers bound. The central peak of the tagged triad is notably missing.

Code for Mass Tag Search

```
import numpy as np
import pandas as pd
import matplotlib.pyplot as plt
from mpl_toolkits import mplot3d
```

In [2]:

```
def m3_search(w, data):

    '''This function searches for ions with [M+3] and correct intensity ratio
s within retention time tolerance'''

    # define columns of table as separate variables
    time = data[0:,0]
    mass = data[0:,1]
    intensity = data[0:,2]

    i = w
    j = 0
    n = len(time)

    dataMAT = []

   # create a hypothetical list of M+3 values from the data
    n = len(time)   # The length of my dataset
    m3guess = list()

    i = 0
    while i < n:
        m3 = mass[i] + 3*mC13
        m3guess.append(m3)
        i = i + 1

    # compare m3guess to mass

    w = 100
    i = w
    j = 0

    dataMAT = []

    while i < n - w:
        j = i - w
        while j < i + w:
            if mass[i] > m3guess[j] * (1 - error) and mass[i] < m3guess[j] *
(1 + error) and time[i] > time[j] + mintime and time[i] < time[j] + maxtime a
nd intensity[i] > intensity[j]*ratio1 and intensity[i] < intensity[j]*ratio2:
                dataMAT.append([mass[j], time[j], intensity[j], mass[i], time
[i], intensity[i] ])
                break
            else:
                j = j + 1
        i = i + 1
```

```
    data2 = pd.DataFrame(dataMAT,columns = ['mass 1','time 1','intensity 1','
mass 2','time 2','intensity 2'])

    return dataMAT
```

In [3]:

```
def result_crop(dataMAT, MZmin, RTmin, RTmax):

    '''This function crops the results list to include only masses and retent
ion times of potential value'''

    i = 0
    dataMAT2 = []

    while i < len(dataMAT):
        if  dataMAT[i][0] > MZmin and dataMAT[i][1] > RTmin and dataMAT[i][1]
 < RTmax:
            dataMAT2.append([dataMAT[i][0], dataMAT[i][1], dataMAT[i][2], dat
aMAT[i][3], dataMAT[i][4], dataMAT[i][5] ])
        i = i + 1

    data3 = pd.DataFrame(dataMAT2,columns = ['mass 1','time 1','intensity 1',
'mass 2','time 2','intensity 2'])

    return dataMAT2
```

In [4]:

```
#Keep only unique masses

def remove_duplicate_masses(dataMAT2):

    i = 0
    j = 1
    dataMAT3 = []

    while j < len(dataMAT2):
        if  dataMAT2[i][0] != dataMAT2[j][0]:
            dataMAT3.append([dataMAT2[i][0], dataMAT2[i][1], dataMAT2[i][2],
dataMAT2[i][3], dataMAT2[i][4], dataMAT2[i][5] ])
        i = i + 1
        j = j + 1

    dataMAT3.append([dataMAT2[i][0], dataMAT2[i][1], dataMAT2[i][2], dataMAT2
[i][3], dataMAT2[i][4], dataMAT2[i][5] ])

    data4 = pd.DataFrame(dataMAT3,columns = ['mass 1','time 1','intensity 1',
'mass 2','time 2','intensity 2'])

    return dataMAT3
```

In [5]:

```
#Remove M+1 Masses

def remove_m1_masses(dataMAT3):

    '''This function removes m+1 masses'''
```

```
    i = 0
    j = 1
    dataMAT4 = []

    dataMAT4.append([dataMAT3[0][0], dataMAT3[0][1], dataMAT3[0][2], dataMAT3
[0][3], dataMAT3[0][4], dataMAT3[0][5] ])

    while j < len(dataMAT3):
        if  dataMAT3[j][0] < (1-error)*(dataMAT3[i][0] + mC13) or dataMAT3[j]
[0] > (1+error)*(dataMAT3[i][0] + mC13):
            dataMAT4.append([dataMAT3[j][0], dataMAT3[j][1], dataMAT3[j][2],
dataMAT3[j][3], dataMAT3[j][4], dataMAT3[j][5] ])
        i = i + 1
        j = j + 1

    data5 = pd.DataFrame(dataMAT4,columns = ['mass 1','time 1','intensity 1',
'mass 2','time 2','intensity 2'])

    return dataMAT4
```

In [6]:

```
def complete_M3_search(w, data):

    dataMAT = m3_search(w, data)
    dataMAT2 = result_crop(dataMAT, MZmin, RTmin, RTmax)
    dataMAT3 = remove_duplicate_masses(dataMAT2)
    dataMAT4 = remove_m1_masses(dataMAT3)

    results = pd.DataFrame(dataMAT4,columns = ['mass 1','time 1','intensity 1
','mass 2','time 2','intensity 2'])

    return dataMAT4
```

In [7]:

```
def cross_ref(data_Steg, data_HeLa):

    # define columns of table as separate variables

    mass_Steg = data_Steg[0:,0]
    time_Steg = data_Steg[0:,1]
    intensity_Steg = data_Steg[0:,2]

    mass_HeLa = data_HeLa[0:,0]
    time_HeLa = data_HeLa[0:,1]
    intensity_HeLa = data_HeLa[0:,2]

    i = 0
    j = 0

    dataMAT5 = []

    while i < len(data_Steg):
        j = 0
        while j < len(data_HeLa):
            if  mass_Steg[i] > (1-error)*mass_HeLa[j] and mass_Steg[i] < (1+e
rror)*mass_HeLa[j] and time_Steg[i] < time_HeLa[j] + maxtime and time_Steg[i]
 > time_HeLa[j] + mintime:
```

```
                dataMAT5.append([mass_Steg[i], time_Steg[i], intensity_Steg[i
], mass_HeLa[j], time_HeLa[j], intensity_HeLa[j]])
                break
            else:
                j = j + 1
        i = i + 1

    cross_ref_result = pd.DataFrame(dataMAT5,columns = ['mass_Steg','time_Ste
g','intensity_Steg','mass_HeLa','time_HeLa','intensity_HeLa'])

    return dataMAT5
```

In [8]:

```
#define and edit constants

mC13 = (13.003355-12)*(3/3)  # mass C13 - mass C12 (amu)

ppm = 3
error = ppm/1000000

mintime = -(3/60)  # minutes
maxtime = (3/60)  # minutes

tag_ratio = 1
ratio1 = tag_ratio*(2/3)  # Peak 2 intensity/Peak 1 intensity lower bound
ratio2 = tag_ratio*(3/2)  # Peak 2 intensity/Peak 1 intensity upper bound

# define search window i+-w to save time without losing results
w = 100

#Keep only masses above minimum mass, and keep only RTs within range

MZmin = 250
RTmin = 10
RTmax = 40
```

In [9]:

```
# import dataset

Ashwa_Steg = np.genfromtxt('Ashwa_Steg.csv',delimiter=',')
Ashwa_HeLa = np.genfromtxt('Ashwa_HeLa.csv',delimiter=',')

HolyBa_Steg = np.genfromtxt('HolyBa_Steg.csv',delimiter=',')
HolyBa_HeLa = np.genfromtxt('HolyBa_HeLa.csv',delimiter=',')

Valer_Steg = np.genfromtxt('Valer_Steg.csv',delimiter=',')
Valer_HeLa = np.genfromtxt('Valer_HeLa.csv',delimiter=',')
```

In [12]:

```
#Process all data files individually
tag_ratio = 1
results_Ashwa_Steg = complete_M3_search(w, Ashwa_Steg)
results_Ashwa_HeLa = complete_M3_search(w, Ashwa_HeLa)

tag_ratio = 0.95
results_HolyBa_Steg = complete_M3_search(w, HolyBa_Steg)
results_HolyBa_HeLa = complete_M3_search(w, HolyBa_HeLa)
```

```
tag_ratio = 0.85
results_Valer_Steg = complete_M3_search(w, Valer_Steg)
results_Valer_HeLa = complete_M3_search(w, Valer_HeLa)
```

In [13]:

```
# Define and edit constants for crossreferencing

ppm = 4
error = ppm/1000000

mintime = -30/60  # minutes
maxtime = 30/60  # minutes
```

In [14]:

```
data_Steg = np.array(results_Ashwa_Steg)
data_HeLa = np.array(results_Ashwa_HeLa)
cross = cross_ref(data_Steg, data_HeLa)
print(len(data_Steg))
print(len(data_HeLa))
print(len(cross))
462
59
40
```

In [15]:

```
data_Steg = np.array(results_HolyBa_Steg)
data_HeLa = np.array(results_HolyBa_HeLa)
cross = cross_ref(data_Steg, data_HeLa)
print(len(data_Steg))
print(len(data_HeLa))
print(len(cross))
735
128
70
```

In [14]:

```
cross_ref_result = pd.DataFrame(cross,columns = ['mass_Steg','time_Steg','int
ensity_Steg','mass_HeLa','time_HeLa','intensity_HeLa'])
cross_ref_result
```

In [15]:

```
data_Steg = np.array(results_Valer_Steg)
data_HeLa = np.array(results_Valer_HeLa)
cross = np.array(cross_ref(data_Steg, data_HeLa))
print(len(data_Steg))
print(len(data_HeLa))
print(len(cross))
324
35
13
```

In [389]:

```
 cross_ref_result = pd.DataFrame(cross,columns = ['mass_Steg','time_Steg','in
tensity_Steg','mass_HeLa','time_HeLa','intensity_HeLa'])
```

In [390]:

```
cross_ref_result
```
